# Analysis of the Transcriptome of the Infective Stage of the Beet Cyst Nematode, *H*. *schachtii*

**DOI:** 10.1371/journal.pone.0147511

**Published:** 2016-01-29

**Authors:** John Fosu-Nyarko, Paul Nicol, Fareeha Naz, Reetinder Gill, Michael G. K. Jones

**Affiliations:** 1 Plant Biotechnology Research Group, Western Australian State Agricultural Biotechnology Centre, School of Veterinary and Life Sciences, Murdoch University, Perth, Australia; 2 NemGenix Pty Ltd, Western Australian State Agricultural Biotechnology Centre, Murdoch University, Perth, Australia; INRA, FRANCE

## Abstract

The beet cyst nematode, *Heterodera schachtii*, is a major root pest that significantly impacts the yield of sugar beet, brassicas and related species. There has been limited molecular characterisation of this important plant pathogen: to identify target genes for its control the transcriptome of the pre-parasitic J2 stage of *H*. *schachtii* was sequenced using Roche GS FLX. Ninety seven percent of reads (i.e., 387,668) with an average PHRED score > 22 were assembled with CAP3 and CLC Genomics Workbench into 37,345 and 47,263 contigs, respectively. The transcripts were annotated by comparing with gene and genomic sequences of other nematodes and annotated proteins on public databases. The annotated transcripts were much more similar to sequences of *Heterodera glycines* than to those of *Globodera pallida* and root knot nematodes (*Meloidogyne* spp.). Analysis of these transcripts showed that a subset of 2,918 transcripts was common to free-living and plant parasitic nematodes suggesting that this subset is involved in general nematode metabolism and development. A set of 148 contigs and 183 singletons encoding putative homologues of effectors previously characterised for plant parasitic nematodes were also identified: these are known to be important for parasitism of host plants during migration through tissues or feeding from cells or are thought to be involved in evasion or modulation of host defences. In addition, the presence of sequences from a nematode virus is suggested. The sequencing and annotation of this transcriptome significantly adds to the genetic data available for *H*. *schachtii*, and identifies genes primed to undertake required roles in the critical pre-parasitic and early post-parasitic J2 stages. These data provide new information for identifying potential gene targets for future protection of susceptible crops against *H*. *schachtii*.

## Introduction

The beet cyst nematode, *H*. *schachtii*, is a sedentary endoparasitic plant nematode present in temperate and mediterranean regions. It has a narrow host range but can substantially impact infected crops, with losses of 30% common in hosts of the families *Chenopodiaceae* (especially *Beta vulgaris*) and *Cruciferae* [1, 2]. Heavy root infestation with *H*. *schachtii* can cause root distortion, ‘bearding’ of sugar beet storage roots and lateral root death: plants become prone to wilting and there is a reduction in plant growth and yield. Plant parasitic nematodes (PPNs) are usually managed by a combination of crop rotation, application of nematicidal agrochemicals and deployment of natural resistance genes, and each of these has limitations. In particular, nematicides are either too expensive, toxic over the longer term, some have been banned or their use restricted, and there are few non-hosts to use as break crops in crop rotations. In many cases resistant genes are not available or effective against PPNs [3–5]. These factors make the search for alternative control methods for nematodes more imperative. *H*. *schachtii* is of additional interest because it is one of the few PPNs which can infect *Arabidopsis thaliana*, the best understood model plant species [6].

**Fig 1:**
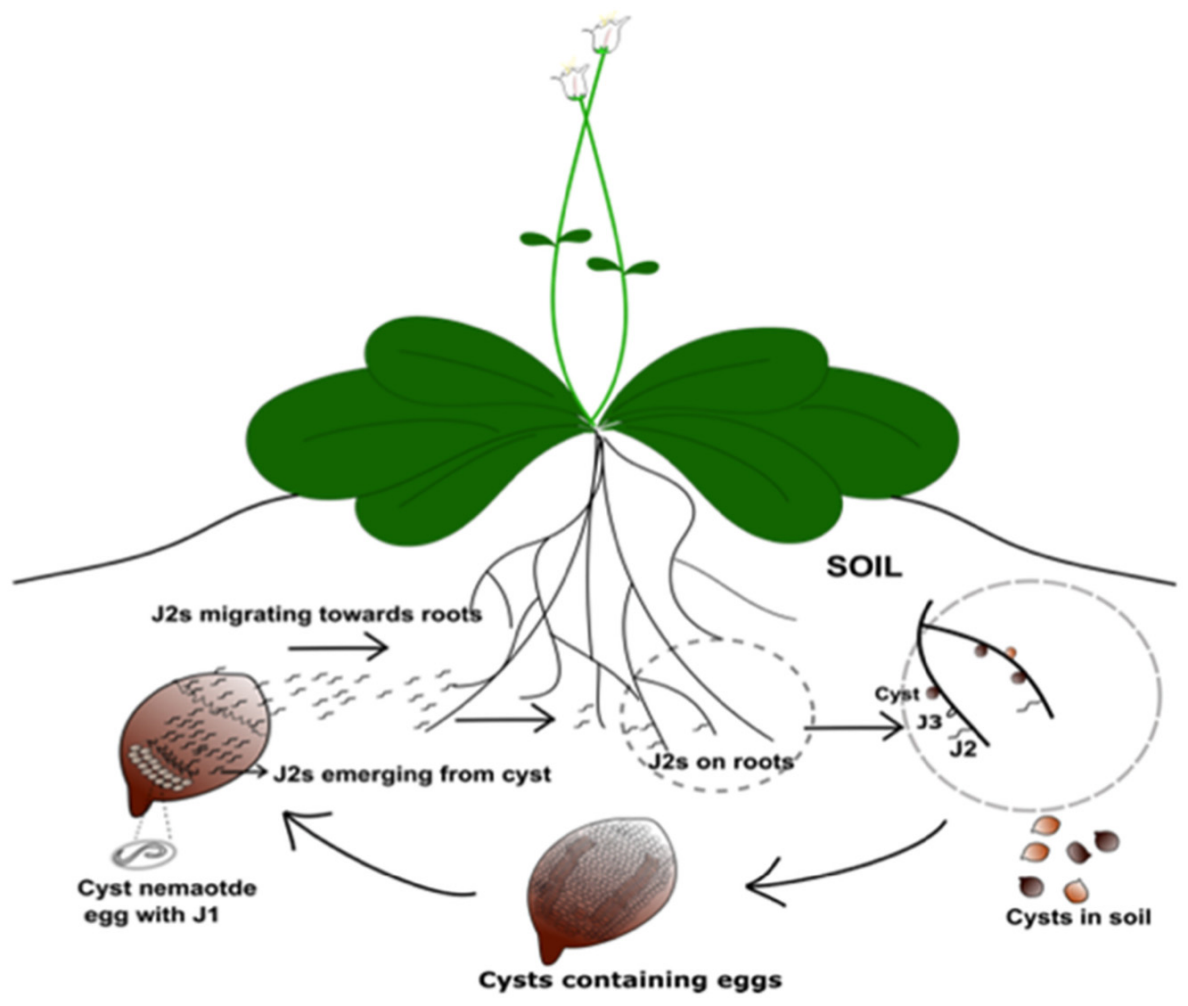
Life cycle of the beet cyst nematode, *Heterodera schachtii*.

Pre-parasitic cyst nematode stage 2 juveniles (J2s) moult from J1s that develop from eggs within the cysts, and then migrate to host plants (Fig 1). They then enter root cells using their hollow mouth stylet, aided by secretions produced from gland cells that include cell wall-modifying enzymes such as cellulases and pectinases, and migrate intracellularly, responding to positional gradients in the root [7–9]. In a susceptible host, the nematode becomes established after forming a typical feeding site (syncytium). The syncytium forms through dissolution of cell walls and cell expansion to create a multinucleate region of interconnected and metabolically active cells, from which it feeds throughout its life cycle [10–12]. Secretions from J2s are required for root entry and migration, and in initiation and establishment of a syncytium by co-ordinated modification of expression of a series of host genes. Once a syncytium has been initiated successfully, J2s become sedentary, and each feeds from its associated syncytium, growing and developing via three further moults to maturity. Males are associated with smaller syncytia—they emerge from roots, and fertilise the females. Adult females become lemon-shaped and after fertilisation, produce many eggs, most of which are retained within the body. When the female nematode dies the body wall tans and hardens to form a protective cyst around the eggs.

Genetic studies of *H*. *schachtii* have been limited so far to targeted isolation of transcripts/genes of putative effectors to study the parasitome (i.e. secreted products of parasitism genes) by direct amplification, cloning and sequencing [8, 13, 14]. Recent advances in sequencing technology and bioinformatic analysis platforms have led to increased understanding of nematode biology. For example, the genomic sequencing and characterisation of genes of the free-living *Caenorhabditis* spp. has contributed to annotation of sequenced genomes of animal parasitic nematodes and PPNs such as the root knot nematodes *Meloidogyne hapla* and *Meloidogyne incognita* [15, 16], the cyst nematode *G*. *pallida* [17] and recently the migratory endoparasitic nematode *Pratylenchus coffeae* [18]. In addition, global transcriptomic methods allow complex sets of genes and pathways in an organism to be analysed simultaneously, so that in the case of nematodes it can be used to study the expression of genes at different life stages, including genes involved in parasitism (e.g. [19–22]). At present there are only 3,066 nucleotide sequences for *H*. *schachtii* in the largest sequence database, the National Centre for Biotechnology Information (NCBI) Genbank (http://www.ncbi.nlm.nih.gov/) and 1,592 on nematode.net (http://nematode.net), and a global transcriptome analysis has not been published so far for any stage of the nematode.

In this paper, we report the sequencing of the transcriptome of the infective J2 stage of *H*. *schachtii* prior to root entry, and annotation of transcripts using sequences of both free-living and parasitic nematodes, and conserved or core eukaryotic genes. Several bioinformatic tools were used to identify a set of genes that may be primed for host infection. The infective J2 stage appears to express many of the genes required for migration through host cells and for initiation of syncytia. Transcripts with homologies to the recently identified soybean cyst nematode nyavirus were also found [23]. Analysis of the transcriptome of J2s of *H*. *schachtii* provides an additional resource for functional genetic analysis of this PPN. Although many of the identified genes need further functional characterisation, it also provides a set of sequences that could be used to exploit gene-based strategies such as RNA interference, to develop new strategies for control of this economically important nematode.

## Materials and Methods

### Ethics Statement

The population of *H*. *schachtii* used for the study was originally obtained from a cabbage and broccoli farm north of Perth, Western Australia. Permission to enter the farm was granted by the farm owner who also kindly provided soil samples from a freshly harvested plot of land from which we obtained mature female cysts. No specific regulatory permissions were required for these locations/activities because *H*. *schachtii* is an endemic pest in Western Australia, and these nematodes are not protected or an endangered species.

### Transcriptome sequencing and assembly of reads

The population of *H*. *schachtii* used for transcriptome sequencing was derived from a single female and was maintained on cabbage plants grown in white sand in a glasshouse at 25°C (day) and 15°C (night). Pre-parasitic J2s were harvested using a mist apparatus described by Tan et al [24] (S1 Fig). Using this method, it was expected that the pre-parasitic J2s extracted, which had been exposed to roots of susceptible hosts, were primed for infection, and the genes expressed at this stage were analysed. For J2 nematode isolation, soil taken from the root zone of infected plants was placed in a 200 mL plastic container lined with two layers of coffee filters. The soil was sprayed with a water mist every 10 minutes for 10 seconds, and active nematodes migrated through the double coffee filter and were collected at four hourly intervals in aerated fresh water that was continually gently replaced to remove microbial contamination (S1 Fig). To further prevent contamination, the active freshly extracted nematodes were then surface-sterilised by suspension in 1% chlorhexidine gluconate (hibitane) for 20 min, followed by washing with 1% streptomycin for 2 min and then three washes with sterile distilled water: for each treatment and wash the nematodes were harvested by gentle centrifugation at 1200 g for 2 minutes. The J2s were then examined by light microscopy to ensure they were viable and free from any visible microbial contamination before RNA extraction. Total RNA was extracted with Trizol (Invitrogen Life Technologies, Carlsbad, USA) and cleaned using a RNeasy Mini kit column (Qiagen Pty. Ltd., Victoria, Australia). The quality and quantity of RNA was assessed with an Agilent 2100 Bioanalyzer (Agilent Technologies, Mississauga, Canada) and RNA with a minimum RNA Integrity Number (RIN) of 7 and with absorbance ratios of 260:280 at 2.0 and 260:230 at 2.1 were used for cDNA synthesis. A cDNA library was prepared from 3 μg of total RNA using the Ovation RNA-Seq system (NuGEN Technologies Inc., CA, USA), which uses both Oligo-dT and random primers, and checked for removal of rRNA using an Agilent 2100 Bioanalyzer. Sequencing (half a picotitre plate) was carried out using a Roche 454 GS FLX DNA platform at the Institute of Immunology and Infectious Diseases, Murdoch University, Perth, Australia.

Reads that passed the key filter test based on default parameters for base calling were trimmed and analysed after quality control. The average PHRED scores for the reads (mean of PHRED scores for bases making up an entire read) were then determined using the CLC Genomics Workbench 7.0.4 after which high quality reads were assembled with the CAP3 assembler and the CLC Genomics Workbench 7.0.4. CAP3 was configured to run with an overlap per cent identity cut-off of 90, overlap length cut-off of 40, mismatch score factor of -5 and other default settings [25]. The set of parameters used for the CLC Genomics Workbench assembly were: mismatch cost of 2, insertion cost of 2, deletion cost of 2, length fraction of 0.4 and similarity fraction of 0.4.

### Annotation and functional classification of transcripts

For most analyses TBLASTX 2.2.30+ [26] was used to compare transcripts to reference sequences in defined NCBI databases or local databases (e.g. created for CLC Genomics Workbench 7.0.4). All BLAST analyses were done with a threshold e-value of 1E-05 after which hits with significant identities were sorted or manually curated using High-scoring Segment Pair (HSP) scores or total bit scores as these parameters are independent of the search space size and enable comparisons across databases and searches. Multiple sequence alignments of transcripts and matches presented were done using BioEdit [27]. Transcripts were annotated based on similarities with sequences in the NCBI non-redundant (nr/nt), Gene Ontology (GO, submission date (30/9/2014, geneontology.org/page/download-annotations) [28], and Carbohydrate Active Enzyme (CAZy, November, 2014 release, www.ahv.dk) [29] databases. In particular, they were compared to protein sequences of well-characterised free-living nematodes (*Caenorhabditis* spp.) to determine genes expressed in the *H*. *schachtii* J2 transcriptome and their putative functions using the GO classification, based on *C*. *elegans* genes. Transcripts for proteins involved in RNA interference (RNAi) pathways were also identified using those characterised for *C*. *elegans*: these proteins were identified from the literature and from descriptions on Wormbase (www.wormbase.org): their amino acid sequences were downloaded and used as a reference for BLASTX searches. Term identifiers for PAMGO, Plant Associated Microbe Gene Ontology, were used to determine association of transcripts with known plant host-pathogen interactions [30].

### Comparative analyses with ESTs and genes of other nematodes

The *H*. *schachtii* J2 transcriptome was compared to genes and ESTs of a reference group of 17 nematode species available at NCBI using a local TBLASTX/BLASTX with a threshold e-value of 1E-05. The reference nematode groups used were the free-living nematodes *C*. *elegans* and *Caenorhabditis remanei*, and three PPN groups with different modes of feeding: cyst nematodes (*H*. *glycines*, *H*. *schachtii*, *G*. *pallida*, *Globodera mexicana*, *Globodera rostochiensis*), the root knot nematodes *Meloidogyne* spp: *M*. *arenaria*, *M*. *chitwoodi*, *M*. *javanica*, *M*. *paranaensis*, *M*. *incognita*, *M*. *hapla*) and migratory nematodes (*Pratylenchus vulnus*, *Pratylenchus penetrans*, *Pratylenchus thornei* and *Radopholus similis*). The numbers of ESTs, genomic contigs and protein sequences for these nematode species as well as sequences in databases used for functional annotation (GO, CAZy, PAMGO) are provided in S1 Table.

### Mapping *H*. *schachtii* transcriptome to genomes of four PPNs

The *H*. *schachtii* transcriptome was also compared with publicly available genomic contigs of two cyst [*H*. *glycines* (PRJNA28939) and *G*. *pallida* (PRJEB123)] and two root knot nematodes [*M*. *hapla* (PRJNA29083) and *M*. *incognita* (PRJEA28837)], both directly and indirectly in four ways. BLASTN and TBLASTX were first used separately to study relatedness of the transcripts to those for putative proteins encoded by the genomic sequences. The *H*. *schachtii* reads were also mapped onto the genomic contigs of each of the four nematodes using the “map to reference” function of CLC Genomics Workbench 7.0.4 with a mismatch cost of 2, an insertion cost of 3, a deletion cost of 3, a length fraction of 0.5 and a similarity fraction of 0.8. Finally, genes expressed at the pre-parasitic stage were assessed using the Core Eukaryotic Genes (CEGs) used in CEGMA (Core Eukaryotic Genes Mapping Approach [31]), and were also compared to CEGs encoded by genomic sequences of the four plant parasitic nematodes.

### Identification of spliceosome genes and evidence of trans-splicing in *H*. *schachtii*

Further analysis was conducted with the transcriptome to study genes involved in the spliceosome of cyst nematodes, in particular, the phenomenon of trans-splicing. *H*. *schachtii* orthologues of such genes were identified using the KEGG spliceosome pathway of *C*. *elegans* as a reference (www.genome.jp/kegg). Evidence of trans-splicing in *H*. *schachtii* was studied using the presence of splice leader (SL) sequences on transcripts. A SL1 RNA gene was first identified from genomic sequences of *H*. *glycines* (PRJNA28939) using BLASTN and sequences for those of *C*. *elegans* and *Pristionchus pacificus* [32] to confirm the presence of such sequences in genomes of cyst nematodes. The identified SL sequence was then compared to trans-splice leaders (SL1-like and SL2-like) of 11 nematode species (including *Pristionchus pacificus*, *Brugia malayi*, *Ascaris suum*, *C*. *elegans* and *C*. *briggsae)* [33] which identified it as a SL1-like sequence. This conserved SL1-like sequence was then used to identify similar sequences in *H*. *schachtii* transcripts.

### Putative nematode parasitism effectors of *H*. *schachtii*

Transcripts for putative parasitism effectors of *H*. *schachtii* were identified using functionally characterised and putative homologues of other PPNs. A local database was created for representative characterised parasitism effectors of cyst, root knot and migratory PPNs and used as a reference for TBLASTX search among the *H*. *schachtii* transcripts. The sequences included those for 30 characterised effectors (29 complete coding (mRNA) and one partial cDNA) and all publicly available ESTs isolated from gland cells and secretions of *H*. *glycines* and *M*. *incognita*. Alignments of best matching transcripts, with the highest bit score, to respective reference sequence(s) were manually curated, for example, to allow identification of the presence of important features such as signal peptides. Also, sequences of transcripts with matches to nematode parasitism effectors and CAZymes were translated into six frames using the translate programme in CLC Genomics Workbench 7.0.4 and the putative proteins of each frame used as a query to search for signal peptides and trans-membrane helices similar to those of eukaryotes using SignalP 4.1 [34].

## Results

### The transcriptome

**Fig 2:**
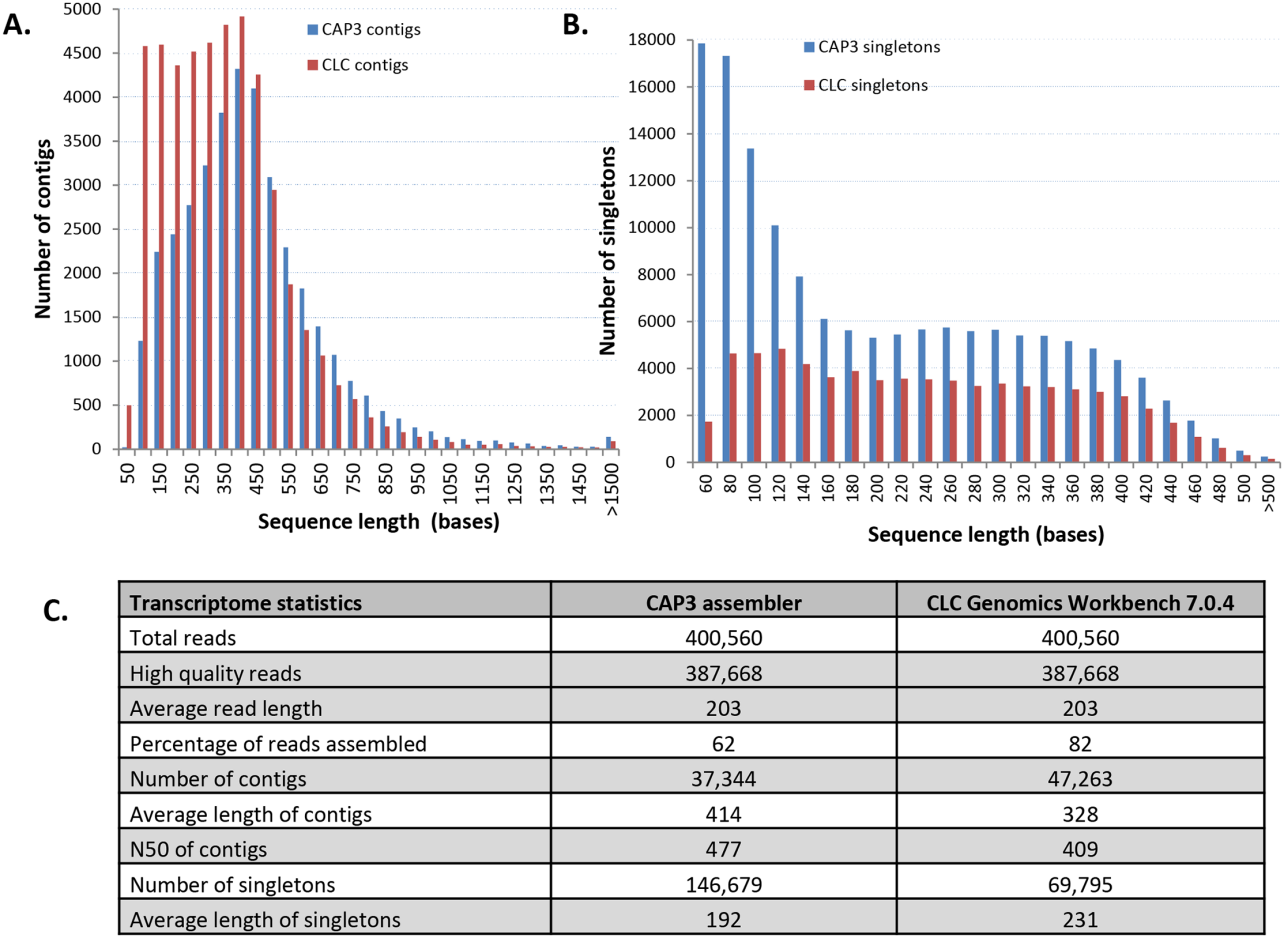
Reads and assembly data of the *H*. *schachtii* transcriptome. A). Size distribution of contigs assembled with CAP3 and CLC Genomics Workbench 7.0.4. B). Size distribution of singletons after read assembly with CAP3 and CLC Genomics Workbench 7.0.4. C). Transcriptome and assembly statistics of CAP3 and CLC Genomics Workbench 7.0.4.

The sequencing generated a total of 1,064,983 reads of which 400,622 reads passed key filter tests and were further analysed. From this, 387,668 (97%) high quality reads with a least average PHRED score of 22 were assembled into contigs. From 82% of the high quality reads, the CLC Genomics Workbench generated 47,263 contigs, about 10,000 more than CAP3, which assembled 62% into contigs (Fig 2). Consequently, there were more singletons after the CAP3 assembly, with an average length of 192 nt, which was about 40 bases shorter than those from the CLC Genomics Workbench assembly (Fig 2). The average length and N50 of the CAP3 contigs were both slightly greater: 414 nucleotides (nt) and 477 nt respectively. The total length of the CAP3 and CLC contigs were 35.4% and 19.7%, respectively, of the entire length of all reads of the transcriptome although more reads were assembled by the CLC genomics workbench. For both assemblies, the majority of the contigs were between 300–600 nt long: 60.7% for CAP3 and 52.5% for the CLC Genomics Workbench contigs (Fig 2). The longest contigs for both assemblies were over 4 Kb long (one for each). Generally for the CAP3 assembly there were substantially fewer contigs 100 bases or shorter than from the CLC assembly, with the opposite the case for singletons (Fig 2). Because the CLC Genomics Workbench assembled more reads into contigs and the average length of singletons was longer, the CLC assembly was used for all annotation of the transcriptome. Analysis of available ESTs of other cyst nematodes in the NCBI databases indicate the average length and the N50 for the CLC assembly were both shorter: respectively the average length and N50 for ESTs of the reference nematodes were 368 and 578 for *H*. *schachtii*; 267 and 611 for *Globodera* spp and, 355 and 691 for *H*. *glycines*. The generated reads have been deposited in the Sequence Read Archive of NCBI under the accession SRX381021.

### Gene ontology of transcripts

From comparisons with the NCBI non-redundant protein dataset, 87.8% of the annotated transcripts matched eukaryotes whereas 8.9% matched bacterial proteins, 0.5% to viral sequences and the remainder matched archaea, and unclassified sequences. The transcripts matched sequences from 343 species; most of these had high percentage sequence identities to transcripts/genes of *Caenorhabditis spp*, *A*. *suum*, *Loa loa* and *B*. *malayi*. Transcripts from PPNs were relatively under-represented in the results either because there were fewer sequences in databases or the ESTs were shorter than those for the free-living and animal species.

**Fig 3:**
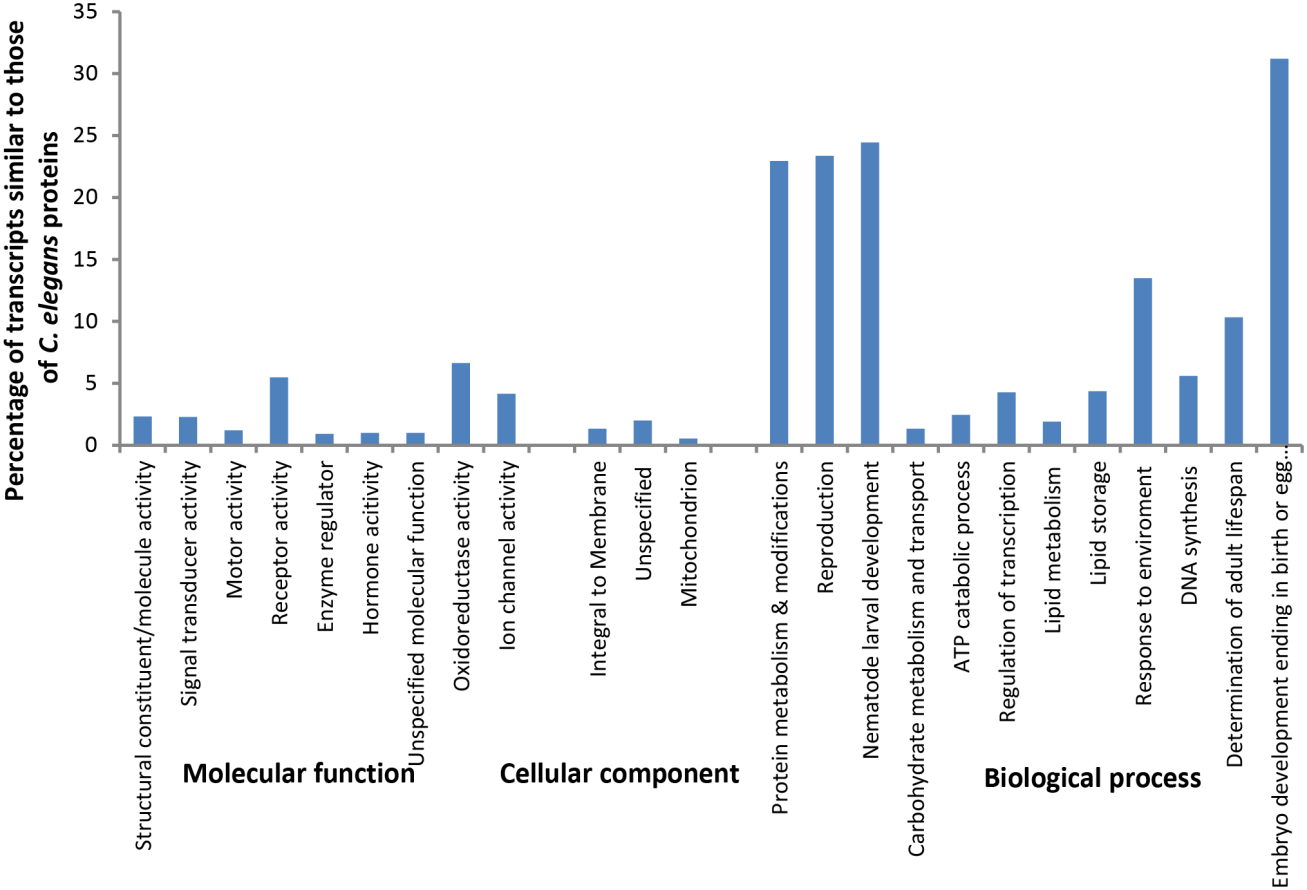
Gene ontology of *H*. *schachtii* transcripts inferred from *C*. *elegans* homologues.

The GO classification scheme based on proteins of *Caenorhabditis* spp. was used to annotate the transcripts. A total of 6,430 transcripts (3,579 contigs and 2,851 singletons) had similarities to those of 2,530 *C*. *elegans* proteins. Using the GO of 2,410 *C*. *elegans* proteins for detailed functional classification of the transcripts, 1,915 were involved in biological processes, 42 were associated with cellular components and 1,587 had various molecular functions (Fig 3). A total of 46 *H*. *schachtii* transcripts with sequence similarities to those of nine proteins associated with PAMGO terms were identified (S2 Table). These included three proteins for *Phytophthora* spp and six for *Magnaporthe grisea*, seven of which were associated with multiple PAMGO terms including the following: ‘pathogenesis’ (GO:0009405), ‘entry into host’ (GO:0044409), ‘chemotaxis’ (GO:0006935), and ‘secretion/toxin transport activity’ (GO:0019534). While some of these terms could be related to activities of a nematode infection process, functions of the associated transcripts and for those associated with molecular terms/functions involved in development (e.g. the GO:0004674-Serine/threonine protein kinase activity) and interaction with a compatible host (e.g. GO:0007165-signal transduction), are yet to be determined for most nematodes.

### Comparative analysis with ESTs and genes of nematode species

**Fig 4:**
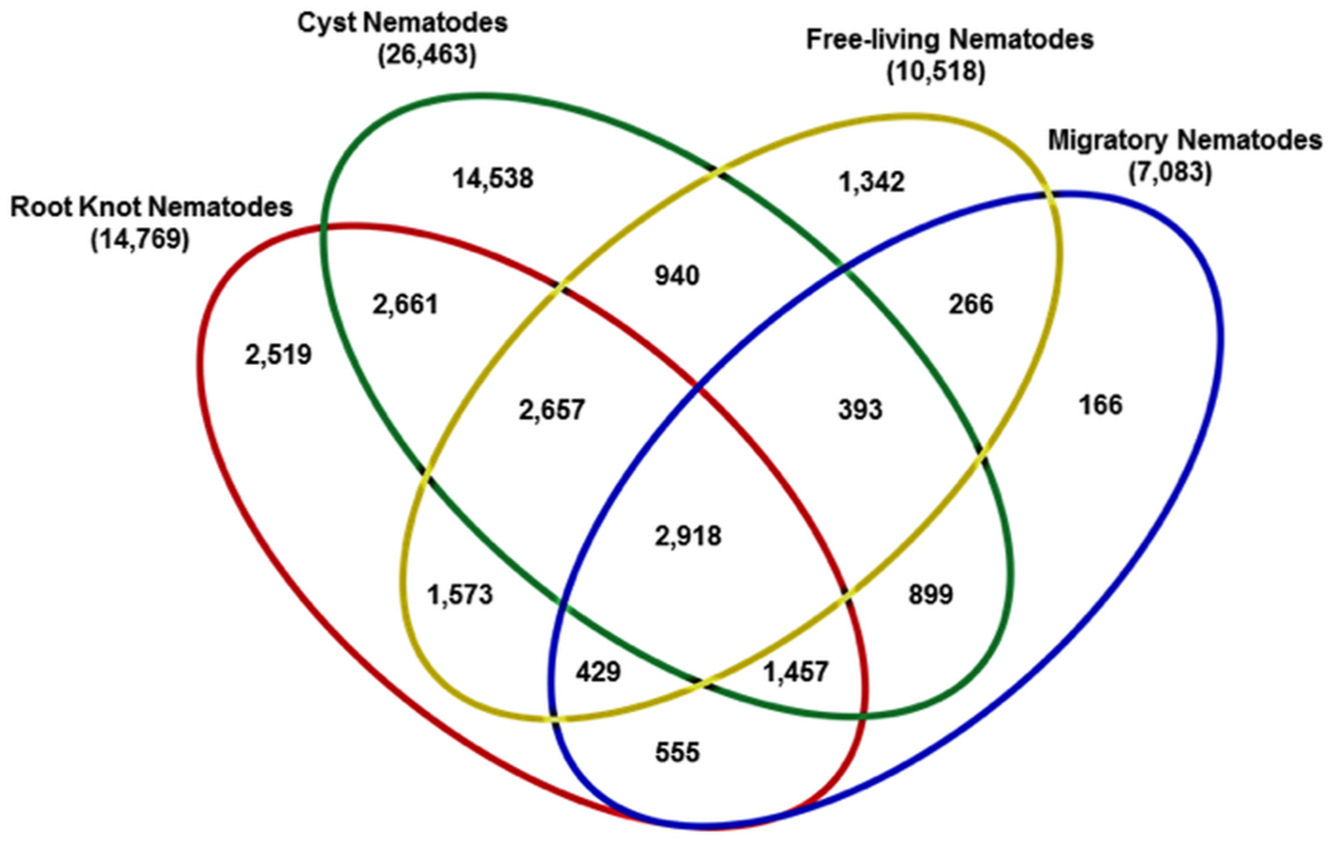
Distribution of *H*. *schachtii* transcripts amongst genes/ESTs of nematodes of different lifestyles.

When the transcripts were compared to genes and ESTs of free-living, sedentary cyst and root knot, and migratory endoparasitic nematodes, those identified with sequence similarity to any of the reference group were collated and presented in a Venn diagram (Fig 4). A total of 33,313 had sequence similarities to ESTs/genes of the reference nematode groups of which 2,918 were common to all the four reference groups of nematodes (Fig 4). Consistent with the number of publicly available reference sequences used for the comparisons, more *H*. *schachtii* transcripts matched ESTs of the cyst nematode group (26,464), followed by the root knot group (14,769) and then the free-living (10,518) and the migratory endoparasitic nematodes with 7,083 matched transcripts. The numbers of *H*. *schachtii* transcripts with matches to only sequences of each group followed a similar pattern: more for the cyst nematode group, then the root knot, followed by the free living and migratory nematode groups in that order (Fig 4). Most of the 2,918 transcripts which match sequences of all the four group of nematodes putatively encode proteins involved in developmental and common molecular and biological processes of eukaryotes. Not surprisingly, transcripts with no match to any sequence of free-living nematodes included those putatively encoding parasitism effectors.

### The transcriptome of *H*. *schachtii* and genomes of four PPNs

**Fig 5:**
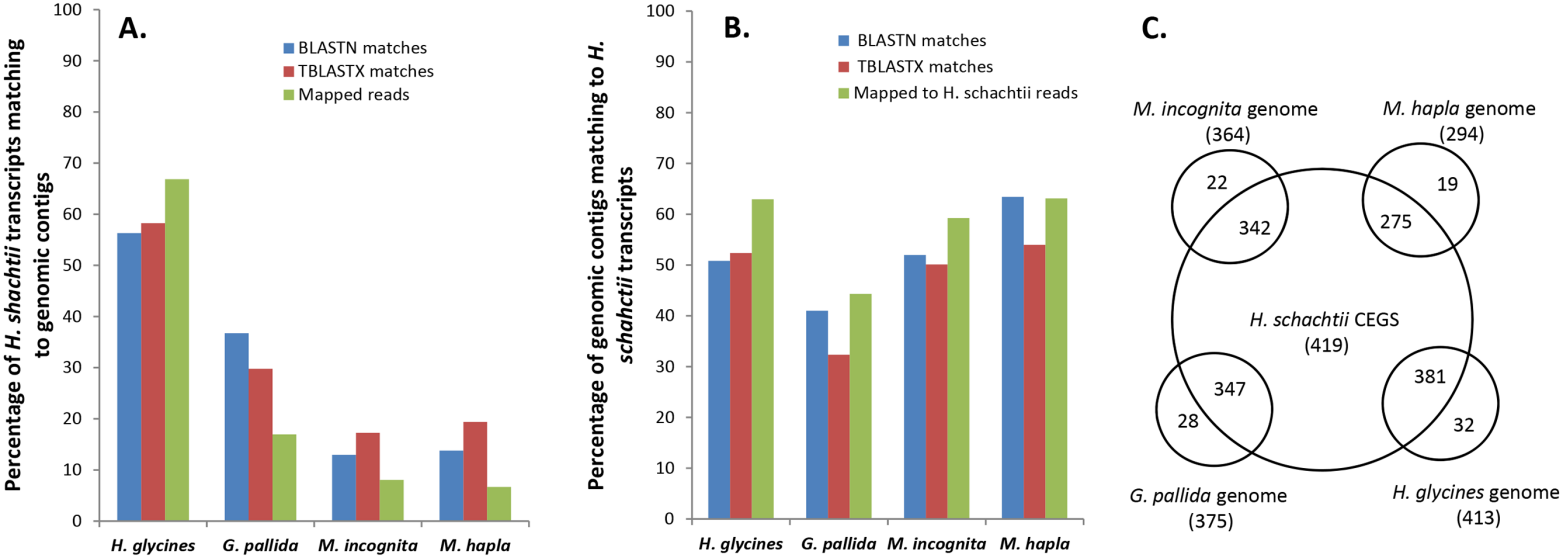
Comparative analysis of *H*. *schachtii* transcripts with genomes of four PPNs. A). Percentage of *H*. *schachtii* transcripts with matches to genomic contigs of *H*. *glycines*, *G*. *pallida*, *M*. *incognita* and *M*. *hapla*. B). Percentage of genomic contigs of *H*. *glycines*, *G*. *pallida*, *M*. *incognita* and *M*. *hapla* with similarity to *H*. *schachtii* transcripts. C). Comparison of homologues of CEGs in the *H*. *schachtii* transcriptomes to those in genomes of *H*. *glycines*, *G*. *pallida*, *M*. *incognita* and *M*. *hapla*.

BLASTN and TBLASTX comparisons revealed 58% of the *H*. *schachtii* transcripts mapped to 52% of genomic contigs of the closely related *H*. *glycines* at an e-value threshold of 1E-05, whereas in all 43% of the transcripts from both the BLASTN and TBLASTX searches matched 41% of the *G*. *pallida* genomic contigs (Fig 5A and 5B). About 20% of the transcripts matched contigs of the root knot nematodes. However, the matching contigs represented close to 63% and 52% of the total genomic contigs for *M*. *hapla* and *M*. *incognita* respectively (Fig 5A and 5B). Using the ‘map reads to reference’ function on the CLC Genomics Workbench, more (ca 67%) *H*. *schachtii* reads were mapped to *H*. *glycines* contigs (63%) than to contigs of *G*. *pallida*, *M*. *hapla* and *M*. *incognita* (Fig 5A) and like the BLAST results, the percentage of genomic contigs mapped to the *H*. *schachtii* reads were higher for *H*. *glycines*, *M*. *hapla*, *M*. *incognita* than for *G*. *pallida* (Fig 5B).

The 259,111 reads that mapped to the *H*. *glycines* contigs had an average length of 192 nt and made up 63.25% of the total bases of the transcriptome. Generally, the number of reads mapping to the genomic contigs did not depend on the read lengths or the composition of the reads since the distribution of the total, mapped and unmapped reads were similar. Moreover, the number of mapped reads was not dependent on the length of genomic contigs, but reflected the abundance of particular reads in the transcriptome (*p<0*.*05*). For example, whereas 265 reads mapped onto *H*. *glycines* genomic contig ABLA01000003.1 (21602 nt), only 56 mapped to contig ABLA01000001.1 (24,040 nt). On average 9 reads mapped to the *H*. *glycines* contigs with a range of 1 to 5,901 and with an average read consensus length of 603 nt. The read mapping could clearly be used to delineate intron-exon boundaries of the genomic sequences. For example, *H*. *glycines* genomic contig with the longest matched read consensus, contig ABLA01000003, contains genes putatively encoding four *C*. *elegans* homologues namely dynein heavy chains (*dhc-1*, *che-3*), ribosomal protein subunit 31 (*rpl-31*) and deoxyuridine triphosphate nucleotidohydrolase (*dut-1*).

A total of 454 out of the 458 Core Eukaryotic Genes (CEGs) were identified altogether in the J2 transcriptome of *H*. *schachtii* and in the genomic contigs of the four PPNs: of these 256 were common. For the *H*. *schachtii* transcriptome, about 2% of transcripts (1149 contigs and 905 singletons) were similar to a total of 419 CEGs. Varying numbers of genomic contigs of the four PPNs identified from BLAST comparisons appear to encode CEGs; 15.3% for *M*. *hapla*, 12% for *M*. *incognita*, 3.1% for *H*. *glycines* and 5.2% for *G*. *pallida* (Fig 5C). Relatively more CEGs were identified in the transcriptome and the genome of *H*. *glycines* than the other genomes and these two species also had more CEGs in common (Fig 5C).

### The spliceosome of *H*. *schachtii*: evidence of trans-splicing

**Fig 6:**
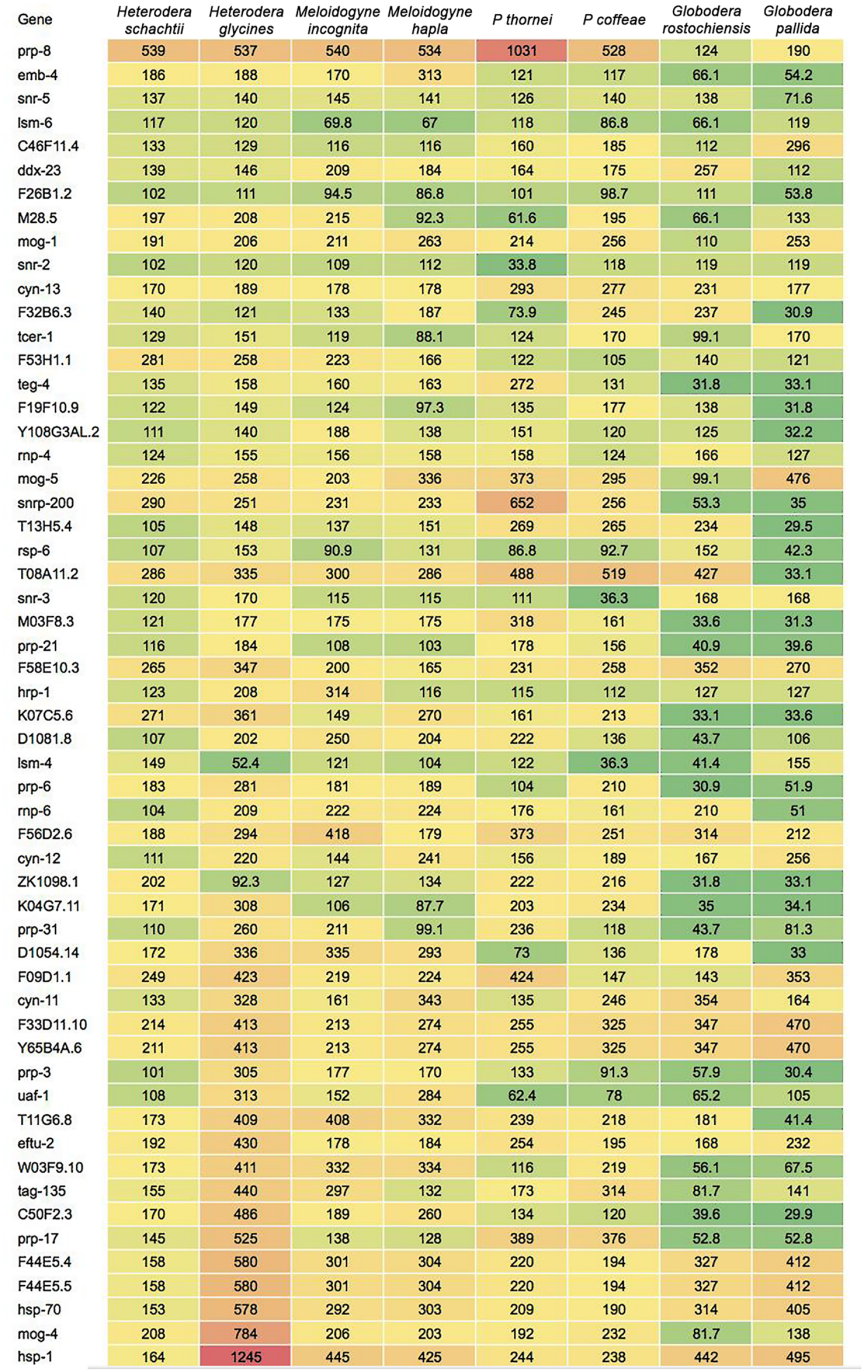
*C*. *elegans* spliceosomal genes showing similarity to *H*. *schachtii* transcripts and their similarity scores for genes of seven other PPNs.

Very little is known about the components/genes of the spliceosome and the mechanism of trans-splicing in PPNs. We used the 104 genes involved in the KEGG spliceosome pathway of *C*. *elegans* to identify orthologues in the *H*. *schachtii* transcriptome and compared these to those of the PPNs *H*. *glycines*, *M*. *incognita*, *M*. *hapla*, *P*. *thornei*, *P*. *coffeae*, *G*. *rostochiensis and G*. *pallida*. The results are presented as a heat map with colours indicating relative values of the bit score from the alignments (Fig 6). Using a threshold bit score of 100, twenty of the 104 genes were present in all the PPNs with *prp-8* (pre-mRNA-processing factor 8), the most conserved. In all, 56 homologues were identified in the *H*. *schachtii* transcriptome compared to 81 in ESTs of *H*. *glycines*.

Global analysis shows that over 70% of *C*. *elegans* expressed genes are trans-spliced to one of two 22 nucleotide splice leaders [35]. Using the regular expression derived from the conserved regions of SL1 RNA genes of *C*. *elegans* and *P*. *pacificus*, which usually has the canonical 22 nt SL1 leader sequence (GGTTTAATTACCCAAGTTTGAG) and a Sm binding site (ATTTTGGAAC)[32], we searched for existence of a similar SL1 RNA gene in the genomic sequences of *H*. *glycines* using BLASTN and manual curation. Of the 45,526 genomic contigs of *H*. *glycines* deposited at NCBI, 218 had at least one variant (with 0–3 mismatches) of the SL1 sequence above: for 182 of these the SL1 was a 100% match to the query, 23 others had the maximum of three mismatches and 13 with one or two mismatches. Genomic contig ABLA01003722.1 contained the maximum number of five SL1s, approximately 600 bases apart at positions 541, 1161, 1770, 2380 and 2990. Of the sequences with SL1, 152 also had a sequence similar to the Sm binding site. Using this information, we identified 823 *H*. *schachtii* reads with variants of the SL1 sequence. There were 587 reads with 100% match to the SL1 sequence. The reads ranged from 42 to 561 nt long with only 75 of them <100 nt long. About 260 of the transcripts were annotated (e-value of 1E-05). Of these, 71 putatively encoded ribosomal proteins and subunits including *rps-7*, *rps-15*, *rps-23* and *rpl-19* homologues of *C*. *elegans*, *G*. *rostochiensis*, *G*. *pallida*, *B*. *xylophilus*, *B*. *malayi* and *L*. *loa*. Others were similar to transcripts for vacuolar H ATPase protein 16 (*vha-16)*, 1, 4-beta endoglucanase, splicing factors (e.g. *rsp-6*), FMRFamide-like protein 5 and 16 (*flp-5*, *flp-16*), expansin B1, troponin C (*pat-10*), synaptobrevin (*snb-1)*, mucin-19-like protein, heat shock factor-binding protein 1-like and cold-shock-like protein (*csp-1)*. None of the SL2-like sequences found in the *Rhabditina* familiy was associated with any transcript.

### Transcripts putatively encoding carbohydrate active enzymes

Using BLASTX to search the 188,623 protein sequences of the CAZy database, a total of 407 *H*. *schachtii* transcripts (226 contigs and 182 singletons) matched sequences of Glycoside Hydrolases (GHs), Carbohydrate Binding Modules (CBMs), Polysaccharide Lyases (PLs), Carbohydrate Esterases (37), Glycosyl Transferases (GTs) and proteins of Auxiliary Activities (AA) of other organisms. Of these, 375 had a maximum HSP score of at least 50 to the best matching CAZyme (S3 Table). The matching CAZymes were from the following CAZy families: eight CBMs, five AAs, three CEs, 39 GHs, two PLs and 31 GTs and were mostly sequences of other nematodes particularly *Caenorhabditis* spp., *A*. *suum*, *Strongyloides ratti* and plant parasitic nematodes.

The number of transcripts (36) matching to CAZymes of plant parasitic nematodes is shown in Table 1. Five contigs and four singletons matched PL3 pectate lyases of *B*. *xylophilus* (AGI04333.1), *Globodera tabacum tabacum* (AEA08812.1), *H*. *glycines* (AAM74954.1, ADW77535.1, ADW77534.1), and a pectate lyase precursor of *H*. *schachtii* (ABN14273.1, ABN14272.1). Fourteen contigs and five singletons putatively encode GH5 CAZymes as these transcripts had high percentage similarities to those of enzymes identified for other plant parasitic nematodes of six genera including those characterised for *H*. *schachtii* and the closely related *H*. *glycines* (Table 1). Twenty five transcripts matched GH CAZymes with multiple domains and those from PPNs were usually GH5s with CBM2 family (Table 1 and S3 Table). Together with pectate lyases which act on pectin components of plant cell walls, GH5 cellulases or beta 1,4 endoglucanases, secreted from gland cells, are generally characterised in most PPNs and are thought to modify cell walls, mainly facilitating intracellular migration of nematode juveniles through host tissues but possibly also involved in syncytial expansion [7]. In addition, two contigs matched a GH53 arabinogalactan endo-1,4-beta-galactosidase 1 of *H*. *schachtii*, an enzyme capable of hydrolysing beta-1,4-galactan: such a protein could play a role in modifying plant cell wall during migration of the nematode through roots [36].

**Table 1:** *H*. *schachtii* transcripts putatively encoding CAZymes similar to those of plant parasitic nematodes.

CAZyme family	Accession number of best matching CAZyme	Maximum HSP scores to best matching *H*. *schachtii* transcripts	Number of transcripts matching CAZyme	Description of CAZyme	Organism of CAZyme origin
GT20	CAH18870	110.15	4	Putative trehalose 6-phosphate synthase	*Aphelenchus avenae*
GH5	CAC12958.1	383.64	2	Beta-1,4-endoglucanase 1 precursor, partial	*Heterodera schachtii*
	AFQ55682.1	109	1	Beta-1,4-endoglucanase 3	*Heterodera avenae*
	AAM50039.1	114.78	3	Putative gland protein G26D05	*Heterodera glycines*
	AER27792.1	133.65	1	Beta-1,4-endoglucanase, partial	*Pratylenchus vulnus*
	AAC48326.1	135.96	1	Beta-1,4-endoglucanase-2 precursor	*Heterodera glycines*
	AAK85303.1	62.77	1	Beta-1,4-endoglucanase-4	*Heterodera glycines*
GH5, CBM2	AAC15707.1	193.74	2	Beta-1,4-endoglucanase-1 precursor	*Heterodera glycines*
	AAC63988.1	65.08	1	Beta-1,4-endoglucanase precursor	*Globodera rostochiensis*
	ABV54447.1	80.49	1	GHF5 endo-1,4-beta-glucanase precursor	*Radopholus similis*
	ACO55952.1	88.2	1	Beta-1,4-endoglucanase	*Heterodera avenae*
	CAC12959.1	194.9	2	Beta-1,4-endoglucanase 2 precursor	*Heterodera schachtii*
	AAN32884.1	218.78	2	Cellulase ENG-5	*Heterodera glycines*
	CAC12958.1	383.64	2	Beta-1,4-endoglucanase 1 precursor, partial	*Heterodera schachtii*
GH53	ACY02855.1	96.67	3	Arabinogalactan endo-1,4-beta-galactosidase 1	*Heterodera schachtii*
PL3	ABN14272.1	203.76	3	Pectate lyase precursor	*Heterodera schachtii*
	AGI04333.1	49.68	2	Pectate lyase 3	*Bursaphelenchus xylophilus*
	AEA08812.1	60.08	1	Pectate lyase 1, partial	*Globodera tabacum tabacum*
	AAM74954.1	70.09	1	Pectate lyase 2	*Heterodera glycines*
	ADW77534.1	171.78	2	Pectate lyase	*Heterodera glycines*

A majority (21) of the best CAZyme matches from the 31 GT families were to sequences of *Caenorhabditis* spp. and *A*. *suum* and were mostly enzymes involved in common molecular processes of development, for example N-acetyllactosamine synthase, glycogen phosphorylase and chitin synthase (S3 Table). The only CAZyme of a plant parasitic nematode origin with a GT activity was putative trehalose 6-phosphate synthase of *Aphelenchus avenae*; this enzyme is involved in metabolism of trehalose which is important in several developmental processes including energy reservation, egg hatching and protection from biotic stresses [37]. The best CAZyme matches to the *H*. *schachtii* transcripts for some families were of non-nematode origins. These were sequences of plant, fungi, bacteria and insect species (S3 Table).

To confirm that these were not contaminants in the transcriptome, the matching CAZymes were compared to nematode sequences in the NCBI databases using BLAST. All but eight of the 40 CAZymes matched significantly to sequences of various nematode species including *Caenorhabditis spp*, *S*. *ratti*, *Trichinella* spp and plant parasitic nematodes. The presence of transcripts matching the eight CAZymes with no significant match to any other nematode sequence in the NCBI database needs further investigation.

### *H*. *schachtii* orthologues of putative nematode parasitism genes

Plant parasitic nematodes successfully parasitise their hosts by employing secretions from pharyngeal gland cells, amphids and the hypodermis: the secreted peptides, which are responsible for various activities, are generally described as ‘effectors’. These are thought to play a number of roles which include suppression of host defences, enabling migration in plant tissues, promotion of nematode feeding, formation of feeding tubes, digestion of ingested cytoplasm, and host cell modification leading to the induction and maintenance of feeding structures such as syncytia for cyst nematodes or giant cells for root knot nematodes. To assess whether the pre-infective J2s of *H*. *schachtii* are primed for infection, we compared the transcriptome to sequences of 30 genes (29 complete cDNAs and one partial cDNA) representative of the effector repertoire of plant parasitic nematodes: for each of these there is evidence of secretion from gland cells and/or an effector function during interaction with host plants.

In all, 148 contigs and 183 singletons were identified with sequence similarity to 25 of the effectors (Table 2). These included proteins that can modify host metabolic profiles (e.g. chorismate mutase), and those that may aid digestion of host cell contents (cathepsin L) or are potentially secreted to protect the nematode against host defence (e.g. peroxiredoxin). Also there were transcripts with similarity to MAP-1 gene of *Meloidogyne* species, which is potentially involved in the early stages of host recognition [38]. Transcripts of high sequence identity to the *Hg30C02* effector of *H*. *glycines*, expression of which increases susceptibility of Arabidopsis to infection by *H*. *schachtii* and the *Hs19C07* effector, which interacts with the Arabidopsis auxin influx transporter LAX3 to facilitate syncytium development, were identified [5, 39, 40]. There were two transcripts with low HSP scores to the *10A06* effector of *H*. *schachtii*: expression of this gene induces morphological changes in the host, targets spermidine synthase and possibly disrupts Arabidopsis defense signalling leading to increased susceptibility to infection [41].

**Table 2:** *H*. *schachtii* transcripts similar to those encoding parasitism effectors of plant parasitic nematodes.

Nematode (putative) parasitism gene	Reference Nucleotide sequence	Number of matching contigs, singletons	Length of best matching transcript (nucleotides)	Total alignment score	E-value	Query coverage (%)	Reference for evidence of effector activity
Galectin (*G*. *rostochiensis*)	AF002989.1	1,3	184	363	1.35E-14	93	[43]
SEC-2 protein (*G*. *pallida*)	Y09293.2	1,30	448	929	8.56E-58	98	[44]
Putative hypodermis secreted protein (sxp1)(*G*. *rostochiensis*)	AJ271910.1	3,1	279	536	2.15E-30	79	[45]
Secreted venom allergen-like protein VAP2 (*H*. *glycines*)	AY028639.1	1,4	379	1315	2.69E = 59	85	[46]
Secreted glutathione peroxidase (gpx1) (*G*. *rostochiensis*)	AJ493677.1	3,2	474	1005	8.33E-53	93	[47]
Annexin 4C10 (*H*. *glycines*)	AF469059.1	6,3	552	1769	3.13E-62	92	[48]
10A06 effector (*H*. *schachtii*)	GQ373256.1	1,1	454	192	6.84E-08	40	[41]
Gland protein G30C02 (*H*. *glycines*)	AF502393.1	2,0	284	1016	1.38E-35	88	[40]
Ubiquitin extension protein (Ubi1) (*H*. *schachtii*)	AY286305.1	9,3	967	1661	5.37E-75	45	[48]
Expansin (EXPB1) (*G*. *rostochiensis*)	AJ311901.1	1,3	189	300	2.79E-14	100	[49]
Chorismate mutase (cm-1) (*H*. *glycines*)	AY160225.2	3,3	320	1576	2.26E-68	100	[50]
Gland protein G19C07 (*H*. *glycines*)	AF490250.2	4,2	253	621	8.27E-29	95	[39]
RBP-1 protein (Rbp-1-Al100) *(G*. *pallida)*	JF933885.1	2,1	433	199	1.19E-08	48	[51]
Secreted SPRY protein 19 (*G*. *rostochiensis*)	JX026920.1	8,8	499	408	1.62E-14	55	[52]
Venom allergen-like protein (VAP1) (*G*. *rostochiensis*)	KF963519.1	4,1	420	1009	7.49E-50	71	[46]
Putative amphid protein (Ams1) (*G*. *rostochiensis*)	KF963524.1	2,2	394	414	6.01E-24	77	[53]
Peroxiredoxin (Tpx) (*G*. *rostochiensis*)	KF963527.1	2,5	485	865	6.04E-69	88	[54]
Putative cathepsin L protease (cpl-1) (*M*. *incognita*)	AJ557572.1	9,22	504	496	1.01E-48	67	[55]
MAP-1 protein (*M*. *incognita*)	AJ278663.1	48,65	229	188	9.06E-09	79	[38]
Glutathione S-transferase-1 (gsts-1) (*M*. *incognita*)	EF429119.1	5,2	411	391	2.03E-34	100	[43]
Nuclei-targetted RKN-secreted protein (*M*. *incognita*)	JK307566.1	0,2	229	188	7.33E-07	79	[56]
14-3-3 protein (*M*. *incognita*)	AF402309.1	18,5	431	751	1.05E-88	100	[57]
Calreticulin (*M*. *incognita*)	AF402771.1	4,3	628	1003	7.93E-104	99	[58]
Polygalacturonase (*M*. *incognita*)	AY098646.1	1,0	283	149	1.09E-05	65	[59]
Transthyretin-like protein 1 (ttl-1) (*R*. *similis*)	AM691117.1	10,12	736	926	3.97E-36	47	[60]
Beta-1,3-endoglucanase (*B*. *xylophilus*)	AB194803	0,0	No true homologue				[61]
GHF45 family protein (*B*. *xylophilus*)	JQ314425	0,0	No true homologue				[62]
Chitinase (*H*. *glycines*)	AF468679	0,0	No true homologue				[42]
Xylanase (xyl-1) (*M*. *incognita*)	AF224342	0,0	No true homologue				[63]
Cellulose binding protein (cbp-1) (*M*. *javanica*)	AM491771	0,0	No true homologue				[64]

In addition to transcripts putatively encoding the cell wall-modifying CAZymes, beta 1, 4 endoglucanase, pectate lyase and arabinogalactan endo-1, 4-beta-galactosidase 1, *H*. *schachtii* transcripts with relatively low sequence similarity to two other cell wall-modifying enzymes were identified from the transcriptome; one transcript for polygalacturonase (*M*. *incognita*) and four transcripts for expansin (*G*. *rostochiensis*) (Table 1). However, no transcripts were found with sequence similarity to those of cell wall-modifying enzymes beta-1,3-endoglucanase (*B*. *xylophilus*), GHF45 family protein (*B*. *xylophilus*) and xylanase (xyl-1) (*M*. *incognita*), and none matched gene sequences of cellulose binding protein precursor (cbp-1) of *M*. *javanica* and a chitinase (*H*. *glycines*). The latter has not been characterised in detail, but accumulates specifically in the subventral oesophageal gland cells of parasitic stages of *H*. *glycines*, although not in eggs or hatched pre-parasitic second-stage juveniles (Table 2) [42].

Effectors secreted by the hypodermis and amphids of plant parasitic nematodes have not been well-studied. In this study, we identified transcripts that potentially encode similar putative effectors secreted from the amphid (e.g. Ams1) and hypodermis (e.g. sxp1) of *G*. *rostochiensis* (Table 2). Recently, amphid-secreted effectors of *G*. *pallida*, HYP effectors, that have hyper-variable regions in individuals of the nematode population have been characterised [65]. No *H*. *schachtii* transcript with significant identity to sequences of these effectors was identified.

Various EST libraries of gland cells and contents have been constructed for the cyst nematode *H*. *glycines* and the root knot nematode *M*. *incognita* and are available in the NCBI databases [9, 66–69]. Whilst a small number of these transcripts have been studied in detail, a majority remain uncharacterised. A total of 11,561 *H*. *schachtii* transcripts matched 4,995 of the 15,226 gland cell-derived ESTs of *H*. *glycines* mostly at e-values much lower than the 1E-05 threshold and with very high HSP scores, the highest at 597 with an average of 93. Of the 40 putative oesophageal gland cell secretory proteins (msp1-40) for *M*. *incognita* [67], six (msp10, 21, 26, 28, 29, 34 and 40) had matches to a total of 35 *H*. *schachtii* transcripts with e-values between 1E-05 and 1E-14 and maximum HSP scores between 31 and 69. Generally, matching *H*. *schachtii* transcripts had high identity to (putative) effectors of cyst nematodes compared to those of root knot nematodes. When SignalP4.1 was used to predict the presence and location of signal peptide cleavage sites putatively encoded by all transcripts with matches to those of nematode effectors and CAZymes, four fully translated transcripts putatively encoding >50 amino acids were identified (S4 Table). These transcripts were most similar to those of three effectors of *H*. *glycines* (annexin 4C10, chorismate mutase, the gland protein G19C07) and calreticulin of *M*. *incognita*. None of the four transcripts was predicted to have a transmembrane helix; this feature and the presence of a signal peptide are two important features of nematode effectors that are secreted and transported into host cells.

### RNAi genes identified in the *H*. *schachtii* transcriptome

RNAi is a natural gene regulation mechanism in eukaryotes. Genes involved in RNAi pathways in *C*. *elegans* are well-characterised and have been used as a model to understand the process in other organisms. It is known that different numbers of genes are involved in the various processes of this otherwise conserved process in different organisms [70]. Moreover, in PPNs there seem to be differences in susceptibility to RNAi between cyst and root knot nematodes, and also within *Pratylenchus* species [24]. The recent sequencing of genomes of *M*. *incognita*, *M*. *hapla* and *G*. *pallida* provides information on RNAi genes in these organisms. However, there is currently no information on similar genes for *H*. *schachtii*. To identify such genes from the transcriptome, we compared the transcripts to protein sequences of 97 functionally characterised RNAi effectors of *C*. *elegans* (S1 Text) including 27 argonautes and to those identified in published transcriptomes of *P*. *coffeae*, *H*. *avenae* and the genomes of *M*. *incognita*, *M*. *hapla* and *G*. *pallida*.

There were significant matches to 36 of the 97 RNAi effectors characterised for *C*. *elegans* (Table 3). Interestingly, at least one of the genes was involved in all the major functional classes of the RNAi machinery i.e. spreading of RNAi triggers into and out of cells (e.g. *xpo-1*), the dicer complex (e.g. *dcr-1*, *drh-1*), the RNAi-Inducing Silencing Complex (e.g. *ain-1*), RNAi amplification (e.g. *ego-1*), RNAi inhibitors (e.g. *eri-1*) and nuclear RNAi effectors (*cid-1*). In addition, transcripts similar to two prominent genes involved in the processing of primary microRNA transcripts, *drsh-1* and *pash-1*, and for argonautes including worm-specific argonautes (WAGOs) were also identified in the transcriptome. More genes putatively encoding RNAi effectors were identified in this study than the number published for transcriptomes of *P*. *coffeae* and *H*. *avenae* and genomes of *M*. *incognita* and *G*. *pallida* [15, 17, 19, 71](Table 3). A possible explanation for this difference is the larger pool of *C*. *elegans* RNAi effectors used for this study, which included those recently characterised. Transcripts for only seven of the RNAi effectors were identified in all five nematodes. With the exception of the *P*. *coffeae* transcriptome, putative RNAi effectors for each of the functional classes of RNAi had been identified (Table 3). This information provides a template for comprehensive study of RNAi in cyst nematodes and how the phenomenon can be applied as a potential strategy to control PPNs.

**Table 3:** RNAi genes identified in the *H*. *schachtii* compared to those identified from published genomes and *M*. *incognita* and *G*. *pallida*, and transcriptomes of *H*. *avenae* and *P*. *coffeae*.

Functional classes of RNAi effectors	*H*. *schachtii*	*M*. *incognita* genome [15]	*G*. *pallida* genome [17]	*H*. *avenae* Transcriptome [71]	*P*. *coffeae* transcriptome [19]
RNA transport	*haf-6*, *rsd-3*, *sid-3*, *xpo-1*	*rsd-3*, *xpo-1*	*rsd-3*	*rsd-3*, *xpo-1*	*-*
Dicer complex	*dcr-1*, *drh-1*, *drh-3*, *drsh-1*, *pash-1*	*dcr-1*, *drh-1*, *drh-3*, *drsh-1*, *pash-1*	*dcr-1*, *drh-3*, *drsh-1*, *pash-1*	*dcr-1*, *drh-1*, *drh-3*, *drsh-1*	*dcr-1*, *drh-1*, *drsh-1*
RISC	*ain-1*, *tsn-1*	*ain-1*, *tsn-1*	*tsn-1*	*ain-1*, *tsn-1*	*tsn-1*
RNAi amplification	*smg-2*, *ego-1*, *rrf-1*	*smg-2*, *ego-1*, *rrf-1*	*smg-2*, *ego-1*,	*smg-2*, *ego-1*, *rrf-1*	*smg-2*, *ego-1*
RNAi inhibitors	*eri-1*, *eri-7*, *rrf-3*, *xrn-2*, *zfp-2*	*eri-1*, *xrn-2*, *rrf-3*	*eri-1*, *xrn-2*	*eri-1*, *eri-7*, *xrn-2*	*eri-1*
Nuclear RNAi effectors	*cid-1*, *ekl-1*, *ekl-4*, *mes-2*, *mes-6*, *mut-2*, *rha-1*, *zfp-1*	*cid-1*, *ekl-1*, *ekl-4*, *mes-2*, *rha-1*	*cid-1*, *ekl-4*, *mes-2*, *rha-1*	*cid-1*, *ekl-1*, *mes-2*, *mut-2*, *rha-1*, *zfp1*	*-*
Argonautes	*alg-1*, *alg-2*, *sago-1*, *ppw-2*, *hrde-1*, *wago-4*, *wago-1*, *wago-11*, *wago-11*	*alg-1*, *alg-2*, *wago-4*, *wago-1*,	*alg-1*, *hrde-1*, *wago-4*, *wago-1*, *wago-11*, *wago-11*	*alg-1*, *sago-1*, *ppw-2*	*alg-1*, *alg-2*, *ppw-2*

Note: Genes underlined have been identified in published transcriptomes and genomes of the five nematodes.

### *H*. *schachtii* may harbour virus-like genomes

**Fig 7:**
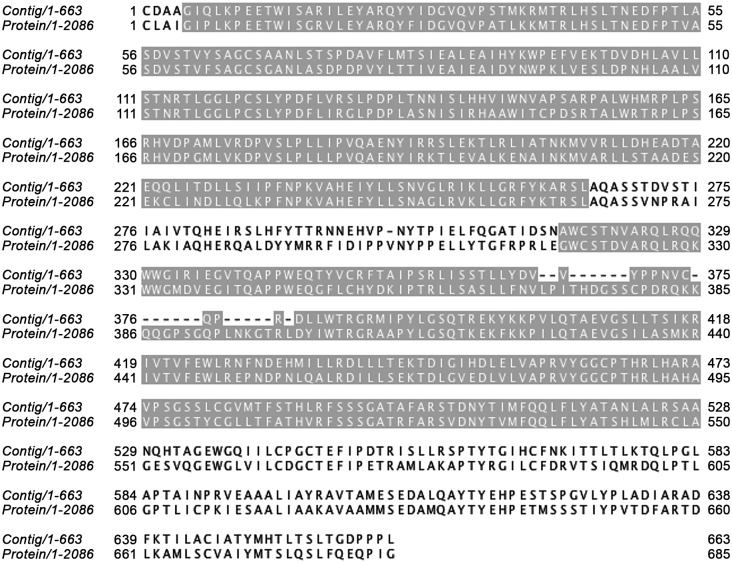
Alignment of a *H*. *schachtii* contig and virus protein showing two domains with significant similarity.

A recent analysis showed that the transcriptome of *H*. *glycines* was associated with virus-like genomes, and genome fragments of four different RNA viruses were found in the transcriptome, including the Soybean cyst nematode nyavirus (ScNV) [23]. Using TBLASTX, 278 of the transcriptome reads were similar to the full-length sequence of the Soybean cyst nematode nyavirus (ScNV, PRJNA258186), the best was 461 nt long (e-value 7.24E-80, bit score 1,366). This similarity was confirmed using read mapping where 248 reads were mapped to the genome. Thirty nine contigs of the *H*. *schachtii* J2 transcriptome had high identities to the ScNV genome, the best contig with e-value of 2E-175, total bit score of 1,373 and maximum HSP score of 534. The contig with the best alignment matched the putative RNA dependent RNA polymerase protein (AEF56729.1). This contained an open reading frame of 663 amino acids and matched the viral protein with a maximum score of 885 and with 63% protein identity. Also, two contigs matched the glycoprotein (AEF56728.1) with an e value of 9E-114 and a bit score of 372. Transcripts with identity to two conserved domains characteristic of RNA dependent RNA polymerase of members of the Mononegavirales, matched the phosphoprotein, P (grey residues in Fig 7), which acts as a transcription factor and the large protein L, which confers the RNA polymerase activity on the complex and carries a motif, GxxTx(n)HR, that is essential for mRNA cap formation. Notably, no *H*. *schachtii* read or contig matched the ORF2 and ORF3 that encode the hypothetical proteins of the ScNV.

## Discussion

The J2 stage of a cyst nematode is often viewed as the most important stage in its life cycle because this life stage must be able to move through soil to locate a host root, enter and migrate within it while evading or suppressing the host immune responses, to a site where it initiates the formation of a syncytium. The J2 stage can be divided into pre- and post-plant entry stages. In addition to expression of genes involved in general metabolism, the results presented here clearly show that even before root entry the pre-parasitic J2 is already primed to express many parasitism-related genes of varied functions needed for it to reach host roots, enter them and migrate through them, evade host defences, and initiate syncytium development. Post-root entry J2s may well express additional parasitism genes, and there will undoubtedly be changes in the levels of many transcripts as the nematodes become sedentary, expand and reproduce as has been found for *G*. *pallida* [72]. A more detailed stage-specific set of transcriptome data is needed to follow changes in gene expression for post-entry J2s and subsequent developmental stages. However, the data generated here are sufficient to identify sequences of potential target genes that can be used to develop new methods to control pre-parasitic J2s, and so reduce or prevent root infestation.

The analysis of the pre-parasitic J2 transcriptome of *H*. *schachtii* has thus enabled us to identify genes expressed at this stage and with possible roles in host parasitism. Many of the transcripts were similar to genes found in other endoparasitic nematodes, whilst some were exclusive to cyst nematodes. Not surprisingly more of the transcripts share sequence homology to those of cyst nematodes with about 67% of the reads mapping to genomic sequences of the closely related *H*. *glycines*. Transcripts similar to those of 2,410 *C*. *elegans* proteins were identified including genes involved in splicing and trans-splicing and those with direct roles in RNA interference. Some of those transcripts with no homology to genes of free-living nematodes were identified as common to PPNs and may well be required for parasitism in general or specifically by cyst nematodes for plant parasitism.

Once inside the root J2s of *H*. *schachtii* typically migrate intracellularly through cells to a site at which they establish a metabolically active syncytium [10, 12]. They need to secrete enzymes that can modify key polysaccharide components of host cell walls (cellulose, hemicelluloses and pectin) to facilitate these activities. The expression before root entry of a range of such transcripts encoding carbohydrate active enzymes clearly shows that pre-parasitic J2s of *H*. *schachtii* are primed for host infection. Examples include transcripts of the beta-1,4- endoglucanase and its precursors, a cellulase with GH5 enzyme activity which catalyses the hydrolysis of the glycosidic bonds in cellulose. Similar transcripts for cellulases found here had high percentage sequence identity to those previously characterised for *H*. *schachtii*, and two other cyst nematodes, *G*. *tabacum solanacearum* and *H*. *glycines*. Some were predicted to have signal peptides and carbohydrate active binding modules, both of which are characteristics of cellulases widely deployed by PPNs including migratory endoparasites such as *P*. *thornei* [20]. The identification of transcripts of pectate lyase, which is involved in modification of pectic polymers, is consistent with the expression and deployment of this gene product by other cyst nematodes during infection [13].

Consistent with other studies, for most effectors of root knot nematodes there was either no similar transcript in the *H*. *schachtii* transcriptome or matching transcripts had very low similarity scores. These genes may be absent from the genome of *H*. *schachtii* or they may be present but not expressed during early parasitism. To validate this observation, four characterised effectors of *M*. *incognita*, xylanase (MiXly, AF224342), NodL factor (AW829666.1), polygalacturonase (AY098646.1), and 16D10 (Q6YKB1.1) were used as references to identify similar transcripts from the genomic sequences of the cyst nematodes *H*. *glycines* and *G*. *pallida* using BLASTX at an e-value cut-off 1E-03. As with the *H*. *schachtii* transcripts, no genomic contig of either cyst nematode genome appears to encode any protein similar to Mixyl and Mi16D10. For NodL, whilst there was no match to any *H*. *schachtii* transcript, five genomic contigs of *H*. *glycines* (the best with a maximum bit score of 131) and one for *G*. *pallida* with low maximum bit score (34) were identified. There was only one contig each from the transcriptome of *H*. *schachtii* (at e-value 1.09E-5) and genome of *G*. *pallida* (at e-value 9.33E-5), but none from *H*. *glycines* that appears to encode a polygalacturonase similar to that of root knot nematodes. Differences in the complements of effectors, particularly cell wall modifying enzymes, probably reflect the different modes of migration through host roots: J2 root knot nematodes migrate intercellularly through cells walls rather than intracellularly from cell to cell, and so require a different set of enzymes to modify cell walls: fewer such enzymes would be needed for intracellular migration by J2 cyst nematodes [21, 73].

In addition to intracellular migration in roots, endoparasitic J2 and subsequent stages must be able to evade or modulate the host immune system. Some effectors involved in these activities may be secreted via the stylet, but others thought to be involved in this process may be synthesised and secreted directly via the amphids, or via the hypodermis after being transported across the cuticle, which is in direct contact with the external environment. Such effectors include the MAP-1 in J2 amphidial secretions of *M*. *incognita* possibly involved in the early stages of recognition between resistant plants and avirulent nematodes [38], and enzymes known to regulate plant defense responses or signalling such as peroxiredoxin (Gr-tpx), glutathione peroxidase (Gr-gpx1), a glutathione S-Transferase (Mi-gst-1) which counter reactive oxygen species mediated signalling, and a fatty acid and retinol binding protein (FAR-1, *G*. *pallida*, *M*. *incognita*) present on cuticular surfaces of nematodes. The latter appear to bind lipid precursors of plant defence compounds and prevent their metabolism [44, 74]. Transcripts with significant similarity to two putative effectors of *G*. *rostochiensis*, Gr-ams1 and Gr-sxp-1, secreted from the amphids and hypodermis respectively, were also identified. Another class of hyper-variable apoplastic effector gene family termed HYP has been characterised in *G*. *pallida* and is apparently only present in cyst nematodes and *Rotylenchulus reniformis*. HYP effectors are secreted from the amphids, and are required for successful infection. Interestingly, full length transcripts identical to these effectors were not found in the pre-J2 *H*. *schachtii* transcriptome [65]. The identification of transcripts encoding similar effectors including the SXP-RAL2 gene family, secreted from the epidermis and the amphidial sheath cells in *G*. *rostochiensis* [45] indicate these effectors are synthesised in the pre-parasitic J2, pre-entry and not necessarily synthesised in response to plant defence signalling.

Two well-characterised secreted proteins of cyst nematodes are known to be functionally similar to and can mimic the functions of host plant orthologues [50, 75]. These are the CLE-like proteins and chorismate mutase: transcripts of both were present in the pre-parasitic J2 transcriptome. Chorismate mutases of plant parasites (e.g. nematodes and fungi) change the metabolic status of host cells through metabolic priming or alter the synthesis of chorismate-derived products, resulting in a down-regulation of a host plant defense [50, 76–78]. Similar transcripts for the two proteins and for those of several secreted proteins have been shown to express in the single dorsal and/or the two subventral oesophageal cells of pre-parasitic and parasitic stages of *H*. *glycines* and *G*. *rostochiensis* [9, 78, 79]. Complete sequences of orthologues of most of these genes have not been found in the transcriptomes of migratory endoparasites [19–21]. It is possible such genes are required specifically by cyst nematodes to initiate or maintain feeding sites by modulating plant growth or metabolic processes at feeding sites, or there may be orthologues present in migratory nematodes that have yet to be characterised.

There are reports that both free-living (*C*. *elegans* and *C*. *briggsae*) and some PPNs (e.g. *H*. *glycines*) can harbour viruses [23, 80]. In the transcriptome of J2 *H*. *schachtii* reads mapped to the genome sequence of ScNV and there was a significant match of contigs to three of the five open reading frames of this virus: this provides good evidence that a virus similar to ScNV is also present in *H*. *schachtii*. New sequencing technologies have revealed the presence of many new viruses in animals and plants, and have given rise to a view that not all viruses are pathogens, some are vertically transmitted, and some can be mutualistic or beneficial [81]. The source of viruses in PPN genomes is not known at present; possible sources are the plants from which they feed or from the soil environment. J2s of sedentary nematodes, which are obligate parasites, usually find a host to feed from soon after hatching. In the case of the *H*. *schachtii* analysed here, because the J2s had not fed from host plants, the source of infection is either from soil or was acquired through vertical transmission from the preceding generation. Functional characterisation of these sequences and other transcripts identical to uncharacterised gene products could provide additional insight into the parasitic life of *H*. *schachtii* and potentially to other parasitic nematodes. Some of the transcripts may be involved in development and metabolism of the nematode, but others could be involved in nematode-host interactions.

In this work we have demonstrated that the pre-parasitic J2 stage of *H*. *schachtii* is primed for host root entry, and expresses many of the transcripts whose products will be employed in root entry, migration through host tissues, evasion or modification of host defences, and also some of the effectors thought to be required for induction of syncytial feeding cells. The identification of a substantial set of candidate parasitism genes also provides some new targets for their control. Evidence is also provided for the presence of viral sequences in J2s of *H*. *schachtii*: this aspect and the presence of many other unannotated transcripts require further study, for example using RNAi, which has been used to characterise some effectors found in this study.

## Supporting Information

S1 Fig A mist apparatus for extracting nematodes from soil. (TIF)

S1 Table Sequences and databases used for annotation of the *H*. *schachtii* transcriptome. (XLSX)

S2 Table PAMGO terms associated with *H*. *schachtii* transcripts. (XLSX)

S3 Table *H*. *schachtii* transcripts putatively encoding carbohydrate active enzymes. (XLSX)

S4 Table *H*. *schachtii* transcripts predicted to encode signal peptides without trans-membrane helices. (XLSX)

S1 Text Translated sequences of RNAi genes of *C*. *elegans* used for identifying similar transcripts of *H*. *schachtii*. (DOCX)
