# Supplementary figures and images for: Analysis of the Transcriptome of the Infective Stage of the Beet Cyst Nematode, H. schachtii
Source: PLoS One. 2016 Jan 29;11(1):e0147511. doi: 10.1371/journal.pone.0147511 (PMC4733053; doi:10.1371/journal.pone.0147511)

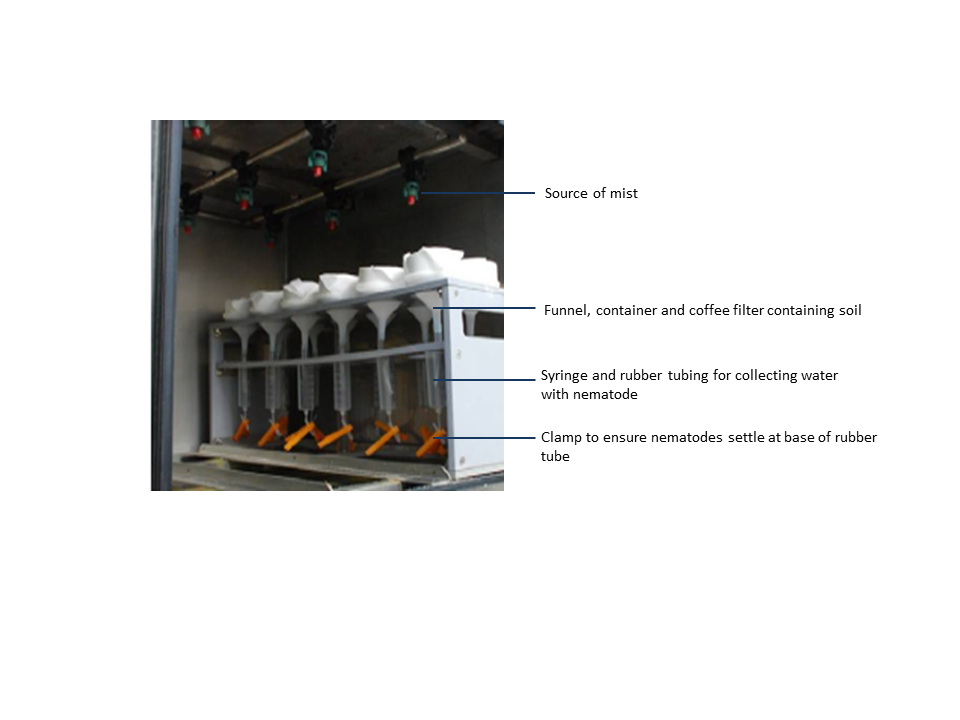

Supplement: S1 Fig — (TIF) [file pone.0147511.s001.tif]
