# Supplementary material for: Analysis of the Transcriptome of the Infective Stage of the Beet Cyst Nematode, H. schachtii
Source: PLoS One. 2016 Jan 29;11(1):e0147511. doi: 10.1371/journal.pone.0147511 (PMC4733053; doi:10.1371/journal.pone.0147511)
Supplement: S1 Text — (DOCX) [file pone.0147511.s006.docx]

Protein sequences of RNAi genes of *C.elegans* used to identify *H. schachtii* transcripts putatively encoding similar proteins

>ADR-1

MDQNPNYNFGYGQAYGSGTDHTSDSTNNYNWASQWSQPESAASLATHTFPQYVSQQQQQQQQQAQQQAQNTYAAMNPISTFMQQQQRAQTFPQKKYGQQGGAPKPSAIRNNNFGAFGGGHALSQEWVQPMSQNQMGGPQGNRFFNNQKGGPFNQNKPNWRQNKPKGPAAPKKFDSTGKSPAMLLHELFKDVSEEYTEVEGVPKKYCCTLKVNGRTFQMESVNKKAAKQKCSELVVRDLRPDVHVTPFEEGVAAKAAAPVKKEIDAASGNGQNNKRNLLQADAISNQPTPKKVSAVKKAKLQLTPVESALSLLDLMQKIIAESAEKYSPVFEASEVPKDPEIPEVEVKKEEVDTNGENVANEKKSGWRKNETMHNVTLKFVEQNKQYTKMGPSRGVLKDMVIREALRDLFNVSHADITTVARRHASNRLGHDTTILQCLNTICSILNCTLTIECEPAEDRPLGIGRAYFMAKCTIIDHNENDLKFEVKSSSLASKAMAKDWVAQETLKNYFAIDPSSCVKTDAVSSQGPCALLHAMLNKQTKQKCKIAYEFKDNVPPVAGQATTTFYCECVIDETDRYIGVGRSKKLAKSEAAMQALKKLFKIDYDPAGNYPLALTSRAMTESKVSPLCRHIAEFCKREYHQMTEYYQIPPSNLFAAFLLVNAQEEKRVLAMGSSIQYIVEPDTLSGANGTSLLHLDAIILARRAMLKAFIHELSTVDSECSIFEKKEEGKAALKPNLRLVLYSNYSPPCIHAVDDAATKKLSYVTPTNLTCVPDDVLTYEQIKETKSLRVHCTADKLFKWNTLGIQGALLSNVLHPIFIDNIFFGSEAPVSDESLSYALQGRLGPNENEREIIVESMPVQMRMHMGISHLWHRGVDSVETLDYNTGRTSKGSPSRVCKAEIFEAYRKLNGVDQAVVNYAKAKEMASEYQYEKKVFYEKLEAAGLGKWQTKPAELVDSFTLAAFD

>ADR-2

MSVEEGMEVELKSEKMDLDDNIPDFVKETVERSGKNPMSLFSELYVHMTGNTPVFDFYNRNQPNGSMKFICVVILNGERIEGNVKSKKKEAKVSCSLKGLEVVLKHVSEYVVPAVKFEEKTTFFEMLREHTYAKFYELCKNNALIYGFEKVIASVFLKINGNLQIIALSTGNKGLRGDKIVNDGTALIDCHAEILARRGLLRFLYSEVLKFSTEPPNSIFTKGKNALVLKPGISFHLFINTAPCGVARIDKKLKPGTSDDLQNSSRLRFKIDKGMGTVLGGASEFEAPQTFDGIMMGERMRTMSCSDKLLRANVLGVQGAILSHFIDPIYYSSIAVAELNNADRLRKAVYSRAATFKPPAPFHVQDVEIGECQVEDTEQSTSAAARSTISSMNWNLADGNTEVVRTSDGMVHDKDMSGADITTPSRLCKKNMAELMITICTLTKTSVDYPISYEELKAGSQEYAAAKKSFITWLRQKDLGIWQRKPREFQMFTIN

>AIN-1

MESLAKNFNNISIDDAAWHPNDQQPVWGNNGLQQQRVWAPPVPQPQFPNRSPWQQESNDIEPNVNEALVGLSGTGSSSQSQWGGIQNTDYEKQIWGDPPLSDVQYPLQPHASFGELNSLGGQSAPWSIGSQPAHPVWSNGGMGKESDDFWKQQQPQQHQQYPMPLLQGAWNTGMRHGPPGQGQRSGPMMQQQQDYQGGWGGPNNMGGNKQMNRPWGQDSNRGMSGMPPRNGRPNHNQGRYSGNMGGGVDVSVPPPMDMHMGGGMGGMRNMGVAPNSGHGSWKNNNAGGGQGGHGGQRNYNNRGGMGHSGSQSGGGSNMWNSTGNNGSGGSMQQFMGGDQQYNNPFPIGGAADDLTLTVWHDPNGELKKWQRDTGVSYWGDPEKQTDRTISLWIVGEGADEDLETALNRCPVPQKKGEDNQRFPFPVPAKRPIVVTGWGELPENDPNNPSKSESTIFDENNRWNDLTTEQNPWYLPNQHTSTFSSDNTTGSWVQGGTIPLNEPGTNQHVAVAEMLKNAVDKGYLDQSVTMMANLPPMVLQYVNMLLVKIPALDSVENELKQIIESSLPEDVKEVDPQNQQKYMNDSQKLEHNRLIIEVTTAKIEVQEYSKKVNRALMDAGIVQPQEQRAPASTEDYHYSFLE

>AIN-2, isoform a

MNGDGWQGPGRGGPHHMNGGGGGRRGGGGGMHNQRIQQNPHHGGMNRMMPHGFRPQQGQFMPQYPPPPPMGGMPDVMDINMRMDGMSLGSTPSGIPGQYGDMGPPPVQQWNQPAPFVPPPQQDDLYDPSGYGGGAGGGGGHHPSHMPPYGQRGNNQNQYGNRMQGGQGGYRGNQQRQQQQQPQQGFNMPPMMVPPGQPPFYGGNQPNHHHQQPSFAPSPFSMGGGGGGSQGGPHEQHGPGGNQNGGFGVSSSSMDDYSMWTDENDEDAKKKKSLRDKGLSGWGDADVSNSKPIRRWIVPEGQEEDFETAMERCPSHLKKKALNEEALRRRLTSENPQIVAQAQQQAEEEANAMMRIGRRPIVPSGWGDIPSEISTDKSDSEFDQSSSRGWDEGSISGGGSGRHPSHHQSNDNSMWNSPSVGGGQSNSETRNPFFAQHMQQQQHQQQHQPHYGGYGDNGVLDGGSLWSAAVMDGSSMVPPHPTMGVGSSASSSSIPIGMLMMSGSGNVGGGPPSSSSLLDEDGSQKKLAENLKLAVQKGHLDISLLSLPHIPPNVLDLLTEILAVIPRLDDYEDELKKLGENRPSNYDEGASKEEKESWMSKDQKNDHDKAVIGVVTAKIEVTQLSKKITEALIEAGLLPPQPQTSTSANTSTNHLNHRGGPPPGSDGGAGPSTSRSGGGGAAGGDSFNSSGGGGPPDHYYDYSFLG

>ALG-1, isoform a

MSGGPQYLPGVMNSTIQQQPQSATSSFLPSGPISSTSTSSQVVPTSGATQQPPFPSAQAAASTALQNDLEEIFNSPPTQPQTFSDVPQRQAGSLAPGVPIGNTSVSIGEPANTLGGGLPGGAPGQLPGGNQSGIQFQCPRRPNHGVEGRSILLRANHFAVRIPGGTIQHYQVDVTPDKCPRRVNREIISCLISAFSKYFTNIRPVYDGKRNMYTREPLPIGRERMDFDVTLPGDSAVERQFSVSLKWVGQVSLSTLEDAMEGRVRQVPFEAVQAMDVILRHLPSLKYTPVGRSFFSPPVPNASGVMAGSCPPQASGAVAGGAHSAGQYHAESKLGGGREVWFGFHQSVRPSQWKMMLNIDVSATAFYRSMPVIEFIAEVLELPVQALAERRALSDAQRVKFTKEIRGLKIEITHCGQMRRKYRVCNVTRRPAQTQTFPLQLETGQTIECTVAKYFYDKYRIQLKYPHLPCLQVGQEQKHTYLPPEVCNIVPGQRCIKKLTDVQTSTMIKATARSAPEREREISNLVRKAEFSADPFAHEFGITINPAMTEVKGRVLSAPKLLYGGRTRATALPNQGVWDMRGKQFHTGIDVRVWAIACFAQQQHVKENDLRMFTNQLQRISNDAGMPIVGNPCFCKYAVGVEQVEPMFKYLKQNYSGIQLVVVVLPGKTPVYAEVKRVGDTVLGIATQCVQAKNAIRTTPQTLSNLCLKMNVKLGGVNSILLPNVRPRIFNEPVIFFGCDITHPPAGDSRKPSIAAVVGSMDAHPSRYAATVRVQQHRQEIISDLTYMVRELLVQFYRNTRFKPARIVVYRDGVSEGQFFNVLQYELRAIREACMMLERGYQPGITFIAVQKRHHTRLFAVDKKDQVGKAYNIPPGTTVDVGITHPTEFDFYLCSHAGIQGTSRPSHYHVLWDDNNLTADELQQLTYQMCHTYVRCTRSVSIPAPAYYAHLVAFRARYHLVDREHDSGEGSQPSGTSEDTTLSNMARAVQVHPDANNVMYFA

>ALG-2, isoform b

MPGDSLTSSSFMPDGGAETSSSSQLGGSAHGAIGTKPDAGVQFQCPVRPNHGVEGRSILLRANHFAVRIPGGSVQHYQIDVFPDKCPRRVNREVIGCLISSFSKYFTNIRPVYDGKRNMYTREPLPIGTEPMNFEVTLPGDSAVERKFSVTMKWIGQVCLSALDDAMEGRVRQVPHEAVQSIDVILRHLPSLKYTPVGRSFFTPPGVMKPGMQMHQESKLGGGREVWFGFHQSVRPSQWKMMLNIDVSATAFYRAMPVIEFVAEVLELPVQALAERRALSDAQRVKFTKEIRGLKIEITHCGAVRRKYRVCNVTRRPAQTQTFPLQLETGQTIECTVAKYFFDKYRIQLKYPHLPCLQVGQEQKHTYLPPEVCDIVPGQRCLKKLTDVQTSTMIKATARSAPEREREICKLVSKAELSADPFAHEFGITINPAMTEVKGRVLSAPKLLYGGRHRATTALPNQGVWDMRGKQFHTGMEVRTWAIACFAQQSHVKENDLRMFTTQLQRISTDAGMPIIGTPMFCKYASGVEQVEPMFKYLKQTYSAIQLIVVVLPGKTPIYAEVKRVGDTVLGIATQCVQAKNAIRTTPQTLSNLCLKMNVKLGGVNSILLPNVRPRIFNEPVIFLGCDITHPAAGDTRKPSIAAVVGSMDAHPSRYAATVRVQQHRQEIITDLTYMVRELLVQFYRNTRFKPARIVVYRDGVSEGQLFNVLQYELRAIREACVMLESGYQPGITFIAVQKRHHTRLFAADKADQVGKAFNIPPGTTVDVGITHPTEFDFFLCSHAGIQGTSRPSHYHVLWDDNDLTADELQQLTYQMCHTYVRCTRSVSIPAPAYYAHLVAFRARYHLVDRDHGSGEEGSQPSGTSSEDTTLSSMAKAVQVHPDSNNVMYFA

>DCR-1

MVRVRADLQCFNPRDYQVELLDKATKKNTIVQLGTGSGKTFIAVLLLKEYGVQLFAPLDQGGKRAFFVVEKVNLVEQQAIHIEVHTSFKVGQVHGQTSSGLWDSKEQCDQFMKRHHVVVITAQCLLDLIRHAYLKIEDMCVLIFDECHHALGSQHPYRSIMVDYKLLKKDKPVPRVLGLTASLIKAKVAPEKLMEQLKKLESAMDSVIETASDLVSLSKYGAKPYEVVIICKDFEIGCLGIPNFDTVIEIFDETVAFVNTTTEFHPDLDLDPRRPIKDSLKTTRAVFRQLGPWAAWRTAQVWEKELGKIIKSQVLPDKTLRFLNMAKTSMITIKRLLEPEMKKIKSIEALRPYVPQRVIRLFEILETFNPEFQKERMKLEKAEHLSAIIFVDQRYIAYSLLLMMRHIKSWEPKFKFVNPDYVVGASGRNLASSDSQGLHKRQTEVLRRFHRNEINCLIATSVLEEGVDVKQCNLVIKFDRPLDMRSYVQSKGRARRAGSRYVITVEEKDTAACDSDLKDFQQIEKILLSRHRTVNNPIEDDSDRFEEFDVDSQMEPYVVEKTGATLKMSTAIALINRYCSKLPSDIFTRLVPHNQIIPIEENGVTKYCAELLLPINSPIKHAIVLKNPMPNKKTAQMAVALEACRQLHLEGELDDNLLPKGRESIAKLLEHIDEEPDEYAPGIAAKVGSSKRKQLYDKKIARALNESFVEADKECFIYAFELERFREAELTLNPKRRKFEDPFNYEYCFGFLSAKEIPKIPPFPVFLRQGNMKVRLIVAPKKTTVTAAQLQEIQLFHNYLFTQVLQMCKTGNLEFDGTSNAPLNTLIVPLNKRKDDMSYTINMKYVSEVVANMENMPRIPKDEVRRQYKFNAEDYKDAIVMPWYRNLEQPVFYYVAEILPEWRPSSKFPDTHFETFNEYFIKKYKLEIYDQNQSLLDVDFTSTRLNLLQPRIQNQPRRSRTVSNSSTSNIPQASASDSKESNTSVPHSSQRQILVPELMDIHPISATLWNVIAALPSIFYRVNQLLLTDELRETILVKAFGKEKTKLDDNVEWNSLAYATEYEEKQTIIVKKIQQLRDLNQKSIEDQERETRENDKIDDGEELFNIGVWDPEEAVRIGVEISSRDDRMDGEDQDTVGLTQGLHDGNISDEDDELPFVMHDYTARLTSNRNGIGAWSGSESIVPSGWGDWDGPEPDNSPMPFQILGGPGGLNVQALMADVGRVFDPSTASSSLSQTVQESTVSPPKQLTKEEEQFKKLQNDLLKQAKERLEALEMSEDMEKPRRLEDTVNLEDYGDDQENQEDENTPTNFPKTIDEEIEELSIGARKKQEIDDNAAKTDVLERENCEVLPVAINEKSRSFSFEKESKAINGRLIRQRSEEYVSHIDSDIGLGVSPCLLLTALTTSNAADGMSLERFETIGDSFLKFATTDYLYHTLLDQHEGKLSFARSKEVSNCNLYRLGKKLGIPQLIVANKFDAHDSWLPPCYIPTCDFKAPNTDDAEEKDNEIERILDGQVIEEKPENKTGWDIGGDVSKSTTDGIETITFPKQARVGNDDISPLPYNLLTQQHISDKSIADAVEALIGVHLLTLGPNPTLKVMNWMGLKVIQKDQKSDVPSPLLRFIDTPTNPNASLNFLNNLWQQFQFTQLEEKIGYRFKERAYLVQAFTHASYINNRVTGCYQRLEFLGDAVLDYMITRYLFEDSRQYSPGVLTDLRSALVNNTIFASLAVKFEFQKHFIAMCPGLYHMIEKFVKLCSERNFDTNFNAEMYMVTTEEEIDEGQEEDIEVPKAMGDIFESVAGAIYLDSGRNLDTTWQVIFHMMRGTIELCCANPPRSPIRELMEFEQSKVRFSKMERILESGKVRVTVEVVNNMRFTGMGRNYRIAKATAAKRALKYLHQIEQQRRQSPSLTTV

>XRN-1

MGVPKFYRWTSERYPCLSEVINESQIPEFDNLYLDMNGIIHNCSHPNDDDVTFRITEDEIFVNIFAYIENLYNLIRPQKVFFMAVDGVAPRAKMNQQRARRFMSARTAHTQLAKALENGEIMPSEARFDSNCITPGTYFMTRLHQKLDDWIKTKSATDSRWQGRKIILSGHNVPGEGEHKIMDFIRTERAGSSYDPNTRHCMYGLDADLIMLGIVSHEPHFSLLREEVTFNTRPRKNDNKATKKPKRTDSDVKKFHLLHLSLLREYLAWEFADVKDSLPFEYEMERIVDDWILLGLLVGNDFLPHLPNIHIHDDALPLLYSTYKKVLPTLDGYINEAGYLNLSRFEAFLAELALNDKTSFMERLEDEQFMESKRIRVVDPEQQELAEPELVAFESSDVEDEAGAEESKDEAGGAGAEDDEDDAAFVSDHEEDGDDLEPGSGSDELLLSNLDAQLLEDEFNDELATLALSGMNDADFANDVEACWTKTINNQFKRHKKTYYSDKLRYKNISKQQLREQAEGYVRAIQWNLHYYYHGCVSWSWFYPHHYAPFISDVRGFVGMRMEFELSEPFHPFEQLLAVLPEASADCLPRPLRELMSSDPAKSPICDFYPANFETDLNGKRNEWEAVVLIPFIEEKRLLEAIEAKRSRLTSEENARNSHGSHIQCISSPEPPNSQGQWRTVRSEIPQDSFRIPRSQVKWGLLPNVKMDVYFPGFPTMKHLSHLGELRFANCNIFGMASRKESMVLKVENERAHTDIIELGSELCDEEVCIDWPILKVAKIDSIWGGDDKIVRKCDDEIIVKDMSEEEKRQWQAHANQLTEHLMTRFAIEIAAQKDSKGKHIRPAVAWVRKFTGLVYEAQGGGKEPSVLKACKQWSSPQQLMPVLLPLVVKDVLLENSRVLADLPMKQAYPKQSIVWITDPKFALFGMPGMVNGFSNEKSADCRIEVIGMSTQNKVDKMEALRKKMEQKSLRWMGGYDCARQCQVDTRLFARITGTMFLWNEPRERVEKGQQISSSDSKINCGLALKYSRRDLCVADYTDRTEHTNQRGVTNKVWFYTNLATRLVSEYRRKFPDVWKYLETIGLTQQDDVYYTEDIWSNEKTREKRFGELSEFLGGLPSLEAEQLKCGTVYADRQLITEIEMILAEPDEKKPVMNKYMMAPGALFRYELYNGKVHADPAADFQILDRVALMSSDTKVPKTTQGTVVGIHDDKIDVFFDKPFDGGQKVRGSNEAAAIRVPQSALLNVTFGIVRKNVQHKKQVEKALTGAYVPMPAQKNSAKNEPAPSTSSSNSNSTNNKKNSSKKEKNKQKPTKTDEESPEALTDSLNKLLKIKPPSAEATSGKQVSLMELLGGGGAAGAKKPAQNAKTLAEIESDSSQSASKSSILQQLSAAQKAAAPKPKKEASQKKQKPQAAPPQKVEILRKPTPPPPPPAEEQKPAPAPFGAPTPPPQFPFGPQPLQFAPHPMMMMGYPMGMPMGMPIGGPHHYQQQQNQQEKRANHPTLTDFKPSAVHRRQNCHQRPSNQPPKVTMITKRTSSPTPPEKEEDAAPRALPSSPPPPSKSAAEEKTTTKKPRKKKQSRLGSNFSNPTASS

>XRN-2

MGVPAFFRWLTKKYPATVVNANEDRQRDQDGNRVPVDCTQPNPNFQEFDNLYLDMNGIIHPCTHPEDRPAPKNEDEMFALIFEYIDRIYSIVRPRRLLYMAIDGVAPRAKMNQQRSRRFRASKEMAEKEASIEEQRNRLMAEGIAVPPKKKEEAHFDSNCITPGTPFMARLADALRYYIHDRVTNDASWANIEIILSDANVPGEGEHKIMDYVRKQRGNPAHDPNTVHCLCGADADLIMLGIATHEANFNIIREEFVPNQPRACDLCGQYGHELKECRGAENETDLGDDYCKPEQREKNFIFLRIPVLREYLEKELSMPNLPFKFDVERALDDWVFLCFFVGNDFLPHLPSLEIREGAIDRLIKLYKEMVYQMKGYLTKDGIPELDRVEMIMKGLGRVEDEIFKRRQQDEERFQENQRNKKARMQMYGGGGRGGRGRGRGRGQQPAFVPTHGILAPMAAPMHHSGESTRQMASEARQTAMKFTNDANETAAANLKALLNVKGEESPADIASRKRKAEQPLIKPEEEEDEGPKDDIRLYESGWKDRYYRAKFDVGSDDIEFRHRVAWAYVEGLCWVLRYYYQGCASWDWYFPYHYAPFASDFETVGEFQPDFTRPTKPFNPLEQLMSVFPAASKQHLPVEWQKLMIQDDSPIIDLYPADFRIDLNGKKYAWQGVALLPFVDETRLLATLQSVYPTLTAEEKQRNTRGPNRIFIGRNHKSFEFFQQVAESKSDDLVPLDPTLLNGVSGKIAYDSTATAPGLPFVSPVNHDECQDLPTNCGICVLYEDPEYPQDYIFPALRLDGAKEPEKTLKPDDWNDRRDGRYQPQVGFNRNAPRGSLDQSGHRQVHHYVRGGGGGGGGYRGNSYDDRRGGGGGGGGYNDRQDFGRNYGGRDGGGPQRYHDQQQQRQGGYQGGGYGGGYGGGGGGGGGGGGGSYHQPYNQDQRRGGRGGGGGPPGYQRPPYRGGGGGGYHGNSSWR

>CID-1

MTDEQRSGSRRRGGALGGGRGGNSQNSGRKRSSNSPADSASSSQKTSRNPSQNRPPMNIVPQKVTILKRNQDSQPTSPINRNAPPGPLVAAVQPLMAVELGDSGASLKGWNSNPTTPEKTTINVAGRQAEANKKQTQEYYFSKNAGSAVSAQTFQETVQYPMSSFPLEKNVPIQIDNNFTLLITPTTSKCNGLFIKVKLQHGDDNITTGALLKLDADEKTIRKSCRETIENLLKAKSSSDRFPFSKCSEGFLTSCLIKKLVDRLEAFPEAIYYCEKCDYHINTIGHAKAHLESSTHFDDVKRQEQREHLLKIIPPPTEAHLESVEKVLEGTLKSYQQVRQNGIEIGETIIYYLSTTVFPSIGLQDVILRPFGSVTYDVTLPDSDYNVAYTMSIPEGTPIFALLEQVRKKIADDGHPADHSMEMGTPSTIIFTFKGVRVKLCWMSCFNHRIQLHFSELMKTYVNLREEVAQFLQLIRLWATKAGLDSKNKPRIGLPRYGFDIMAIHFLQQIGCLPVLHELFEAGPETEKIEEMSAEKTAKLQSEEAGQRRMRLMSRYEKNTEKIGTKFDLKKSWNLAELFIGFFRYYVERHRDVVIQITQLIPMSRDVSRWNKKILHVVDPFRGDNVLSIPKVSTWQPFFFNCLLTSFVSFSIPRTKNGPLIEVSFVHNKTIKPGKKFKDTPKKATVEAPIPLTQIMEEHYQEPVYEIVNDKDHLKYMKELKERLMIDGILVNERKPIDYHTDDHRECVYGRNSLLRFKRVLKPGLNGKMAASMDGLSFVTKKGKKFINRWKKANVIILSEDSSEIDEKTKAADSNVSEIAEKLEETLKISSQKNIDVAVTSMPVSTEIKPEPITKLSNEAPVKIKNVAQPQCDLIEAPQESLMNESMMESSFDETESPMKTPIVQKPVKQNATFGKSTISTVIPDKQFCSEEFFIKLNSNLKDIVSKSKSLKPSDFHYEFSTDLFCGDFEMEMKCTHCDGSHCVENCPMMEIPPIKKYQARTPEDLKDIDDMIDKYYHENILDERRLKMLDHKIDELQSFLRKNYREDVTLTTFGSVMTGLSVNCSDIDICLRFGDGDVPPKDLTAKEVIQKTESVLRKCHLVKRVQAIVTAKVPIVKFQVKLSNGAIIDVDISYYNILAIYNTALLKEYSLWTPDKRFAKLALFVKTWAKNCEIGDASRGSLSSYCHVIMLISYLQNCDPPVLPRLQEDFRSDNRERRLVDNWDTSFAQVETSLLQRWPKNKESCAQLLIGYFDYYSRFDFRNFVVQCRREMILSKMEKEWPRPLCVEDPFDLSHNLSSGVNKKMFVFIMKVFINSRAVFMSEKPPMNRDHTFLSSYQHQLLRKCNQGSAPTDRQCHQCHRIGHFVESCPQRALAKEARKRFGSNSTNSSYRSNDSGRGFKNGEEGGDRLTYHKRTYYHRSYNK

>CSR-1, isoform a

MQSDNVGRGRGSRGGSRGGSGRGRGRGGYGDSYRPDLETRETHVTYQGKVKYEGGDRHDFNYEKTPTGSYRGSHRDGQEGSRGNRGSFRGGRGGYDRRRDDEESKAENRFGGYNRGANGGNYRGRTGGSGRGGSTQTTTVDPKYNGLILENSAEPKLKGLNSDMNQKQNPRLALNIFGLELSERTIFRHVVQMKLIDRQHNKEYILTTMSARGRGNRATKQKDNFILLDILLKQWAAKKGQQNLPAFAYDGAQSLFTLEGISLMVDIKKEDALEIPELSNFLKDSISFLSGDLEISCEPDLEKPSFVQTELNEWSDPRFYAYLDIVTSQSAIRSERYLSQSKGLYVHTQSLEELRVKWAVAAKGIHKGCRIVGANGPLPILELDPQSTQYYASIPLSQMLQYAFPRDFPPNRIVNPNMKLQRAVKLLLKDLKCNPYYDDKQIWATNTITVSDVDYNAPKDPEFRQKYPNLKFPMLPAVQCGTGPHKRLMPLEYLKVLPYQSIDRRVLEEFELTPRANAPNERWSTLQKHYDQFGFNDQVMKDFGVQICNDPFNNVSEIDGERVLAPSVAYADPVHVDDEKRDWKAQDKKFVTPATIDHLMFVLVAGYTRTWDADCDATKFVAKAFMQRCKDKGMHIGSYSMDQHNGERGSENFLTSVFKNLVTHPNYRDSSFTPFVLFISDDVPNIHECLKFEERMSDIPTQHVLLKNVKKMRDNIEKKSQGGRRAYDLTLDNIVMKANIKCGGLNYTADIPRDLACWNEVSTFVIGMDVAHPDRNAAREGNPSTVGLSCNSAENPYSFIGDFLYTDPRREAIQDEILRKFTDQSVRNFAEIRGFPKKVIIFRDGVSFGEETAALKEVEIIEQTIKTAAKSMGHSDYAPKVLAIVVKKRHHTRFYAKGGHHGNMPINPLPDTSVGGDIAEYGKRQIFIQAFRPVQGTAKVPSFLVIRDDEEVSDEHVAKMVCAVCSLHQLVNSPTSIPTPVYVAHELAKRGTGLYKAYRFKNGELFDDWETLTTQLSYSTLDRLSKVRVV

>DRH-1, isoform a

MRKKQCSSILSLYDKEIILCLEPIYRDPEKGDGFSELLPLGRIDELKIQSENAQEFSKQLYHDLKNSILSNADDERLYKDIMTYLQTYLPKCTVHKLLNCSNREVKLSDFHYILDHFEGFLRFIEPKVVLAYLDSYPQYIDAVAVLRKEIERNEEDNQDSDFIKKLILRTVPLLGEQAVYDIMYTISEKSSNNLDVEAKQFIAKVLRLKNDGFLRFYQIINASRRQLNGRIYICPVHESATEMMVYLGTAALNTNRYRMINIRVDNIVQENSTPRLVIESVRQRIHRQRQLCLRNYQEELCQVALQGKNTIVTAPTGSGKTVIAANIIKEHFESRSSEGKRFKALFMTPNSMILNQQAASISSYLDHVYHTQIIQGSDNVPTRNVIQSKDLIVATPQMIVNLCNEHRNSLDDESRLDQFFLSTFTIIFFDECHNTVKNSPYSNIMREYHYLKNMGNMPEGHSLPQIIGLTASLGTGDKNDCLQVRNYIAGLCASMDVKDLSIVKDNLEELRGYSPIVPDKVLLCERSTDGPIGMFTNRLTLMMQEVEGLIRTALRNEHIGIEQRRQIETTERDFRPDSSFLDPPADKEHAGYQNWVCNQMNLVSGTSFRETGTRTIINEALDVLKECFCTLSYNINFHPEVALNYLKDEMEYRTPNFTVNMIRIWERYHNQLVGTGSAENPMISKTVQYIVEQNLQRADSRTIIFVRTRYEATILNKVLNSNEELLMLGIKSEWMSGLNKSTASSADISASKQKQMEKLKMFADGEIRILVSTSVAEEGLDVPECSLVIKYNYATNEIAHVQRRGRGRALNSECVLITNSIALRDQESNNRDKESLMSETISLIQNSPAEFRKCVDEESNKIWPRILREDTDKAQKIEEQINRNIVYKIICKKCEAILCTSKDIRSRNTQYLVCDPGFWSLVRKTRLTDEQQALIKYNATGSINCRRENCGLKLGQLIEVNTVDLPCLSALSIVLLVEGTDKRIIVKKWKNILDKYFTPTEIRQLDVQTMRDADQARTPMVFEHHANGEVVNLIREA

>DRH-3

MQPTAIRLEDYDKSKLRLPFESPYFPAYFRLLKWKFLDVCVESTRNNDIGYFKLFESLFPPGKLEEIARMIIDEPTPVSHDPDMIKIRNADLDVKIRKQAETYVTLRHAHQQKVQRRRFSECFLNTVLFDEKGLRIADEVMFNYDKELYGYSHWEDLPDGWLTAETFKNKFYDEEEVTNNPFGYQKLDRVAGAARGMIIMKHLKSNPRCVSETTILAFEVFNKGNHQLSTDLVEDLLTEGPAFELKIENGEEKKYAVKKWSLHKTLTMFLAIIGFKSNDKKEKNEHEEWYYGFIDAMKNDPANRAALYFLDKNWPEELEEREKERDRIRLTLLKSQRTNEEAVGEDVCTTIRPQPKDSGYNPDAVVTELVLRTYQEELVQPALEGKNCVIVAPTGSGKTEVAIYAALKHIEERTSQGKPSRVVLLVPKIPLVGQQKDRFLKYCNGMYEVNGFHGSESSVSGTGRRDEVIATHVSVMTPQILINMLQSVRQNERLYVSDFSMMIFDEVHKAAKNHPYVLINQMVQEWKYEKPQIIGLTASLSVKVDGQKDENQMLNDIYNMLALINAPHLSTITRQSSIDELNEHVGKPDDSVELCLPAKENILRDYIERYLNHAHGKFLEELASMSKSTGRNNTIPPNMINTFKKNQPKNYEYYDSLLQGIIQELNKLNVPEKWNSQTWAKYMKVYLEARGIVDLMPAMVAFKYMEKAIGKLNESHSETVEYSTFIKDHDTLKQTIQSVEPEIVLRLKNTLTNQFHVAPESRVIIFVTQRSTAQRVSDFLNESKVLDQFGNYGEQMVGYVLGTNKQGAVQQTSQEQQLTLDKFNNGRLKVIVATSVVEEGLDVTACNLIIKYNCSSGSAIQLVQQRGRARAKNSRSVLLSVKSSINETETNALISEKYMRLCVKKITENGEKQLAAEVKRVAELNAAERKRNLEEQLNLRLRHENKIYKLMCSNCSKEFCKSIYIKKVFSNYMVFDPSVWRFLHVESKRKVSKYLSEDNQPLSDIKCFHCKLDVGRAYKIRGTYLPQLSVKALTFVQESDYSSMTKAKWSDVEQDLFYISEAIEDDFRIMLNALSDTEENIEKKIVLDLDSRQHNKQLEMKRFHIQQEPPTKGVAPEAQ

>DRSH-1, isoform a

MSDEKISMTLNFPKHKRARRKKYQKEYQERHKEEMMQQLGRRFQNQPSTSSAPPDTVEKIPLPTESTSALPFGDSPRLTEKDYETNYMIDPPVVSTHSAELIKSNRVVIKAEEAEKYMMIKAKSTTSKILQDFQTKILETVKTKRRLQADVPYIIHPCHSMKGRKTPKQKGGDESFTASDVSDDSNDSQDEASTSEPTNRQAPEADKTGEVKDEKQTCNRRNQQRKAKRLRNFEEKERQITLLKKGIDRKKTHPNGIHPDISFNEKGLGNEGPECRCPEPIKTCGLKHGYYAGEDKAIDCKKSNGENLHYYTLRVTPLPSENQLYRTHMAINGEEFEFEGFSLITHAPLPDCMTRAPICKYSMDYEFQLVEEFMPDECFDPEDCDMLFEYIFHEIFEMLDFELRPKHIPSDVESCPMIHIMPRFVQTKDDLVQLWSSKTVLAYFTSKGSSEIMSPEDVNRLCDAQIDQFTRNTSKHKQSIVLNTKFKPSAIRADWFERDEEKKEVYVVHNAIRAQTYTAISLPRIAFLEKTLNKMIQEKQSSGVYNKDFEKTKNELEHLKRENRSARNLKLREPVAGFIETGLKPDVAAHVVMTILACHHIRYNFSLDVFEEVIEYKFNDRRVIELALMHSSFKSHYGTPIDHVKNMITNCGYRRKYGAEDKREKKRVAGIMSLFNIMKGTSGGEPILHNERLEYLGDAVVELIVSHHLYFMLTHHFEGGLATYRTALVQNRNLATLAKNCRIDEMLQYSHGADLINVAEFKHALANAFEAVMAAIYLDGGLAPCDVIFSKAMYGHQPVLKEKWDHINEHELKREDPQGDRDLSFITPTLSTFHALEERLGIQFNNIRLLAKAFTRRNIPNNDLTKGHNQRLEWLGDSVLQLIVSDFLYRRFPYHHEGHMSLLRTSLVSNQTQAVVCDDLGFTEFVIKAPYKTPELKLKDKADLVEAFIGALYVDRGIEHCRAFIRIVFCPRLKHFIESEKWNDAKSHLQQWCLAMRDPSSSEPDMPEYRVLGIEGPTNNRIFKIAVYYKGKRLASAAESNVHKAELRVAELALANLESMSFSKMKAKNNSWFQNMRRRLEQDTSD

>EGO-1

MGDEGYRGWIKLEIPCSLPERQMGPIVKCHVAKLEPALNEYNIKVLTKGQVQVVEEQDCEPFYETNYEVATSRFSHDLIAAIQTYLKDLSTDHLMPFQRGNLVLHSSDFWSSELTCHLVDIPLAAVFFGNIQGGTFINHWEVSFWDDVRRRKSARTRNTEPTQADKIGMNQIKVEFEFDKIDFMTVHFKHFENDFEVADKDAKRTKQTVTMYYQITVRRTSIRRIIVDPVVQDCNGSDRIRVHFELNCPVLIRRAYRTAKQESENRHSVPHYRRYLVINRGRSANQYPTAKAITDSPVFTIEFDQSVGLNEIYRLLSRLRIRTGVSIEFADIPSIDCLIWRENPYNRWTFLNNQHLSPTHFSAPIYRDFITTAFPKKHEVCGSREVDTNRERKFAITYLLECLISRGAVVKDQILLDEGIWHRFLEVILHYYTKDDKLCEAGLEDLVHMIDGRKRIGSLIKCFDRICQTRQRNSLVNGLTTEEMREGYQRVRKIIFTPTRVIYVAPETLMGNRVLRRYDHDGTRVLRITFRDDDNQKMRTNKTSTMLEKTVNQYLKNGITVAGRNFGYLGSSNSQMRDNGAYFMEKYSSSQCREYERIYQIKPPITFNPKIQAARKNLGRFETIDNIPKMMARLGQCFTQSRLSGVNLERCTYMTTYDLTGGKNLKGDEYTFSDGVGMMSYRFAQMVSEVMDFGKGVPSCFQFRFRGMKGVISIEPLLDNLRQWSISYNISKPSDDSSWSLNCMFRPSQIKFISKRHPRDQVEIVKYSSPVPVALNKPFINILDQVSEMQSLECHRRVTNRIEELLDRQMLSFAQQMVDETFCRNRLKELPRRVDIDYLRTTWGFTLSSEPFFRSLIKASIKFSITRQLRKEQIPIPCDLGRSMLGVVDETGRLQYGQIFVQYTKNLALKLPPKNAARQVLTGTVLLTKNPCIVAGDVRIFEAVDIPELHHMCDVVVFPQHGPRPHPDEMAGSDLDGDEYSIIWDQQLLLDKNEDPYDFTSEKQKASFKEDEIDDLMREFYVKYLKLDSVGQISNSHLHNSDQYGLNARVCMDLAKKNCQAVDFTKSGQPPDELERKWRKDEETGEMIPPERAERVPDYHMGNDHTPMYVSPRLCGKLFREFKAIDDVLKISEERDEQVEISIDETIKIDGYTEYMASAKNDLARYNAQLRSMMENYGIKTEGEVFSGCIVDMRNRISDKDQDDMSFFNTNQMIETKLTNLFKKYREIFFEEFEGGWEGNTEAFSRYGRDSNILQRQCRAPTVQMMKKAVAWYRACYEEARITRENKKLSFAWLAYDVIAKVKQDKSLTSDEVKMGGANPLYTMLDDHRSQYLVDNSRKFEAFRQFSTPKTSGEQVKRAHRIIKMYTETYPGLDAVLFMLDEWARISNLFENQSLREYHLSLLFILFATRQFSSVDGNAAKFFNKVDEKSYKQSKTIGDFEPSLYIEEKGKSQMMVKFLEFLASRKFRKMANLSFCALDFSSIFMRGEWQIFHLAALKTYYNVLFNLRFEELPVSTDPTTTVRSIIRENEPFVIELPANCDRSLVHRKLVEHTGVKEIFMRNMEKSVRSSDDVQKINMRLLVSTRGTLESMYKLRQLVAVKVPIKTYVTGQDVSTQMARLCYEKIVRGHINI

>EKL-1

MIAVGLRLTDEVFPNPEEPIAKLPTGNENPEMLLDGVNKDGKTRIKRIVLSRSANVEFLRAESPSRIWVRLTNHITDSALTFREPFELTQKTTFKIGDYALAPTDERVYRRCRIVDVCRNNELLKVFFIDDAVIAWVQPECLGELDQHYMYYPWQAIQVSMFGATPTVDVNRSTEQLWSPIICDQLSKVLEQFFILRVEVVLSTVVFNDYAKPIPVNLFGIEADIDEKDQIRRAIEIGPILERQEPVGISFPEFFDAAYHQVFEVAERETIPNEALEIHRSFPTDWKKTSISEDQMKEEKEKALFSKNSLIPEMEHSDWDPRQNSIEMLSIDQLEEKFKFPYDSDVAPAIMLAVEGRCTKSPFEWYARPIVKTGRNANGEMVIWNDEEQLKEVDPVDWMIYGNDQLLSMAEQLDTYYSNPKNRKPLKAEEIQSMRKEKRDVFAVCAVNEEKAMYTGEWQRVLIVECDTFAEVRFLDSGGRDMVLTGSLYKIHRQHCRFPPMCLRLSMHGITSSESVVRKWTSAETSRFRTCLREDVPIFINIDDIAPLILPPDDKKDPRAHMAKHVLMVSDVSYMDESKSLLDRFTDKDEAVRAAFSQKEPIEWPN

>EKL-4, isoform a

MIGDVHQILQSTEAPKDATKKPPKPGTSAPRKPEGMKRELFNLMSGKDLTAVMPTDVKKTYKQKFQTGFRSVRKYKWMPFTNESRDDGLMLHHWVRADKVEAMQPYPFSRFNKVIDIPIYTDDEYENYLKIAKWSREETDYLFDTCRMFDLRWPIVYDRFDCKKFNQNRTVEDLKERFYSITYELGILRDPSSSPTAYDAEHERRRKEQLNKQWNRTAEQLQEEEDLTAELRRIELRKKEREKKAHDLQKLINMSEQQPASPSAGGIGGAASAKRKNAFRTKAGSISTTATTFFNPLDISVTALRFSEFKSSGAHFRCQEMKLPTNIGQKKLKNIEVVLEKCKMEMNPVASEPIMKTYNDFRSQIMLAQELKSAMQTAEFELESIRTRMQENGKDFDIEPRFRISQLPEGGIDDDFIGGKGQPATNRRITSYIDASSKDLTAIASRKRKTIATTPTITTTTSSSFVSSSNSPSTSAASDPKRIRKI

>EKL-5

MLPVANNPEEQANEEELRDQAADIPQARNHNLRNHARRHREMFERARANRRLQADRGAQAARERGGPAAGRAQLGRARAREALARANNARQALQPDAAVLEALDEINGIDFDDEEVPNRRDVRQDPQVLEFANLFPRLIALDPHDAQLANQNLARMARHLAPENYIRVARREQRQSFMCGICKSSVHNVAFLNHLDTCAASKGACKIMKDMEYHHFLSSTYSLRQQLYKLRERLELEYFEARVNPERLEHMECAGCITCAEHLLGACMPNMQRDVYQIHANKIEKTVMAHFEQFLELTKEDGLGEIISKHTAHFDSIQDCIEDGPTDFDELQETQNRRHGTWYEFRKDTVEEQQLEKNKYLEETIEKHLVKYRSHRTEVTKLIGLFMDKMLACIEELKNSPRLARIIQLRRVHCIDPIRGQVPLEGEAEEIEDDEEDINAVVQAEEFLQLGII

>EKL-6

MFFSNKSIMTIPSKILASKPPDAQKEIKIQMPVKTNPVYRARTNTFPLFFGLHYQHRSTFRLICDILYLAIYAHLLECIAVYAINYMYPNLLWETFGYTIPRILQILLVFHSIPVIGSLSRTVFGCRMSVFFTHPLPESDSSGSCHQTKQPPFQSCTNSPPCCLIILTIFLKHILLSTMLTKNELIHMISELITPPAETWKNDPFELLVKKCPKDLFEKYVDTPNEDSIPSTSDDVRTVNADVRIYYSQILGGLYLDLANLLKSERANCSDEYQLLSLQDVTLVRKSFEFFLLTGILPFMEPGAGLGAASRSTFIKSWKLYDGNKETCIEKLEFAAKVIINLLESNESLIGQFLTKFIDDVLAVRFQLLQLKIDKYELNFAELVSKLQIDVLFGSLMFLTQDKKTAKTPMWLKVACGKQMTKILVGKDGLSYLLQYYRERAGDTWTDNLPLTKQVSWHLATVPKMFRHPLNYHEIISNQFFELLWSQKIPEQNTLNVFINYVEELHTRFALNANLTVFDKILNFWEVLDKKIQDKTVMHSEKVENYSPNFIRNLQLLSQLQLSADVKRTRALSTCLFACVEQIPYIKDILKSALDSVLSIGYIIYQFVMTPSHRVALEKKFVTSSKIQEIGGDSNGNDTPFDEMWLHGIESTDEGVARRLETAFFVIDNVLTSAQIRPILEMMNMALDDFLKVSEKEREDDQARFVQLDGAKLFSSSHAHLVVGACFERIINLAQDSGFSQEECVQLLRIAESILNNCTGKFMRMANRKRNVDVFRMTPTEKKEFECSRDSAKMCLPMISTVFMLTQTTTRMHDVHIRSMEAMANFTKAADAFPSEDVSFNLAVDEAKKLLRDLKIDVNQVNAPTLPQRTDRQRYNQQVDVCNEWIEELHDDEPAIKGGALMQIARVFRNRTYHCQRLFEYGVFDAVKDMVADDDSYVYLSAINCLCEMGLYNKNMFEDTIEYYEEMSNQPQKDTKMIIRVGRVAEAIGKLLLARGEISVTYFDRLATLFMKGIDEPEEILRASSCGAFGNLMIATRGRGSEKWLGQVFHKIVNIIRVDRSPLVRRSAADLIRHSLQSTGRDMLAILREHLLDLHREIRQLHRTDRDETVRLHAQLCLEEIEAALRQNQEDTERGYHRRIRF

>ERGO-1

MSYNNGGGGGGGGYRNDRDDRYHNNDRQNYRSSDQGRSGYNDDRRDNRYDDRRGSNNDRGCYDQHDRRGSSNDDRRGYRGYNQGGGGYQQQYSQDARYGSNQRNDNYGNNRGSHGGANMYSQNGGNRGGGGGRVGGGRTAAGMSNPGDLVGGADQPIHSVSKKSLRHNAQEFAVRPKTMVQDKGLGQKTTLLTNHTLVQLPQEPITLHVFNIEVFINGKSSNKRELCGPRFWEILKENKPTFGMPNQYIFNDVNMMWSTNKLRQSEGRTNNRRMNFVWKYVKQIKFGGNIEDEETMQLLSTLIDAIATQRARLPLAPPKYTVFKRLTYLICEEAYEPELPDVSLCHKLRIGTDARVGVSIAIRTNLRAGITACFDLGHTLFTRPAYPLVRLLCDIIEHSVVLDEAFEMKYDAALRACNVSDENLRVMTQILTKMTLQLSTETGDYVGEDGEVIVRPAPTIRNPGRNFKFVGLGAPADRYYFTSDGVELTVADYYLQKYNIRLRYPNLPCVLKKAPEQCGNKHSAMPLELVSYIVVPTRYGGFTMPDMRADMINKTTYTAQQRGKLLQHIIAQKSLSGIEPPVSNNDDYMKKHKLVMKREPIRVKATILPPPTLVYGDSVFHDEHHIGEWEAVTHDPPRQVLDGAVFRRKLYKSSEQPLMKRLMGSILLIQSPRQCRDFDYNQQGYHAIMRAIEDSGQPVLWADENKHSAVIQGELQFNQNQHGIEVIEQFLQNIKSTIGEYERDGEVIVPIVFAVFQARATVYSGNNNEYNDYNVLKYLADNKYGIHTQGILEKSLGVVGPSPKNCALTRLMVEKVLGKVGTTHRKLERGGAHKTWTIFTDPAKPTLVLGIDVSHPSTRDRETGNVLQKMSAATVVGNIDLDVTEFRASSRIQDTGVECLIDFSKEIDERIGEFIDHTGKRPAHIVVYRDGLSEGDFQKYLFEERVCIEERCLKIDTSFQPSITYIVVTKRHHTQFFLEDPSQGYESQGYNVLPGTLIEDAVTTNKYYDFFLSTQIGNEGCFRPTHYYVLHDTWTGKPDSFWPTVTHALTYNFCRSTTTVALPAPVLYAHLAAKRAKETLDGINTYKSVNNIYCDLESFGDLCEVNKDMNVNEKLEGMTFV

>ERI-1, isoform a

MSADEPSPEDEKYLESLRDLLKISQEFDASNAKQNDEPEKTAVEVESAETRTDESEKSIDIPREQQLLPSERVEPLKSMVEPEYVKKVIRQMDTMTAEQLKQALMKIKVSTGGNKKTLRKRVAQYYRKENALLNRKMEPNADKTARFFDYLIAIDFECTCVEIIYDYPHEIIELPAVLIDVREMKIISEFRTYVRPVRNPKLSEFCMQFTKIAQETVDAAPYFREALQRLYTWMRKFNLGQKNSRFAFVTDGPHDMWKFMQFQCLLSNIRMPHMFRSFINIKKTFKEKFNGLIKGNGKSGIENMLERLDLSFVGNKHSGLDDATNIAAIAIQMMKLKIELRINQKCSYKENQRSAARKDEERELEDAANVDLTSVDISRRDFQLWMRRLPLKLSSVTRREFINEEYLDCDSCDDLTDDKVKHLHSCDIYEIFDEKTSASFTDSKCLIC

>ERI-1/2

MSSQKSGIVVQSDETHILISSSHDIFVADPDQNTKLGDSVSFTTWDIANNLKRASDIKPRNQIVESVVYEQDQSLRLRLKILGFYDFTKSQQRVFRTDFITQVREGEKFKYLPKNAFFGRQITVKLTHQKNSIVWELDEFEEPKEKTYSLIGQIYYTDFSDAYVYVPGYPEPFVISKETNFSRSEEGTWVKFLACTATASVKKSEGFQFLEKDLLDTNRNREIAIKCSRKDGIIQSHHFEKVIDKDNRLADYGVLSDFENVLIYVKVSMEGVSKKPSLILGQFQTSFNVNPTLTRDQKEALDPPKRILKKQSSLPREGSSRKQVTQIQNLSRKIETGPRINFLPNFNNQDHGSEFFSDNEEMDLVPPLENSHLRVDPVPVPIEPSIQQNRTTTTAPISAPARRISSNESRRENPNSQPAPSKMQNVFREDPEAEYKRPERSNADNDETSSTISACSSKSRIVRENPDANYERPARRLPTPPLEANDNLEHQSHDSSSTDGLRDTSPVGNNTNPRSGSPSCHSDTHSSVSSSLWITTINSSKDLEFVKNEYIKLWIEDQASQKEMKNLKAKRIELKMKQKKLKAELNDEKMKNQKLEEAAFNDRKEKAELKKKVSNLEQSAQNTQRTLQNQTRELNLYSTNRSWLRKEFNSTNLLEHLRRNYHDLSKRLEQFAFN

>ERI-3

MQPVLVNSRPLRVKSHESESKLNLIEQEDQFEGANYSSSSGVIICYSNGTGEVITQEAFDDSGIHFIFSKATCIQYPSNFDPIGVGSVVQIFWSRSFERVVRGNHIIVQIEKMEVYKCCAMLREQVFVTFNSPSTAGVAIGVTERNITVAFHPNCSPVIRYETLKAHSIGRTEFEIKDRHRENTNRMVDVILAAVPFRVEIHGNVDKIPFFVIEKCRNSPGRSGAAVITKIMKNHFMEANFLQNSESIYFDSTSCHSNILEKVSIGSLINVLADPTFATSSYKWYGYDVTLCNNYLAHASTQRSFVLENNEILQNCKKLEKSPEEAETTTKNDLRFVPPQPEKGEVKKKKMTNCLKFNSKSAQFKLRHLILDRCFSELPEREAKSIINSYFIDRLAEGIKIEKIDKNWRTFGEILPKTPKKYSESLKKSIQNVLEPFGLNKPEKAAETPKIVEYFPKNPKKRVEIVEKPTVDEIRELFGALMDAEGFALNQRVKPHFVLPDTRWKPTERRYIGIYDDVQWTFMSTFCPKIEENSENRPLAGGWWYRRTVPRDHPVEIVQKMETRRNIIKDCTESPFIE

>ERI-5, isoform a

MLSEEPYETYHPEFPDPEIRRIALKSSAVVELLRIESPSSFFCRPVDHVTDDQLLIREPYRLTTRIIDFSAGNMAMAPLRPRVFARCLILKNLELIEAARIFFIDSAVTANVSWKCLFQIDENLKFHPWQAMHCTLGRLVHLSDSWTDTQCTEFRNIVSKFAKFQITANQCDVDFRSDRPSLLVNLYGLPNGTEIDKKVAIEEICAVSMQNVMVSQFPTNFMVNPKLEELDKEQDHLDVILLEEFRRDLPADWAHEPPADYREDDADWDILQCHVAEWNDTALEQFRRADGSFWAMLEPSCTVSPWEMHVTPILAPEKMSDNEHWIFEQLVKNSENQQKIDDFYSNLKNQRPLEMEEIKFALQTGRTYVMATIKNRQKSSAQWLRCEIIDFLPNANVALRYVDLGTRGILKLKNLHRMHIEHTKIAPACIEIGRFLDDDLSMADSEMEWNTHFWREIVPYDVPIVVGPDMEFLETGKLQFSQIRVAGDEDDENLLDKIPSPSPFFTERSDDLRTQKEDDDDGNVSDDKDSG

>GFL-1

MTEIVERMKKKNIVKPIVYGNTATPFGYKRDSDQHTHQWTVFLKPYLIEDPTKWIRKVQFKLHESYAVPYRVVEKPPYEVTETGWGEFEIQIRIYFVDPNEKPITAFHYLRLFQPTIELPSGNQIVCMEFYDEIIFQEPTVQMYKALQASDGKRPDKQAFLNDIEQVKTRTRELGEVAQKEIAAEIEDLRESLKDAHKMIVKYNTELAEQE

>LIN-15

BMQTLKTARLTSNPASIPTSSSSSAISAAAIQKTLDAVNRPPAVRASGILRHRTLPAPTQETAHHLDADPKTTELMARFFISQGIPFECAHEPAFLELMKHVDPNCVIPPTNVTKKLVDKISTSSKPQVNYTKTVGPLSVTIDICGDEDEKYLAFSIHYFEDLYERKNAIYLRKLLLTELDSNSLLTNIRRSVNSYSFSNVKFTNIVCPNEEICKLVEESAVVKRYNVCFYNYVTRFVADLMEIEEFSSGLTQLRTFVRYMKQNSDMYSKFRRMQLQKNAELDIPSIDSGDWHSTAIFLTRCLVWHDTFTEFCGKLDILHYIDNETFNHLIYLQRLLQQCMKHCRELSIPNNSISQVVPAIMSIRNFIASNSMGYRFQKRIRDSFTTSFKEITSGPSQDRYDIATLLDPRFAYRDTVYTAQTWRSLEKKVIDDFVNSDLQNDKNFYQDISILNQEQRYDIIKKEFAYYRQTSFVERPEENENSNHWWGMRQTDMEFLAVIAREYLASPAVSIDAGYYFGNGGKFQHICHTYSHQRLENCLALAGNYQTFRGKGASVDVISQSMIETLNNTASRLQKQVHLGLYAHGVDNISSDRDVQSIVGHHYPPMPTVANYDIPHVPKEEEKPPVANLQSTSSPATSSPTIIRPRAAPPPRTLAQGRPIPLNGKELKAVPIRQIPLQVRPLPPRPANVPIVPRPTVPQQFIKAPAPKPITLQAVVCSIPEKEIKKETEDVALLEKIKDEPLDEDDFNHPSTDPVPNRTTASSQGPSSYPRKIVVLASKLPTSQSSSPSTATSAQARSHVTTAQLIRCGPSEGTVPQKIHSHNFVQKFAQKQNFVHKYALNSQDHTGRLNQTVPMRAALRLPNSEQKSGAPSSINGKVQRDDFKLEPLDDFNGEPDYDNLIGAQRLMYSDNLNDASAEDAFARHRVTMEFQKRRACNRRCAVCGHLEIHERLKNVTIENEKLLIMLGCIYRGEFTLGQAQLFMARESKTYICRLHFLETLDEIYQMLRLKSADDILICPLDLIQNALITVSALRPHIIASQLRKILHDFAERNNHLRETPAELKKLGQQYFDYREPEPEPERNDVDEQEIIPKLFRQPRKQVLEADQHDGTVKVIEQEDFKLPTVKPSENEECDNPGVCCFCSKRGDRGGMLRVPRSEERLARWVDKLGPEFEARLHTNTENLICRSHFPDAAFSSRGRLLKGMIPDAAPEKVETTYIIQGNNFLKLKERKSGTDKNSAIDLANMLNPDGVEYTQEEEEEEEYEEMSRSPTEETSDDEPSQAAVYNNAPVIKRTYRKRELSNEDGPLNLVTPPAHTPNPRGRPRKYPKNSVTPEAEKSLTDYDYNPGTSQRRALKKGYVQLEDGEIVGEDCEYVPEKTPSGRLIRQAVARRSFAFADEEEEEEEYEESPIVKKPKIAGRPVGRPRKDANKLPTPTPPSNE

>MES-2

MSNSEPSTSTPSGKTKKRGKKCETSMGKSKKSKNLPRFVKIQPIFSSEKIKETVCEQGIEECKRMLKGHFNAIKDDYDIRVKDELDTDIKDWLKDASSSVNEYRRRLQENLGEGRTIAKFSFKNCEKYEENDYKVSDSTVTWIKPDRTEEGDLMKKFRAPCSRIEVGDISPPMIYWVPIEQSVATPDQLRLTHMPYFGDGIDDGNIYEHLIDMFPDGIHGFSDNWSYVNDWILYKLCRAALKDYQGSPDVFYYTLYRLWPNKSSQREFSSAFPVLCENFAEKGFDPSSLEPWKKTKIAEGAQNLRNPTCYACLAYTCAIHGFKAEIPIEFPNGEFYNAMLPLPNNPENDGKMCSGNCWKSVTMKEVSEVLVPDSEEILQKEVKIYFMKSRIAKMPIEDGALIVNIYVFNTYIPFCEFVKKYVDEDDEESKIRSCRDAYHLMMSMAENVSARRLKMGQPSNRLSIKDRVNNFRRNQLSQEKAKRKLRHDSLRIQALRDGLDAEKLIREDDMRDSQRNSEKVRMTAVTPITACRHAGPCNATAENCACRENGVCSYMCKCDINCSQRFPGCNCAAGQCYTKACQCYRANWECNPMTCNMCKCDAIDSNIIKCRNFGMTRMIQKRTYCGPSKIAGNGLFLLEPAEKDEFITEYTGERISDDEAERRGAIYDRYQCSYIFNIETGGAIDSYKIGNLARFANHDSKNPTCYARTMVVAGEHRIGFYAKRRLEISEELTFDYSYSGEHQIAFRMVQTKERSEKPSRPKSQKLSKPMTSE

>MES-3, isoform a

MTPATAEVKVGGRKRKNDEPVFIKTKQSRSARREEEKENLLNKSLPSTPTSSEAGSSRESSNPVTSSSRRKNPPTKLENIQKTLPTCSDGLEIRNYVKKYGLPEDNKFLVRNVFDKQLLFGKKYVCRRRVIKSIDEFFPRLKTDAHRENGLYNFCTLQFMKISSWGSSMDEKIYVTSAAIVQSYVIIVDDNDSVTCIENGISVIPMANRTLPSISYNSSNVKNITLTGPELKKAKNVYLAVIVKRKTPKLGENRVGTRGKSTRASFETAASSENFQQLVRFGCTLMYDKDAEINCLNDGVQRIILLDREEKPAYLKIFGKADEPELENAWMNNVKHLEFISDTNYNKEDSHLARLIFSSNTHNYFDRPDISKFSPWQPRSRRTTLKSGISVEPGTSCDEDCGSQYEDTITLKQLLLGTKKLCGRRKRHLTRLTSRWPCFSVDPTKINSYFNETGVTLLENSCSYRPGTQRSIPIKPTRVIRVREETPVSVPIEVITIEDDSDDSPCFSARNRAPGGTRKYGLDFIQSSGHMPYIRYVYMNRANDLVPQLNGEENGKIEGLMEAYPMNNAHMYSDIEFLRKEKLNLPKNAQKYGEMTILDYPLTKTETDEYNKKMSVMNVKDFAVKPRKPIPTVGNTTLSLDLEGCPISTFSEIFFPSGKTELFNILCRHFYSEAGQRPGCFSAQWKSRVLTGFINKYHQYIFDMKLNSLFEKQINLIAMVSPDWKLEDFELPMVRYKQLKKEWKNRQRDIPSSN

>MES-6

MEHTKKFKSLNLHGFDRSTEDYGKRPFVLTAKLLEDQKKAIYGCAFNQYAGIDEEQAVATVGGSFLHMYSVPIDINNIELQWSCNFPTDKSSKVEREESLFTVTWCYDTYEAENDRNPFKVVTGGTLGHIYVIDYVSRKLSNRLRSVGWEINDIRTCPANSNLIVCASSDQSIRIHHIRNEACLIVIGGLECHAGTILSVDWSTDGDFILSCGFDHQLMEWDLSVKQVKEHLERACKALHQDKINVLTQSQDIPYVSKGTMRKSAVSRNIPDKEEDQLLELHRELIPRPSCLLPIYTPSSVSTDMHSDYVDCIRFLIGTNYALSKGCGNEKAIHFWRFGPPKGEVENRIHGNVLRPKSCTTKFRTMNVPSGSAWFIKFAVDPRRRWLVCGGAGGSVMFFDLRNNEETNPTHTCSVGSRTVRQASFSTCGRFLVLVTDEGFVCRFDRVSASVDAKDLAKF

>MUT-2, isoform a

MSQPNKDQRSPEDIDFKVKANPKAFHKFNGKFQRVLRDHEDDFNILSISMQDHFDTTKQPKEEFGKKMDWCYQLKNIISKNNPTWLFNIVPTGSTVTGLATKNSDLDVAIHIPQAARVLEQEERGRNITDDERQASWREIQLEILQIVRLNLQNDEQINSRINWEHGIQLVQAQIQILKVMTVDGIDCDISVVMDRFLSSMHNSFLIRHLAHIDGRFAPLCAIVKQWAASTKVKDPKDGGFNSYALVLLVIHFLQCGTFPPILPNLQEIFKKDNFIAWDDKVYPSILNFGAPLPKPLPRIAPNNAPLARLFIEFLYYYSMFNFKENYIGARPVMVMDRRTSQNNMVRSSTNKEVCIQDPFDEHNPGRTVRTLNRIKDVMRSTYQKFLPVEGSEFTFPTLDDIINMSPEVPKPSRAEVRNFRAEATEVNRVGLGVYCRNSFV

>MUT-7

MEEEPYKRKLTKAEKKAKYRTDYAEPLKSRREVLKAIMNGPESERERKVRAKNREFFNEDYRSGVNIYGMAVDMMKAMPDRGKTSGQSLAVWYLEDFGVWLKESGQETELRQKYLTGTIQINALDVCTIGQKQLLSEIFDITKEKFTEDITQLLDAAIKKQDFSVAADMAIQYNLLRDHHFEHLVLPLMLSGKDQTAYKLISNNERMQQQLVEFFDRMVGISVVAVEEMLKPYKETKIMTIPMEKLTGKTLDKLISTIINKNTHEYNFSRELSKFAKNHSQNGNLKALKFNISERYEKGKSDDNYFQHMVETFTKAEDVREPILFYLWSSNDTEKQIDAICFAIYLGIASSSSYQLPNVMRDFFRQPDSKLREAKELLVRRKTLQVPLNGEQLFVFENERRTQIHMVKTESEMNYLCSEIKSLSDEPAPVYVGFDSEWKPSNLTAVHDSKIAIIQLFFKNCVWLVDCVELEKANMADDWWQKFASRLFGDSPVKVVGFDMRNDLDAMATIPALKSSMKIEDTKNAFDLKRLAENVCDIDMEILELPKKTFKLADLTHYLLGLELDKTEQCSNWQCRPLRKKQIVYAALDAVVVVETFKKILSIVEEKNKDADIEKIVRESNVMAPKKDKGHKSYRKLKTIPWLELYDILRSHRNPTRSPQRPHDIKVIVDTMLIGFGKNLRRVGIDVILPKDVSDFRKYLKEIERVGGEHLRHIITVPSKSYEALKMDYDNYTIAIPELNNMSPVDQLIEFFDLFNVDIRPEDVYPRCTECNSRLQIKFPGPVLHFLHQYCVIHVQNVYRADMSEFPLEEWWNRMLHINPDDYDGVKVEMSRPSPTSKWIVATVPTGCLHITRQTALHTNLPDGIEVRIHKVPDDEFKRRNLSFYVCGECGTVACDGRGNQASESTSQEC

>MUT-16, isoform a

MSESDDDYPELDISDQYIDPLGIVVGPPPASYTETDREETPMQNRTEDDTNSYGNSSGEHDYDSYLDSGDDDFDVDAYYANDNMDEPPPETIPDNLIQNVIGRNDNADYDFSDASNPEIMKRDLQLFLSSSLLSNPLKFRSGYSSDELDDCLKSCMGYSLQVTAVLLLPPEIISQLPNDSKTEHAHALVRGGWLQSKEGAFFPVISDSERETVVSLMNGSEEQHKRQERKKKEADTFESEEKEIRTLLTFNMIAELLMAVRNEYSIRSVKYQILSTAYTNMVTGAAHANIFRKYKDILQLDPEKLWNNDWFKEYTNRGTLKKFLTTARFSEIVVSQANGKTVELYFRADDEGNRPVVLFTDEHIADVRNKWKTGNQRNQNYGSQGNYRAGGQRSDDRRGPQQRRNVIVPDPNYQPSTFAGGISNNADDDGSLQPTTSSHFNRNTDRSTSRPPRAPTSPVNRVMETDPLMGQGTSSGAPQRSAIPNPFGGAPALSRSTITNGNRGPSYGDRGERVQDVGDTTSDSEITSEGSYSDEDPEQKEIKRQRRKDKLKKKQERELRSREKHTKSKQQPPSKIETRFNTYKKKSESSATDTSNTPPVDTVNVALPTPVVESSSTTAAPSIPVSTRPEVVVPPENPAPLREVGNFYSKSNHDEDRRNVQLPFTPADTHKPIKVAPKEPVRNPLLKERPSANGFINRRLPSHPAPPPVNQSQPANQPMQTAVYQNSHPGAPYIPQQPTYQPQLPVQQPQPHQYAPQPIHHQQPIHQPMHGQQYPPVNQQQPIYQQPAPQYPPYNSIQNNPQHGPSPFNYSQVPQPAYNHVGQQPSHMSNQPHINQNGYQNSYNPNQGPTSSDPNYGCNPQFNHYGSRSVYHEDHSSQRRRSPDQFPPNPPEYDPHGNFKLADYERDRMTVGYSQNPHQFDHHGSHMPHQSQPQGYDNFNGNSAPYFNKNGGQSNHQPEAQRSFSVLSSNRQPSNRELIFQDGIEKELRDIILRYRSMNLTVLTVQELRTEVSRRPAIPRYIDIVQYIRDSSSVAIVERGDIEPYVVLKDDIRN

>NRDE-3

MDLLDKVMGEMGSKPGSTAKKPATSASSTPRTNVWGTAKKPSSQQQPPKPLFTTPGSQQGSLGGRIPKREHTDRTGPDPKRKPLGGLSVPDSFNNFGTFRVQMNAWNLDISKMDERISRIMFRATLVHTDGRRFELSLGVSAFSGDVNRQQRRQAQCLLFRAWFKRNPELFKGMTDPAIAAYDAAETIYVGCSFFDVELTEHVCHLTEADFSPQEWKIVSLISRRSGSTFEIRIKTNPPIYTRGPNALTLENRSELTRIIEAITDQCLHNEKFLLYSSGTFPTKGGDIASPDEVTLIKSGFVKTTKIVDRDGVPDAIMTVDTTKSPFYKDTSLLKFFTAKMDQLTNSGGGPRGHNGGRERRDGGGNSRKYDDRRSPRDGEIDYDERTVSHYQRQFQDERISDGMLNTLKQSLKGLDCQPIHLKDSKANRSIMIDEIHTGTADSVTFEQKLPDGEMKLTSITEYYLQRYNYRLKFPHLPLVTSKRAKCYDFYPMELMSILPGQRIKQSHMTVDIQSYMTGKMSSLPDQHIKQSKLVLTEYLKLGDQPANRQMDAFRVSLKSIQPIVTNAHWLSPPDMKFANNQLYSLNPTRGVRFQTNGKFVMPARVKSVTIINYDKEFNRNVDMFAEGLAKHCSEQGMKFDSRPNSWKKVNLGSSDRRGTKVEIEEAIRNGVTIVFGIIAEKRPDMHDILKYFEEKLGQQTIQISSETADKFMRDHGGKQTIDNVIRKLNPKCGGTNFLIDVPESVGHRVVCNNSAEMRAKLYAKTQFIGFEMSHTGARTRFDIQKVMFDGDPTVVGVAYSLKHSAQLGGFSYFQESRLHKLTNLQEKMQICLNAYEQSSSYLPETVVVYRVGSGEGDYPQIVNEVNEMKLAARKKKHGYNPKFLVICTQRNSHIRVFPEHINERGKSMEQNVKSGTCVDVPGASHGYEEFILCCQTPLIGTVKPTKYTIIVNDCRWSKNEIMNVTYHLAFAHQVSYAPPAIPNVSYAAQNLAKRGHNNYKTHTKLVDMNDYSYRIKEKHEEIISSEEVDDILMRDFIETVSNDLNAMTINGRNFWA

>PASH-1, isoform a

MEQESGGKKSNEQLLSEREAIMRQLAMLGSGPEDLDVDDEEEDEEEEVEKMEEGVEETMENASVVKETTPVKDENIVEDSNSSEELFPYLDSPPEDKIQEISANDTEIDQIDSPIRFEGVKNPAQKMDVDGEEGTSTDARPVSGSSGSSVGEQIRAPVPVLKTIMDRIDLDKSNPLPEGWTVISHQSGMPVYYHKFTRVVTHSKPYLVEGIVRDHEIPVSSIPCLYKKIMDELHENVEKQSSKCPMTYEESQSMLEIPVKELRMSPDRYQKYCEKRFKFKQITVHRYINPAEKEGVVLKKRMNTILKKRGFDADYDQLKKNNKPGDVLLSSSTGAILIDLTPCPTNINKRSGSKKPYLLNPMGKTTVAVLNEFVQRLAKGTLLYEIEDTRNIHCPYKATALLTMKMCTLREMAGQCKESLVVLSEIAANDENSTTYSQGLLPDLRRFPVGSGVGANKKTARLVAARDALLKLIPKLRVSEDNVCDGMVEEDGTQQGFEELFKKVKIDSPNLVQMCTQCAIPKPYNLLRDAVSRSLRWNGMELVMKKEMIGNGSQLSKVILILGDIQEEAEAVGVKQATQMASQRLFKKMHPELLTYGSFLEIYGRLDDKSKIDNAKKQHDEVVRLPDTGNLLAPNFIVLSKLSEEMKNISLVYPPRKFLYGLATNSTGIKHDIRNVLTQTLMATLPPPPPQFFPMIGGPPLMHPTFSTSAPPPPPPPQPMEYGYNPMKQMPSRKRGRHDDSSSPSHQKPHK

>PPW-1, isoform d

MNIIVSGPAFENKNVITVGACVHYLIDPTVVDVAYKEYAEGQLYSGVGASKSVKTLEGTDKKVPSLFMTTEMKTTLFHPDYAPLVELLQTFRGFSTTLKANSPAAQRIEKAFVGLDVVLNYGVHKGLGEDGVVMKIRRFHTSAKETCFEVEKSTREFTNVFDYFKKKYGITLKYPDLFTIEAKGKQGKIHFPAEVLLLCPNQTVTNDQMINNEQADMIKMSAAQPHIRKTTTDTIVRNVGLASNNIYGFIKVEDPVNLEGMVLPKPKIAFAGNQLADLANPKSRFPTDFNRAGQYYDAKELTKWELVFVQNEEVQGLAKQLADEMVNNGMKCSNPTMSFIIRGDLEPIFKKAKAAGTQLLFFVVKSRYNYHQQIKALEQKYDVLTQEIRAETAEKVFRQPQTRLNIINKTNMKLGGLNYAIGSEAFNKPNRLIVGFVTSQRVGGNPDYPISVGFAANMLKHHQKFAGGYVYVHRDRDVFGSIIKDTLLAIFKTCTEQRGRPDDILLYFNGVSEGQFSMINEEFSARVKEACMAFQKEGTPPFRPHITIIASSKAHNERLYKSDKGRIVNLEPGTVVDHTIVSNVYTEWYHASAVARQGTAKATKFTLIFTTKAGPQAEPLWHLEQLTNDLCYDHQIVFHPVGLPVPLYIADRYSQRGAMVLAANQGPIYNEGQIDLAATNSAYGYGEKKLFTTRFNA

>PPW-2

MPATPVPPVTMPPVPPVGFPPVTAPPGLHPPPPVPPVPVPTLPVTSEHKTAHDACIKRLEQLEIPPDPKIYPSPTEPGTFGSVTEVLTNVFGVEVHKTSNFYQYSVHITAELSSKKEVTFTKKGKEDFIVHDRHEKCSAILFHALNRFEDFFKSSENCIIYDGQSILFTSMNLFEGIPTGKIKTKVFQINGADTNLKDLMVLPCIKLEVFPTKNPVVNFSLEDIGRRTSDSNIESVNMAYKQILDLAMSQYAIRETSKCVVFEHGKMFFINPLQEGYAPCDIVEVGDGKQMKPGLKKTLQFIEGPYGRNQNNPSIVIDGMKVAFHMEQLVFQKLSSITSNLANGITGSERERCAAVIKGLDCHSNYRGRTRHHKIESIHHEGAATARFELEGGGTCTVATYFKDKYKIQLRYPNANLIVCKERGALNFYPMELITISPNQRVRITQQTSSQSQRTTKESAVLPDIRQRLIMTGKIAAKITAENEVLGKMGVTVCDEPLVVKGRNLPAIRLASFETGEHLINPRDCKWRPQRYNRSAVAPKVWALYGVGSPGSQMNRDVMRRFCDEFMNMSRSKGILFPPPGDVNLLTPDAIENRLREAANAGCTFVLCITEDNITCLHQKYKFIEHHTQMIVQDMKLSKALSVVNASKKLTLENVINKTNVKLGGSNYVYLDTKNFLQEHLIIGVGISSPPPGTKYIMESRGILNPTIVGFAYNGNGKQEFSGDFVLNAAGQETIAPIEDIVSYSIKGYKKFHDGKAPKRITIYRSGSSEGNHGPIISYEVPLARVAMRNFSPDTQLLYIVVSKEHTYRFFKKESGGSSSGGSNSAGASNSGTLTSAPPKPWELNIGPGLTVDYGVTNPACKQFFLNSHMTLQGSAKTPLYTVLADDRNIGMSALEEFTFNLCHLHQIVGLPTSIPTPLYVANEYAKRGRNLWNEAINANEIPEVTGPESARLKKMTDGISYMGSGDMIDRRVNA

>PRG-1

MASGSGRGRGRGSGSNNSGGKDQKYLGTIQPDLFIRQQGQSKTGSSGQPQKCFANFIPIEMTQSDYSIYQYHVEFEPTVDSKANREKMLRDNNVTDEIGHHFVFDGMILYLKEEWEQNQMIEVQHPIDRSLICIRFKQTNRFLVDDPQTINIFNTIIRRSFDALQLTQLGRNYFNWGDSRAVPDYNMSILPGYETAIRMYEENFMLCVENRFKMVREESMYILFHKELRSCQNNPQRVQEKMNEMYGGTTIITRYNNKLHRYTRLDYSISPLSEFVKDGQSITLKEYFKNQYGIEITVDDQPIIISEGKPKQPGEPPQVSYIVPELCFPTGLTDEMRKDFKMMKEIAKHTRMSPQQRLVESRKLIVDLSKNEKVMECFKYWGISLGQDLANVQARVLKSEPLQGKKTYEGKQAEWARGVKECGIYRGSNMTNWIVIGPGSGNSGLLSQKFIEEARRLGKILQVQLGEPMCVPIRGISPNDYLEGVKGAIKQVDGEDIHMLVVMLADDNKTRYDSLKKFLCVECPIPNQCVNLRTLAGKSKDGGENKNLGSIVLKIVLQMICKTGGALWKVNIPLKNTMIVGYDLYHDSTLKGKTVGACVSTTSNDFTQFYSQTRPHENPTQLGNNLTHFVRKALKQYYDSNDQTLPSRLILYRDGAGDGQIPYIKNTEVKLVRDACDAVTDKAAELSNKVQEKIKLAFIIVTKRVNMRILKQGSSLDNAINPQPGTVVDTTVTRPERMDFYLVPQFVNQGTVTPVSYNIIHDDTDLGPDKHQQLAFKLCHLYYNWQGTVRVPAPCQYAHKLAFLTAQSLHDDANGCLRDKLFFL

>PRG-2, isoform a

MIQNDYSIYQYHVEFEPTVDSKATRENMLRQPSVTVEIGKHFVFDGMILYLKEEWDQNQMIEVQHPNDNSLICIRFKKTNRFLVDDPQTINIFNTIIRRSFDAMKLTQIGRNYFDWDNSRALRKELRSCQNNRQRVQEKMNEVYGGSTIITRYNNKLHRFTRLDNEITPLSKFQKDGEQIILKEYFKNQYDIDITDDEQFIIISEGKPKQPGEPPQVNYIVPELCFPTGLTDEMRKDFKMMKEIAKHTRMSPQQRLDETRKLITKLSQNQTMMECFQYWGISLGQDLANVQARVLKSEPLQGKRQYEGKQAEWARGVKECGIYRGSNMTNWIVIGPGSGNSGLLAQKFIAEARNLGRTLQVQLGEPMCVKINGISPNDYLEGLKAAIKSVDGEEIHMLVVMLADDNKTRYDSLKKYLCVECPIPNQCVNLRTLAGKSKDGGENKNLGSIVLKIVLQMICKTGGALWKVNIPLKSTMIVGYDLYHDSTLKGKTVGACVSTTSNDFTQFYSQTRPHENPTQLGNNLTHFVRKSLKQYYDNNDKTLPSRLILYRDGAGDGQIPYIKNTEVKLVRDACDAVTDKAAELSNKVQEKIKLAFIIVTKRVNMRILKQGSSSKSAINPQPGTVVDTTVTRPERMDFYLVPQFVNQGTVTPVSYNIIHDDTGLGPDKHQQLAFKLCHLYYNWQGTVRVPAPCQYAHKLAFLTAQSLHDDANGYLRDKLFFL

>RDE-1

MSSNFPELEKGFYRHSLDPEMKWLARPTGKCDGKFYEKKVLLLVNWFKFSSKIYDREYYEYEVKMTKEVLNRKPGKPFPKKTEIPIPDRAKLFWQHLRHEKKQTDFILEDYVFDEKDTVYSVCRLNTVTSKMLVSEKVVKKDSEKKDEKDLEKKILYTMILTYRKKFHLNFSRENPEKDEEANRSYKFLKNVMTQKVRYAPFVNEEIKVQFAKNFVYDNNSILRVPESFHDPNRFEQSLEVAPRIEAWFGIYIGIKELFDGEPVLNFAIVDKLFYNAPKMSLLDYLLLIVDPQSCNDDVRKDLKTKLMAGKMTIRQAARPRIRQLLENLKLKCAEVWDNEMSRLTERHLTFLDLCEENSLVYKVTGKSDRGRNAKKYDTTLFKIYEENKKFIEFPHLPLVKVKSGAKEYAVPMEHLEVHEKPQRYKNRIDLVMQDKFLKRATRKPHDYKENTLKMLKELDFSSEELNFVERFGLCSKLQMIECPGKVLKEPMLVNSVNEQIKMTPVIRGFQEKQLNVVPEKELCCAVFVVNETAGNPCLEENDVVKFYTELIGGCKFRGIRIGANENRGAQSIMYDATKNEYAFYKNCTLNTGIGRFEIAATEAKNMFERLPDKEQKVLMFIIISKRQLNAYGFVKHYCDHTIGVANQHITSETVTKALASLRHEKGSKRIFYQIALKINAKLGGINQELDWSEIAEISPEEKERRKTMPLTMYVGIDVTHPTSYSGIDYSIAAVVASINPGGTIYRNMIVTQEECRPGERAVAHGRERTDILEAKFVKLLREFAENNDNRAPAHIVVYRDGVSDSEMLRVSHDELRSLKSEVKQFMSERDGEDPEPKYTFIVIQKRHNTRLLRRMEKDKPVVNKDLTPAETDVAVAAVKQWEEDMKESKETGIVNPSSGTTVDKLIVSKYKFDFFLASHHGVLGTSRPGHYTVMYDDKGMSQDEVYKMTYGLAFLSARCRKPISLPVPVHYAHLSCEKAKELYRTYKEHYIGDYAQPRTRHEMEHFLQTNVKYPGMSFA

>RDE-2, isoform a

MHNGYHSYFPPNHYYAQSQPSSSYNPQPQIQQPQTQIYGLVVGYNKYGNRNYLFYTKNIDGFVEISGNIQSLQIGRWLKLTVCQNFVGQYVLDSSNSCGYENWDVLPNHRNSSARFFPKYEANDEGEILITARFDVIVDRKNNLYDFTSYDISYDHIIDDFSLIKSSCQHYDGRSFIVEAQASLAGWVALSVHEDPQKLHTCDAVSRNGYQSLPNPVVDSDVQYLHDTRLLDIMTGNGYVQTPETDVQDQQSSQHQEDVHSQMNSQTSDSYNSSRVVSENREIPPETFRSQSIELNQVDSSLSQTTISSRAAPVIDSNQLSDEDEIDDEDTYGTRGTSNIPMRPFIKDLAPTMLQLLRQDKTDSEKPQSALCTVVQKIDGFAILYTAKRDVINVLLQERSCEGLERSPQLGDVAFFDILPRRIETKDRLIFKIPYTHIAVKKKPDTPDSLLKIDCFKNSVRCFGGVLEMKVKIALSKPELVVEQYHDNTEMNSDHHFYYLKATNGVLVTIPKERLLNHLNSKLSADFDLIAWVVHRKPIGNVSLHIGKGGEAYQQFTNGDIRELPPLSSNQYFMNVRK

>RDE-4

MDLTKLTFESVFGGSDVPMKPSRSEDNKTPRNRTDLEMFLKKTPLMVLEEAAKAVYQKTPTWGTVELPEGFEMTLILNEITVKGQATSKKAARQKAAVEYLRKVVEKGKHEIFFIPGTTKEEALSNIDQISDKAEELKRSTSDAVQDNDNDDSIPTSAEFPPGISPTENWVGKLQEKSQKSKLQAPIYEDSKNERTERFLVICTMCNQKTRGIRSKKKDAKNLAAWLMWKALEDGIESLESYDMVDVIENLEEAEHLLEIQDQASKIKDKHSALIDILSDKKRFSDYSMDFNVLSVSTMGIHQVLLEISFRRLVSPDPDDLEMGAEHTQTEEIMKATAEKEKLRKKNMPDSGPLVFAGHGSSAEEAKQCACKSAIIHFNTYDFTD

>RHA-1

MSRDVKEFLYAWLGKNKYGNPTYDTKSETRSGRQRFKCELRITGFGYTAFGNSTNKKDAATNAAQDFCQYLVREGKMQQSDIPTLTSSSLEASSTWQDSETATMFCGGEDGNSFQESQQPIPQKRFPWSNNAYQRNEGTHEQYITQKAEEIAASETVDLKSEIHGGWTMENSKKALNEFLQKMRLPQVNYGTKIRESNTVKTMETTAQIFVPQINKNLVGKGTGSNKKVSEAACAMNIVRQMFHLNIMQSYSGPIKKSKVSTLPEIAISIPEDLSTRVTEYVRSCGLELPEINETSATPEAPTSLLTDVKLAQFPVSEICSASNISWAPPLQNWNPWRASNIDEEPLAFMSMEQISQRIMEKEDFKRGEALDKITAQRGELPVAQYRENIVQTVAENRVTLIKGETGCGKSTQVAQFLLESFLENSNGASFNAVVSQPRRISAISLAERVANERGEEVGETCGYNVRFDSATPRPYGSIMFCTVGVLLRMMENGLRGISHVIIDEIHERDVDTDFVLIVLREMISTYRDLRVVLMSATIDTDLFTNFFSSIPDVGPTPVITMHGRTFPVQSFYLEDILHNLQHMPEEPDQKKRKKGGPPPPDDDEGDEEVDDKGRNMNILTDPSINESLKTAMSRISEKDIPFGVIEAILNDIASRGVDGAVLVFLPGWAEIMTLCNRLLEHQEFGQANKYEILPLHSQLTSQEQRKVFNHYPGKRKIIVSTNIAETSITIDDVVYVIDSCKAKERMYTSNNNMVHFATVWASKTNVIQRRGRAGRVRAGYAFHLCSKMRFEALDDHGTAEMLRIPLHQIALTIKLLRLGSVGEFLGKALQPPPYDMVVESEAVLQAMGALDRNLELTSLGKMLARMPIEPVIAKVLILGTALGAGSVMCDVASAMSFPTPFVPREKHHSRLSGTQRKFAGNKFSDHVAIVSVIQGYREAVQMGASAAEREFCERYSLSNPVLKMTDGARRQLIDVLRNQCSFPEDILFDISVNVNGPDRELNLMRSLLVMALYPNVAYYVGKRKVLTIEQSSALINKYSMLVPMNNRQEMDFPSPLLVFTEKVRTRCISCKQMSVISAIQLLVFGSRKVECVGEGLVRIDETITIRMNVSTAAALIGLRPCIEALLVKSCENPESLAGLNPSDAELRQLLRDISSEEFMSEAGPIKDSLLTDNALIQKGPAPNRLEYADWGPSNNNSSFQNQDFPPAAGGKVHPYRGNRRGNHPYAQNRPYAPPNSGMGYQNFNNSGYGASGDWNAGRGNYGNVGSGYRGAGGGGGYRGGRGGRGGGRGGGGRGWNASNW

>RRF-1, isoform a

MSERSHGFIKFEFPEFDNSLQVIEGSIDTMLLTFTSVLRKYEIEIKSRQETQIVEEQDCDCFFEINFEVESEQFDHTIIDAMHDYLSDLNVYVPYQRPNIVLHGSDFWLRTLDCHTEIPLAAIYFGNIQGGTYFNHWQVSFSRENISSRDMLHKIHAEFEFDKTDMITVQFQCFEEKKQKFEDSRKQKVRVNYQLTIRRDSIRRIIVDPRVEGCNTCVHFEVNCPPLIRKGYIDNDKSSFHKPFYERQKRFDCDWRNGNVNHGNPQDAAIADSPFFTIEFHKEISTKEMYRVLSRLRSRTKVLIEFANLPSIDVPMGSHYPYNRWNLKKSPTDSNAPIFREFLKEIFPPKYEIVDDKLIDVNEERKFSITYLIECLLSRGAIVKDQLLLNEQHWKNFLEIIIWYYRNDNQLCEAALEDLVHLIDGRKRIGSILKCLDKICQKREVMKLVNGLTEKESIEGYQRVRKVIFTPTRVIYIAPETIMGNRVLRKFDKDGTRVLRVTFRDDNNKKMRSNVTGKLLDRTANKYLEHGVRIANREYGFLGCSNSQMRDNGAYFMMRFTDKQLDRFYKCNPTASNINFKPKIDEVRFQLGRFSEIENVPKLMARLGQCFTQSRLTGVGLGRDDYCSTYDLTGGRATNGSEYTFSDGVGMMSYQFAQEVSQAMQFGKAVPSCFQIRFRGNKGVIAIEPFLDEIRKWALVNGVTSMKMAKCLFRPSQIKFQAKAISGDQIEMVKFSSAVLVALNKPFINILDQVSEMQSLDCHKRITSRIEELMDRQILSFAKQMNEETFCRNKLKEFPRRIDIDNLRTMWGFTLSSEPFFRSLIKASIKFSITKQLCKEQIQIPSELGRSMLGVVDETGRLQYGQIFVQYTKNYKKKLPPRDSNNKVHGSEIVTGTVLLTKNPCIVPGDVRIFEAVDIPELHHMCDVVVFPQHGPRPHPDEMAGSDLDGDEYSVIWDQELLLERNEEPFDFAVEKIKVPYDREKLDVLMREFYVTYLKLDSVGQISNSHLHNSDQYGLNSRVCMDLAKKNCQAVDFTKSGQPPDPLETKWRADPVTFEVIPPENPERIPDFHMGNERSPMYVSPRLCGKLFREFQAIDNVIKISEERDEQYNIELDETIFVTGFERYMESAQKQLSSYNGQLRSIMENYGIRSEGEIMSGCIVEMRNRISDKDQDDMSFYNTNQMIETKMTSLVCKFRETFFEEFGGFTVKCTLLPNAYDNGNCLNYRCEDPDQEVRKKAVAWYRACYECAQSTREVRKLSFAWIAYDVIAKVKETNVLNNERMQIGGANPMYTFLEEHRKQYLIDHDADFKNFCELDHLITGEKSKEAISILKIYLEMIPGLDSVFFMLMRWGESLRLFDGKPIKIYHFFLMFILFATRQLASADGNAEPFFKIIEKEEYEKQKRDSSRGNIDPLTEKKRSDMMVKFFQFMGCRKFRKMSTLSFCPLNFSSIFMRGEWRIFHESALKTYYNILFNLRFEELPVSSDPTITAETMDRECEPFVIELPENINVNDLINNMKKHTNVSTVKMRRQEKNPINDKAKPKTTVRYIVSVSGTLESIQMLKKLSAVTIPIKSHWEGEEVSQQMASLCYQKVMNGEF

>RRF-3

MLPFDNDDSSDDATTSVRPKHPRGVPQSQSTFPRGRSNFSSGTLPNRKTECTPVNTLTIGHSNKMLLTTFRMDRNSKSKSEVDVQEQPVHSSSSAFPGNHLNNFSYPVNRGYLRDYLLQSQRPSTSKPVDCSVLKRHSLPSTHILYEKTKHRGGVNIEEQEKLVRMLWAAAEESETVAKTRQFSKKQAIELNFDAKLIGSMNNDCFGYCRAHMENIKDVLKTHLKLSKVDEVNWIKVGMVPRAAYEDKSYVIDAHLVLTPNGEVEDENELFSEFASSFTSRITGMLHDQVFLEVPKMHTLFTKITPQHMDINISAIAIGNCPNSGLFLVRGDFISQENTVCSVKLQSHHNADASRENSSFKVAGSNKYLSYARFEHDKRLAVVYFGVRLAEFADDGLDHAGFRLNLYYNLFVRIVVDMSHETTNSIYIQMKNPPHLWEGIPKNTIFHPSKSKVLNMETCTEWTRVLSWPGDAEGRGVGCTSEAFSQSSWIRLTMRKDDDNDSVSSTQLMDIVTRLSARSKAKVMFGSIFSIRRKLAPSPAFHSLGSFRANYALQALITRGSVFTDQLFDATDENIPSSDNDNDEDDDDDVDDTKKPMELVHEPLFLKLVRRGMKECSQATEETLEQLLNAFDERRQIDVVTAFTTMYQSRKIQYERLLKGESLQDVGLAKPLPKNCVSVAKVIVTPSRILLMAPEVMMVNRVVRRFGPDYALRCVFRDDNLGRLAIRDFSINNIDHMSNIVTEGIYLTLKNGIQVADRVYSFLGWSNSQMRDQGCYLYAPRVNALTGEVTGTVEDIRVWMGDFRDAISVPKMMSRMGQCFTQAQPTVRLERHHWIVEPDIEGGVENKYCFSDGCGRISIKLATHISKILQLKEVPACFQVRFKGFKGILVIDPTIDDIINMPKVIFRKSQQKFGEGGGELQDEYLEVVKYAMPSPVCLNRPFITILDQVSEKQSASSHRRITNRVHYYLERELCSLSNMLINENQAAEELVNRTNLAIDWNAASKRAGFELSVDPLIRDMLFSIYRYNIIHHISKAKIFLPPSLGRSMYGVVDETGLLQYGQVFIQYSPSIRQTSNRPILKTGKVLITKNPCHVPGDVRVFDAVWQPALAHLVDVVVFPQHGPRPHPDEMAGSDLDGDEYSIIWDQEMLLDYNEEAMVFPSSSAAEEDKEPTTDDMVEFFLRYLQQDSIGRMSHAHLAYADLHGLFHENCHAIALKCAVAVDFPKSGVPAEPLSSFEQCEMTPDYMMSGGKPMYYSTRLNGQLHRKARKVEEVLEEFETRGSVFEREYDKLICPEDVDVFFGNEIKLVQTLTLRDEYVDRMQQLLDEYGIEDEASVVSGHAASIKRLAGMERDDYSFYHTDKVVELRYEKLYAVFRAKFFEEFGGEEINIENDGKNTRLKCTKAMHEKIRQWYFVAYVQPKINKAGRCIGQSLPWVAWDALCDLRRQLMLDKNDAVLRGKYPIAARLEEEIENSIERQFDKFLKLKDLIESHKDALFLRRYVYFYGDQIIKMLFILKVWLERENVLPSSVLSIWQLGRLLIRLGLGDLLGNPTIDYEKSLLMPTTMFQQWISKKEDADEAPILRNFDMGTMMLEFLRYLASQSFASAESISLRVFYEKDIVEPILTKSAQWMPLHLIAYRTFHSIAVSGRFDALHLDDEDAVDQITESKDPILVNESLFSSRNYNDDYPISRSRILQSLKDWSGVKEIIPREITGTRKSDMIYVTSVGTVLARQRLARLLLLSGETIRDAIANNVVPNEVRDEFL

>RSD-2, isoform a

MSSCRVLILHHDQENDVFGCFKRESHEFFWARIEGQQQLEPRPGFFLTFIENEVMTEDGVMKIDASTSTIKEPEPADCVILPTHTNEQVVYYKSWIAFGDNPNYLTYNRDLGYADWYGIVDCSDSPPTGPGVYKAFIYVNDLNYQESGNPLFKVTGRLKFLANKDTSDTMMKLEIFEQNRLEELRLSPKKMSDSQVIAIILFDNEETNSAICYSEQLESSIKVHLPVGSPAKAGQLILFTEYNYENDTYFPDPRPDAIEYRECEMTRIENGELLISAKAACSSIETHITYPQSVAFADTFGKVNVAAESMERGVTYRVELAINIIDSEANNVPLFKISDIGEKLTGEENEAFLRKLNDFEELQGTWEDWTPEEIAVELEPVAAAEEPADPEVEEAKIEENGVNDESLTQSESDSVFLPQIESSASSEETMSESNSAENSEKPKKTFGRMPQSLSESSSSTISSSKVGVDHSVIVLNVDNDECVGFETTQLKLMTICTAGVTVPLRPYGIFQFIAAGNNYGKTSIQTQSARVSECEGAVFSSDDVPKISANVIFSSNPKHKSYNKEVGVSDRYGFVEIPRITGDFEFGTVYSTQITMVQYGDPLKACFKAVAPLKRQFIDDLDHYLQVMGKYEDNIYQLSRKNGLGAKSISRLPNEPRNGSKHGSTKRFTTPHVTGLVVDMEKRGNYFGCVWTCFGDVVFKSTPTFSQNGGYPSVGDLVNIRVEILSPGTKDQKNLGASMTEKPASLSTTVIWRNNGFIVKAYLIMKPEKLIYSEDNNRFYEHDQLGIVVADSSIYGSDNEQSKVFEFERLRNPITLGFTTNLMNKCYWAARLPMIHREPSPPEPINKYNDENVYDDFQKGGPQSVNSYRSDRQYQEKPPMQYQSYHDPADTISIHSQDNHRPMETRSNAGTSVSSVDRKEVGVVMKKVDKNTQIVYLSGQKAAAIHHWNTHSQSTIVGNSYIFMYRPIEVHKATHYAPFEITQVVEILNTENHGRVDRSEFSSLFTFDLCNVDQRYVERLAFGEALVLRSNDVGHVVMASNVKTPLNPSMTAVEVCQTYKGKRESENLVGYCRLVYGKVPREIVDGHFSDQFVSAYYFELHEILATSNAYIVRNENLDRRNLRGNTIEEVRDDISIQKRIAHMNFGQNEQQQQQSSLSSRGPTRPYSPVGSYQSSHQNFDNRSAAHNGDGRSTSQSHHSAGGSMNHQNFYQPPVYQSSSSQQSYQPRYNGAPSGPLPPPSSQSSFNGAPAPPHYSEMSNDYNRMVRKLAELTDTAAQMIINNDIRQQIKNISPDLLDDLERNIDACQREKRHYYEKYGN

>RSD-3

MSDLLAGITTSIKSTANAITKNEYVRKVTESMNDAIMNYPKAMMDVREATNEDPWGPTGPQMKKICEYTRSRYMEDFYNVYTPLFQRMLENNKDAWRRVYKSLILLDYLLKNGSERFVQEAREKAYELRRLESYKYIDEKGKDQGINIRHRVKQILEMMNDDELLQAERQKANSDDKSKYRGFDQYDMKMSGSSFKSSNSGYDNKWDKEDSFSSSVTKKYDDQYKSKEVSNFSFSSNVNRSPSPELGFVDDSKKKDDDDGFGDFVSSRSSQPAATNKSKPNPSKSFFDDPIPAIPPPSGAPSNYVAPPLSPVSKNAPRNMDLLFDVSAPTPAAPQNNGAPIDLFGKLDAPVVNAAPAVAPSNGGLDLFAFGAAPMAQSIPMATPVAAPINTASNGSFNGFDFGSSFGSTNNSTPSYGGAAPLQPMSGLSQPQAQPAQSNLGGPSPKIGNSFAGLDQFMSLSLGSTNSNAPKAKTLNQMMGGNN

>RSD-6

MNEKELADSVFGGLMAKAMGKMVHESEICPLVDQIEGKGQTNEELQGLRLSLSDLLLRHPDKFINCGNRNWRLNQNNHAFQQLAESISDKKSGKGGKRSARGGSNLPSFNRARSGGIPRNHNQTHGFRQSAPAHRGHHRPPAGNHGSSFRPNLNISQPNPSNDYSKMTGLHYPVSRNVLASNNGSNSRFSNQPPRREDHHEKMIMRTPYRDPSPRRGNNYGDIRGRPDSRDGYNGDRDSTRYSSPRRNEQPGRPSSRGSNISDYSQFSSDTNAESNTERRSRPDASSPDLADLARRFNSSSINVQPQPLESVASSDVLSKKIAKAVRKIMKKLNEGEKLSLENLFDEIERKNKILIPGKNGKEKEDFLKDLKKIMPKLLSALTIDTETGDLTWNEAAVTTDSPIHEYMEDDLHYYVVDYVKMLDKARFRVTDEAERLSNATGVRMERTCEKLTYVLFITFEGEYRYASGSKTEVLKISKITTVGDAPEFYQTSFEHKIFENVPANALVKCGIVRYQNVQKFAVRPEEYIKPYEKMNQEIQDFMSQIRNEKSVPFEMWRPNHACLVQLNDSTTILKWSRAMIIKIETQLIFLFILDSGFRKVVPSSDLRLMPQKFAKLPPFAIPCTLDESEDDKDNIEFGGKEYLSNGPGNLSVTCSGAATASDGILTYPIKLFSTCHGGPLTNIKDVFI

>SAGO-1

MSNITQVTSSMASASLSNKAPLPVGHQPLAEKKPKEVNQEGTPVQIVTNMRKINLEKNHSIFKYSVQVLFVYQKSDGTELVLEKSKSVGSGCDHERSKSHCLRVYRKAAKQCQELKSGGPFCYDSQGCLYSFSKLKNDEFSTNITGSDISNNPKFLRVEFKLAKVQESFQTTTNDVAKSVNCRPALQEKTILEAMNQIVSTAPINHPNVLTIGNCVHYLYDDTNIDIRSITGEGGKSSAVGASKSVRTLEGTGKTPCLYMATELKTTLFHPDNCSLLKVFMDYRGFNGSLKANSPFVLKNKNAFIGLWCYTTHGKCSDWKDDRPMIKIKDFGLSAKETTFERDNKKISVFNYFQVKYNMTLKYPDLFTVVARGKDGKNQHIPVECLDLCNSQTVRTEQMVGTEQADLIKLAAAKPHDRKKITDTVVNSIGLASEPKGIISVGAPESVTGLVLPKPDIYFSGGKKVFWNDPKKRGPATDFMPAGTFIKPTKLTNWEVVFDNGVQLVDCIQHLTSTMRQLGMEVSNPTVSLINRGYLRSIFENAKAANRQLIMFITKSMNNYHTEIKCLEQEFDLLTQDIRFETAVKLAQQQNTRKNIIYKTNMKLGGLNYELRSGVFSNSKRLIIGFETSQRGGLGDAPIAIGFAANMMSHSQQFAGGYMFVKKSADNYGPVIPEILLTILKQAKANRPNDRPDELLIYFSGVSEGQHALVNEYYANQVKAACGLFNESFRPHITLILASKVHNTRVYKSENGGGVCNVEPGTVIDHTIVSPVLSEWYHAGSLARQGTSKLVKYSLIFNTKKNEKLSVYERLTNELCYEMQIVFHPTSLPIPLHIAGTYSERGSQMLALKKPIYTNGEFNQVATNEQLGYASKKLFGTRFNA

>SAGO-2, isoform a

MEKQLKAMSVSDKPAAPAAQKLGTAPLAAKKTRNEEWGTKVNIDTNIRKLTIKPNQPIYKYAVQVNYVFRKPDGTEATIEMSKSAKKGTEHDNDKTRCQNVYNEAIKRYDELKTGGPFFYDRQASLYTLTKLKNESISFVVTDKICKRQNFKEAQFVLKKVDQSFQSTSNDVIKTTNSCPANADKTLLEAMNIIVSGPAFENKNVITVGACVHYLIDPTGVDVAYKEYPEGQLYSGVGVSKSVKTLEGTDKKVPSLFMTTEMKTTLFHPDYAPLVELLQTFRGFSTTLKANSPAAQRIEKAFVGLDVVLNYGVHKGLGEDGVVMKIRRFHTSAKETCFEVEKSTREFTNVFDYFKKKYGITLKYPDLFTIEAKGKQGKIHFPAEVLLLCPNQTVTNDQMINNEQADMIKMSAAQPHIRKTTTDTIVRNVGLASNNIYGFIKVEDPVNLEGMVLPKPKIAFAGNRLADLANPKSRFPTDFNRAGQYYDAKELTKWELVFVQNEEVQGLAKQLADEMVNNGMKCSNPTMSFIIRGDLEPIFKKAKAAGTQLLFFVVKSRYNYHQQIKALEQKYDVLTQEIRAETAEKVFRQPQTRLNIINKTNMKLGGLNYAIGSEAFNKPNRLIVGFVTSQRVGGNPDYPISVGFAANMLKHHQKFAGGYVYVHRDRDVFGSIIKDTLLTIFKTCTEQRGRPDDILLYFNGVSEGQFSMINEEFSARVKEACMAFQKEGTPPFRPHITIIASSKAHNERLYKSDKGRIVNLEPGTVVDHTIVSNVYTEWYHASAVARQGTAKATKFTLIFTTKAGPQAEPLWHLEQLTNDLCYDHQIVFHPVGLPVPLYIADRYSQRGAMVLAANQGPIYNEGQIDLAATNSAYGYGEKKLFTTRFNA

>SID-1

MIRVYLIILMHLVIGLTQNNSTTPSPIITSSNSSVLVFEISSKMKMIEKKLEANTVHVLRLELDQSFILDLTKVAAEIVDSSKYSKEDGVILEVTVSNGRDSFLLKLPTVYPNLKLYTDGKLLNPLVEQDFGAHRKRHRIGDPHFHQNLIVTVQSRLNADIDYRLHVTHLDRAQYDFLKFKTGQTTKTLSNQKLTFVKPIGFFLNCSEQNISQFHVTLYSEDDICANLITVPANESIYDRSVISDKTHNRRVLSFTKRADIFFTETEISMFKSFRIFVFIAPDDSGCSTNTSRKSFNEKKKISFEFKKLENQSYAVPTALMMIFLTTPCLLFLPIVINIIKNSRKLAPSQSNLISFSPVPSEQRDMDLSHDEQQNTSSELENNGEIPAAENQIVEEITAENQETSVEEGNREIQVKIPLKQDSLSLHGQMLQYPVAIILPVLMHTAIEFHKWTTSTMANRDEMCFHNHACARPLGELRAWNNIITNIGYTLYGAIFIVLSICRRGRHEYSHVFGTYECTLLDVTIGVFMVLQSIASATYHICPSDVAFQFDTPCIQVICGLLMVRQWFVRHESPSPAYTNILLVGVVSLNFLISAFSKTSYVRFIIAVIHVIVVGSICLAKERSLGSEKLKTRFFIMAFSMGNFAAIVMYLTLSAFHLNQIATYCFIINCIMYLMYYGCMKVLHSERITSKAKLCGALSLLAWAVAGFFFFQDDTDWTRSAAASRALNKPCLLLGFFGSHDLWHIFGALAGLFTFIFVSFVDDDLINTRKTSINIF

>SID-2

MPRFVYFCFALIALLPISWTMDGILITDVEIHVDVCQISCKASNTASLLINDAPFTPMCNSAGDQIFFTYNGTAAISDLKNVTFILEVTTDTKNCTFTANYTGYFTPDPKSKPFQLGFASATLNRDMGKVTKTIMEDSGEMVEQDFSNSSAVPTPASTTPLPQSTVAHLTIAYVHLQYEETKTVVNKNGGAVAVAVIEGIALIAILAFLGYRTMVNHKLQNSTRTNGLYGYDNNNSSRITVPDAMRMSDIPPPRDPMYASPPTPLSQPTPARNTVMTTQELVVPTANSSAAQPSTTSNGQFNDPFATLESW

>SID-3, isoform a

MASTSGALVDDNVLEVLRKAQLDAFISQFVFLFNVRRFDHFSHVRDKDMLEIGMQQVQIRQLREQILKMSREMWNRSDPKQVYIQADQSMPAQNSIDEKALIPNEQIKLYELIGEGSFAVVKRGTWTQSNGTHVNVAVKILRDISPNIMDDLRVEASHLLKLQHPSLIRLYGIVRQPAMMVFELCEGGSLLDRLRDDKKAILLVSRLHDYCMQIAKALQFLESKHCVHRDVAARNILLARDERTVKICDFGLMRALKENEQMYTMAPQKKVPFAWCPPEALRHRKFSHASDVWSYGVTIWEVFTFGEEPWVGCRAIDVLKNIDAGERLEKPKYCSERIYQIMKNCWKFNPAERCKFGAIREDLVAAMFLDAVARETYNSIQPGALQLTKGDEVVVVENTGQDWFGQNKKNQKFGTFPRSVVFAQTNNAVAAATAVTPQKVPTAPTIRIPPSHPPPAPLKPLNNNTKTSLNDRTSKISMPVAGSFIHTGHGDPLGGQSWGNPATIADMYLKNPVNGAPLSSMSSGAEIIASKELLTNGGRSTHQPAAPSPAVMSKIRGLSLDLPEYDDFDRAFDDGFSPSKIELPREFCGNDSVISGGSNSIGLANTYVMEPPKQAFDIRGNRVLPPTNKAPVLIPTNPAPSVISSTASAGITLSTNSSQMFTSQDRHSNMPANLFPELQHRLNQGSSTGNGVRPRPASSIGIQNNDLSMLNPQVNRPFSVVNVPIVQQPANIPCLVPTPAPPAPAHFSQPVSSQRVAQQQQNTLQKALNDELKGNLNKRPTGTTAPPSNGFNAPRADVAPVQQRPISSASIPALQPQPIQHIQKPIQPQQVRIPPSTAPVQKPVQVSAPTHSNVAPTTSSQASADARNPLPPKTSPPVSNTPITVAPVHAAPTTSAPSTSVVTRRPTSTTAQMSDEERRSRIAMDISSALPAPSALLYGSNSTSSLPSAAVSTASSVPSTARDNPVETRPSQPHVTMPPKKSSEPILSSEVLQPTRLPSATTSQAKPVTQPIRHPSPPVATVIPTAVVDKKPVSQNQGSNVPLFNITNSSNGYPQLNGYPNYGNGFQAYGYGMNYHQGYPGYQGYNSYGNGMGQLALTHNAVTSLPPLVPSENRFSGTAQPLGESDIMEFLGTQQRQAGSSSRAVPPASASTSAASGITDLSMADKMEVLYREADFTHKGNCDTMVSQCNGNTEQALKLLKQQHLVDMELAMSTETARQALEARQYDLPAAANMLLG

>SID-5

MPSKNCAKNLHACQWERDIALVFLGLMVLFNIGQVVYMNRARLYRLIRRGAEQIPADDEEPIIGIRD

>SMG-2

MDDSDDEYSRSHGETLTFVDPEDDGVSIGNTQDSQFAYEQFSVPTQSSQATDLLPGGTDGTTNDLPFHDVEDDESDSEKSLTEEQQQQKLPEHACRYCGISDPLCVAKCTVCRKWFCNSNDGTSGGHIVHHMVRSQHKEAYTHKDSPCGDTQLECYRCGSKNVFNLGFIPGKKDQVVVIICRTPCASIAFQNDDNWSPEDWKSVIAEKQLLSWIVNVPSEEQVARARKITATQAVRMEELWRDHPEATVDDLNKPGLDREPDHVQLRYVDAHHYSKVFRPLVAIEAEYDRRVKESASQAVGTVRWEQGLRQSVLAFFHLPQFADGVMKLAKGDELRLKHSQTVDGSEWTKIGSVFKIPDNHGDEVGIEIRGAVDKSVMESRIMFTVDVVWNATTFERQYKALAALLNDSKAISPYLYQKLLGHPAEEMMLKFDLPRRLSVAGLPELNSSQMQAVKQVLTRPLSLIQGPPGTGKTVVSATIVYHLVQKTEGNVLVCSPSNIAVDHLAEKIHKTGLKVVRLCARSREHSETTVPYLTLQHQLKVMGGAELQKLIQLKDEAGELEFKDDLRYMQLKRVKEHELLAAADVICCTCSSAADARLSKIRTRTVLIDESTQATEPEILVSIMRGVRQLVLVGDHCQLGPVVICKKAAIAGLSQSLFERLVLLGIRPFRLQVQYRMHPVLSEFPSNVFYDGSLQNGVTENDRHMTGVDWHWPKPNKPAFFWHCSGSEELSASGTSFLNRTEAANVEKLVSKLIKAGVQPHQIGVITPYEGQRSFIVNYMHTQGTLNSKLYENVEIASVDAFQGREKDYIIVTCVRSNDILGIGFLSDPRRLNVAITRAKYGLVLVGNAKVLARHDLWHELINHYKSKEMLYEGPINALKPLNLALPKATIRTKNNIAGNANRFGIKRMQYTFNEYKSNDPSQPRLPPTYSNSQNLLSMSKLAQTFNKNVPIPAHMMDPNVYAAARNQKDRRRGDQRRPPPQAEAAMDLSQGMMSQQSQQYPPQGASSQSQYLLDGASSLSGWSQSQTTTTTTRHHHHRQNRNSQQQMSQDMDDIQQKMDDLLFSQDC

>SMG-5

MQKSDEVTEKFKRYCNQLEKYGQTENVHSPVMAMLRRKGRKQLIEIMKRDGDCTSSINKLWIVGYYHPFQFFIRDKEKNMAIAVLLTMFCGELQEMLSLPDDKYPALWNMYIGDFHRYMPDEEIQKCLAVGYYSRAIDLDPNQGRAFHVLAGLRADLNVAQKLRLMILGQLADAPYKKGTELLEYLKFPQKESTDKLMVDFVIWALNEKSKRMDYQMTGIKIVNEFKAEIEQKLEFDWSLIMSTCRLASKLAMKKFGFQQFYNCFDTISTLYITIYSRTISSKCLLAEAISWISDSAEILGHLDEQKNEPHFQKLSVFAKTKWNELNDLVMNHINSVFTSMSLTINPSISMTSFLLNGPISEPNVEFLSQLINYLVSVEFPPMEIIHDREESGPLLRRINQSEQKRLDIQIKTQNDEVNREDWRPVYVLMDYDVIVDKIRIALKIWDIDDFICILPSTVLDELDYQKTKNRAVRPVIRALMELQAEGKIVLKKCDNERSCAEQLVQSARRSAEDHKHIVAFLCKNPSEQKEMEGVTFYDIDQFYMKYLE

>SMG-6, isoform a

MNGDTDASSSRTGAPRKPRPEIQIYRPGMLRQGGSTKSLSSTNEEARPPRPEKLNTTTTTMSGNSRRRSNDTDSVTSRGGSGSTTPDANVINAMNERGGGRNRFEKGYRQQQRTDGPSNYNSTQSLYDTRQHGGYMEYQGNHRSNHRNNGRYSNQQHQQHRQPFERPGGNHYTRGFNERASMRGDGAIARRPQGSRRQRNDSINSTQSEMPPQTGAQLHIDTSFDSASQCGASSNFSMNSLGEAFSFEEMCQNLQNFASMDWSKEVENEFAMQKEQEEEEERRMEQKLQEAAAQEAQKAASPRRNNRRNNRRGGGNSRYHPDESDRESSFGGSIAEEKEEEEAEEELKSGERTPTSVRSGNSYNPRVLYQRTRGANDENHPKRRDAAVEKKPKEPRRYRGEHDDRDDDASEYHGTLENHMSSGRLAGRIQIVQRSQNGTEAGGNRNRQSSEEKPRTYQKRSQNQSEMANRAEINRRRQEDIDRNEGKQIREISEKIEKLSAPVKKRDLKSAEKMAEISMELANIYSQVIIHDVIYSFTAGLEQKLFRQAFYKCIESLRTGSNSAAPDARLIRAVTQKLLLNGIVYYENLIATYETQFHVALTDALTWQSGSPSDEELCEQYIELPIGIQKFDSATQKTAIKSLSRHLISLGDLHRYKSLIDGSENYEISKSCYQKSSQLWPSTGHPYNQLGIVVYYSMLYRSARRARLVPVDVLSRQRQKRVIDEFFCLTRALACSHPYEVAKDRLKQRIDAMRTKVAKYQPVLDKESGIVKEQGNVLRRLQRIRQIWIHPITETSQDGTGERIVDDVLVHFMEHSKAKLHRRAVSYLCDTFGMLVTKIGMDHFESVSERAFGLLYASLSKSETDFSADQLVKFSAMFIYAAQVNFDKSTPSTSKSQLHTAVRTIFTYFLVLLEHISTHTTLLLPAANVIATWILHADTEELLSYLEHLEPLSSTIISSFSNFPPNLESEAENPENVTTPEAILLASFFKIYEPNPTPVRCSRSTNISQAVEKLKILGFSAEKSEKSGENGKQKLGDGLEEPKSREQMIDEQRGLGKTTIVIHPEYLIPDTNVLIGDLQLMKNLLETAQKNKKFQILVPTTVLDELQYIAKLSPLDSKSNSEAHDPERISKAKIAVAWLKEQAKQKTGHLYTLTTTGKRLPTLSIVSEDLEGHEMTTNDDMILNSALRWSESLPGSTAPSATEISQKCVLITGDRGLTIKAIGNNFPCRGISNFTKWIINV

>TSN-1

MTDAAAATPTVPPPAASSANPAVRRGLVKSVLSGDAVILQGQPHNGPPPEWTVYLSNVTAPRLGRRPTDSASATPDEPYAWDSREYLRQKLVGQFVTFVRDFTATSGRDHGRIYLGGTSPADAENVAEGAVSAGLLEVRQGKVADEYSTKLLELQEQAKSAGRGKWNSNAGTIRDIRWVIDNPRELVDKYAQKPIDAVIEMVRDGSTVRAFLLPNFEYITLQLSGVRAPSTRNPNAADSRAEAFSEEAKFFAESRLLQRDVQIILESTSNQNFVGSIVHPKGNIAESLLREGYAKCVDWSIGLCTGGAQKLRDAERQAKEKRLRLWKSYQPTSSAYSGDRKAFTGKVVEIVLSDAVVVQKDDGSEVKLHLSSIRLPRESGDDKATGGPGRQFRPLYDIPFMFQAREFLRKRLLGKKVQIQIDYVQPKSENFPEKTCATIKIGDQNIAEGLISRGLSKVVRHRADDENRSSEYDTLLAAEANAEKGKKGLFADKTAEKKDTHRIQEITGDLAKAKQFLPYLQRGGRAEGVVEFLSGGSRLRIYIPKETVLITFLLGGINCPKGARVGPGGVSTGAAEPFADEAAAFTRKLVLQHEVQLEVESTDKNGNFVGYLYVSPDGNTSRAINLSEALVENGLASLHFTAERSGHYNALLSAENKAKKAKKNIWANFTEEQHQEEVEVQQADTSERKQNFRQVAVTDIAPGALRFSAQNIEDGPKIEKMTTEMRQALAEHPPLAGSYTTKRGDLCVAKFSQDGQWYRCKVESVRAGQAEIVYIDYGNRETIEAVKLAQIPAGFANFPAGVREYNLALAKLPNEDYVQLTSDAFAQYLFGHSSVFINSEYKVGTSEYVTVYYDSGNKKVDIGKSLIAEGLALADHRREPRLQTLVNDYNTTEEVARKSRKNIWEYGDFTGNDI

>VIG-1, isoform a

MSTEYGCQVTNKFGLPSDDDDEYDDPRELIQKVSQIAAKKKEEKSVKPAQPVKPAAAPVAATKTDGAAGRGRGGRGRGRGGAGRPRDGERVSNENGDRPQGENRRGGPRRGGERGAARPAGRGGRGGFTRENREGEEPKQEVSFEDGQDTRAPRRRGGFTLGGGPSGGRGGGRGGRGRQFDRQSGSDRTGVRSFDKKDGHGKGNWGDQKDELAGETENIAPEGAESTEPEVPREKTAEELAYEAELAVLAKQKTLKEFKAAAKADAPKFNTRKAGEGAADTFGKLVPIKKEVIPDREEDEVVVIHKAPRKQVLDISITFRNDRPERERNDRSERPQRGGPRGGGRGGGRGGQRQGGHGGRNNTPFNASDDAFPALGAK

>XPO-1, isoform a

MAVSAMEVLSEAKRQFAQGDRIDVTLLDQVVEIMNRMSGKEQAEANQILMSLKEERDSWTKVDAILQYSQLNESKYFALQILETVIQHKWKSLPQVQREGIKSYIITKMFELSSDQSVMEQSQLLLHKLNLVLVQIVKQDWPKAWPTFITDIVDSSKNNETVCINNMNILSLLSEEVFDFGSQNLTQAKEQHLKQQFCGQFQEVFTLCVSILEKCPSNSMVQATLKTLQRFLTWIPVGYVFETNITELLSENFLSLEVYRVIALQCLTEISQIQVETNDPSYDEKLVKMFCSTMRHISQVLSLDLDLAAVYKDASDQDQKLISSLAQFLVAFIKEHVHLIEVTDEPLTEAKILMRESHDYAIQLLLKITLIEEMEVFKVCLDCWCWLTAELYRICPFIQPSTLYGMMSQVREHPRRQLYREYLSQLRSTMISRMAKPEEVLIVENDQGEVVREMVKDTDSIALYRNMRETLVYLTHLDNKDTEVKMTEKLASQVNGGEFSWKNLNRLCWAVGSISGTMVEEDEKRFLVLVIRDLLGLCEQKRGKDNKAVIASNIMYVVGQYPRFLRAHWKFLKTVINKLFEFMHETHEGVQDMACDTFIKISIKCKRHFVIVQPAENKPFVEEMLENLTGIICDLSHAQVHVFYEAVGHIISAQIDGNLQEDLIMKLMDIPNRTWNDIIAAASTNDSVLEEPEMVKSVLNILKTNVAACKSIGSSFVTQLGNIYSDLLSLYKILSEKVSRAVTTAGEEALKNPLVKTMRAVKREILILLSTFISKNGDAKLILDSIVPPLFDAVLFDYQKNVPQAREPKVLSLLSILVTQLGSLLCPQVPSILSAVFQCSIDMINKDMEAFPEHRTNFFELVLSLVQECFPVFMEMPPEDLGTVIDAVVWAFQHTMRNVAEIGLDILKELLARVSEQDDKIAQPFYKRYYIDLLKHVLAVACDSSQVHVAGLTYYAEVLCALFRAPEFSIKVPLNDANPSQPNIDYIYEHIGGNFQAHFDNMNQDQIRIIIKGFFSFNTEISSMRNHLRDFLIQIKEHNGEDTSDLYLEEREAEIQQAQQRKRDVPGILKPDEVEDEDMR

>XPO-2, isoform a

MEQIGAALQQTLEPDAAIRKRGEEALRSLQSNPGYIIQILQLVVNEQQQIAPQIRIAAAVALKNFVKRNWGPAPEVEMGQEDEEQFRSMLLEAMFNTKSNVQEILSNALYLIAQRDFPEKWPDLVPYLSKFLNGADLNHLVASLASMEQIFRKFRFESKSAELWKELKKCLLSTQEPLTLLLRNMMEVGQRKDQLGADEIAQWLRVLLLIAKVYHSLCSQEIPEYFEDHLKDWMPHFLHLVQIDAPTQTSNSGEPTTLDELKHEICEIFTLYSQRYEEEISEFVPDIILAVWNLLKSTGPDTRYDTMVCAALEFLSMVSQRQYYEGHFTGEGVLKTLAENVCVQNLLLRQQDMELFEDEPLDYMKRDIEGTDVGTRRRGAIDLARGLCRRFEAQMLPCLGEIVQNLLGSGDWIKIDIVYSLITAIAVKTETAKSGVTATNPLVDINDFFITQVATHLNADVNQTPILKADALKFAVTFRKQLAPEHLMTAIKSADALLSSNTPILHKYAAYAIEKILLADSNKIFSAQNLPVSSILQNLVTAFDKDAKAQNSPYLIKAILRIIVILDDDTIRHADAIAVKLAQLVESATKNPADSVHTHFLFETICVLITKTRTIGASLDAQLLPLIEVIFREDLEDLIPYALQITGVLVSSCIARNSSIDQFSPFLPFLLSERLWARSANVPAALSVLEVILSVNAQRVVSENSGLILSHLARLLGSKTLDQYGFQLAATILPSIEHFEGSAMTFVLNTMFRRVQSSKTPKFMKLFIVFLCRFTIARSAQDLVQSCENIQTGMFGMLIEKVVCIDLPGLKQTTTGPEKRIIAIGMGNLLADVTQQLVGQYGILSYEVAMLLEAASASDRAVLSPEEEQASMYNAEGEFVNPFCRLSYAPKQPPVAANIANHKAYFAQAVLVRGPGNCPETLRSVPPEIVTYLQSIQQ

>XPO-3, isoform a

MFGTNGNAGIAVTDPTKQAEIYRALESLKKDESGWKKSVDSFIGPHKPSPEEQFLLLQVIEDYLNKRYHSSSQGDVSVIRTFLLHYTKNSRSSTVDQPAFLTNKMAHIFSLVFAADFPERWSSFFNDLFFSDNINDRKVAFFYLKVLLAIDTEVVNRDIQRSKNESDRNIKIKDAMREICINEIAKSWLTIANALPEDNVIQCLVLDNIASYVDWIELDLVANDYVMPLIISKFQNPATSESATAAVCSLLEKGMPAEKKVGLTLTIMTVLRSNGLLTVNDNNDEEEVTRVGSLVNTLGLVLLDVQNKLCASSILEKEQECCVQEMAGLAEPALVVLNNEDPDLSCMCIDYIRAYCSFLLKFHPNETNFIEKVIRAGLQRYVMNDDMTVGGDGEDEVEFQEFRRELRSMLNVIGLKRPEAIINAVEPWTAEVTSGGSSIPVNRIEALLNVIFHLHEIIPSNMLQSPREGISQRAARLPIVILEGLVLDGRCPAIHVLYFELACRYERLLVLQPQPVVIPHIAAAFLDQRGISISSANVRTRIVYLFCRFVKSHKTVLGPLVSEVITRLAPLLAVSPQADTNQLLSPEDQGYIFEATATLIVFGDLTSEMKSQYVGELASTLAMKFENGLVELNTARARKADEETIQAILQFMSNIIGYSSRMSKAFNNAQSMKACNCIDIYLRLIKLYLETLSPQNAFLLESTRQFAHRLVVSMENELMPYMNGIFDKLALVSTDLDSMHHLLIFCHQTVAKYKKAMLTSGVDLGNVLAIAARASLQEQENNIPAKDDSQRALLYVQRAFVQLLYTVIASDCTPALNTTPGLLDHVLESAARLALSSDQTAQKVALSCLAKISLITPSWSARTLRVALEIPSLSHITPSDAGSTLVVHEVCATLTSLHQSDPDGFTRALRELVPNGFSDQLLSALTNLKGKNLDKQVMNLYSSLRNQSAQ

>ZFP-1, isoform a

MKEMVGGCCVCADENGWTDNPLIYCDGENCEVAVHQGCYGIQEVPEGEWFCAKCTKASAMMPGSINEATFCCQLCPFDYGALKKTDRNGWAHVICALYIPEVRFGNVHSMEPVILNDVPTDKFNKLCYICNEERPNDAKKGACMSCNKSTCKRSFHVTCAQRKGLLCEEGAISRNVKYCGYCENHLKKAINDPAIKVIPACPPVQRLSKEQDKKKTVLLTSLPLPPPAPRLHMLADPLPIKSNKVNNVLGLGSAINAVSLEERPASGSTVNSGIFVPPPTAFSPPLTTSSRSSVAQDPSPPLTINKNSLSSSGPLIPSTAHLSATTASATPIMANGSTLPPSSETTVGTHCLQQLQIQSAAAAAITQNQIGPSELNGYPAASQLSSFMHEIPARNTTSVASLLPPGAAEYHLNGSGDEEKTVKAVLTAPLTKAKRIRDSKNDMMDKTHKRPRANARPPAVLGSMSSGSSGGTVGKSPSMQRLQNLVAPIVSETVTDFQRDRVADRTAAERRAAAAQSQPSTSTNGGPNVTIPAVVEVHTNSTNSTNHQNNGLTQNAPASTSMQAGTSSNDGVISQNGTSSTSQSNRLNLPSFMEQLLERQWDQGSSLLMANAHFDVAQLLSCLFQLKSENFRLEENLSGLRKRRDHLFALNSRLAEVNTLDVSKRQRSDGLLLQQQIAHHLDPTSIVPKAEVHKQEPLSAPTSVPLPANHSSSLFEDIKAPKATYSRKTPSNIPLTVPLSTAATALTTTTAASSGAPVNSNIQNHRATPSTAGAPMAATPIMTAVTSANELAALSPERAQALLNMYRMPLDANVAAQLSMITNFPGQVNPSLFSRLLAVNMMNGGLQPNGQPLSALPPPTSATPNGK

>ZFP-2

MEEMMMNDPSAMVIYEEEVTTAPNLPCSLIQQRSWDEEKPIGYELTNTKCYKTPNGVQKTATIQREQLSTSKDFGEQQIVEMDGEFSIEGTDMHAIPCTSSSMQPSTSSNPSSGEHQPVPLRRMAIKIGQRVLRFKVISAEEAPEAPLDTQDSWINDPKPVTTPKALAGLYRCTNCKTYFGNKEVYQRHIQEVHGDARPFRCFNCGMRFANKTSMTHHLKDHSLLKPMFSCDYCPRIFSKLESKTRHHKMHFTRSTCQTCMRFFTTEDALRHHQSTAHPATFDSGPPPEDLLPNGKSARYSCSYCNLRFHFKKDMLVHERIHTGEKPYSCGYCMKSFAQSQALTAHIRTHTKELPYGCGKCDKRFRDNSCLRKHELAAHTDEPIVRPISVAYSNQVQKQMQRQRENRRKQELLIAERHPYRI

>HAF-6, isoform a

MSILSKLSQISIKSAVQSSKHCFSSYPWLHKPISRTTILKTWRICGFGAGLGAVTLRKAACAPKLSKRIDHLRTTEDQNASMTAGELWNLIKPFFGWFFAAVVCAILSAYINIQIPLCLGDLVNGIVKIIKDESNNLRSHFEQLKPSALHLMTLYVAQSALTFLYITFLTVLGERMATKMRSDLFQKLLHHDMAFFDSHKSGELSARLNADVQEFKSSFKLCVSQGLRTFAQTIGCIGSLYFLSPTMTMYTVAVVPGIILAGSAIGAGLRQLSRRAQGQSATASAVSDEALTNIRTIRAFAMEKLESRLFDNELDKARAMQEQLGVGIGLFQAGTNLFLNGMILSVLYGGSNLISKGEMTPGALMSFLVSAQTIQRSLSQLSIIFGTAIKGWTAGGRVLQFSRLEPSIPMDTGVCIPYHSLWGDIKFEDVSFSYPTRPGHTVFENLTLSIPAGQVVALCGPSGEGKSTITHLLERFYEPKSGRVTLDGRDLRELNVEWLRGQVIGLISQEPVLFATSVEENIRYGRPDATDEEVREAARAAHVDEFVSRFPSGYSTVVGERGAQLSGGQKQRIAIARAILKNPPILILDEATSALDSHSEHMVQEALNNVMKGRTVLIIAHRLSTIRSAQMIYVIKDKKALESGTHEQLMAKKGSLYRKLVEAHNVDS

>PIR-1, isoform a

MSNYHHNHNYQHRPRGYERLPGKRLPDRWNIYDNVGRDIDGTRFVPFKTPLDSSFFDGKNMPVELQFGVKTLISLAQQANKQIGLVIDLTNTDRYYKKTEWADHGVKYLKLNCPGHEVNEREDLVQDFINAVKEFVNDKENDGKLIGVHCTHGLNRTGYLICRYMIDVDNYSASDAISMFEYYRGHPMEREHYKKSLYEAERKKKYGKSSGKSSGNSADSTISSEQLHRNNSQ

>RDE-10

MSNHRSNFRDYQREGIRANNAGTSGDAVRQNGNPISVAKHVDGKKSVYMLFLRQIGQKKFLTEQGHRYNQNDQADKDIMTRYYHGMCPDLKQKFEREVAEHNGNRGLVIKTKHQRAQRNREMRHRNPDEFQQLRRAHLETLSQTPSVIALPRDINHVLHITEDLDAFCLANRKKAKRIMTSYIAQRSDDPGNPLLCEDYTMQIVSVFPVAYAFKPSINKLSSYPAEISVTTFNLKNGIIQNESRFVKFDAAWFYPDADDIGHEELSRKAMADELGISPNGPADGCEPYEVFEWLQHLLKQHPKSPILCDRAQFNFVYYGIKTLATYTGINAITFFQEVIIPSILSIQDFTSVILEKAPTDVPRVWRDVDICNQFQYHFLIPRTELNLFCSFHENKPSPTKYNCVKAHNARLLDNFFTVIKGNRLQGFVISPPVHEICIQDGSDTSLPQTILARTISRNDAEVYAARQRDTDEQYDVHQEGPSNHDQYEFASEPLDFEEDSDEENYNEQLDVPYSYNDHFISSSSVREPEHPSARSRDVAPNVQQESVVVPAPRRLSPQRAPRPSQNSPNAYSERKSFSAFPSEDPSEDYETPIISHIMNRNEADQYFNILDSVKPGQKYKIIKFDDF

>RDE-11

MNVITPPFADGVDPKPVCIVADDIFETMTARRKIHFSIYVIFKKNVELAEPFKLLYYTEKKNVAHLDGSVAQDFGLPMWTKDDGQRAPDLSRQKNDYIKLDFFTNINNPEDGFSYCWTDCISTKNTTMVPRQLMPPSKRSMKLYLILPVEESNIMVLSEKAYGSLQLMKKHRKMGQPPQRVGCYINFNCQTSKVMFGCGHVYCEQCLNSWNDKPCSVCLKPVTSEPTQLKLRQGPCPFDLCSPNSSTMGIVLIPCGCHVMCQNLEDAYERNKHHLEPLIEKIKYCPFEPCRIRVRKLKKPFWHQQDKEHTLEMNSA

>ERI-9, isoform a

MYRFCVALTPSTSEDEQVVRKSEKPASKPPTPEVEDCTFSEKNNDQRKIEKETESEESSNLKFPPARPSIRNATNFRRIICISESDSGKEEPVVKKEVLKTNKLRISETKPEYPPELLQLEARLQELRAGRKNKQGRINRKFVAKPCGVVSGETPQRPFLEEMYYFNPQETGTASEIFDMLFPAKDTPNFRPWLDGHFSAPDLQFKQIEKQRQTKLHSYTGTTLQEKKRKKRKKILKSIQEILWLLHFQKHDDFLESTSRLTIKNFSCHFDEHQRLKLLRNNDLSLICNYQIGSENTPIFNQRSTDCMLRPVFIDGLSFMDMLIPISSSLPIISTRSLLEILLNFILDGCQTVIYLPDYYNDTTNQKVDDSTIFQFLCNSKLIQFVDGRSRRAVERQVLLEAEKVHGVFVSSAEEFLTRNAIQCFPVINRYSKRLDEFLICTVFLVNNLPKKILAQDFVEESRVLKPEGSSSAVSSTTVLEESHQKISGAMEDSEDEPADPQTLKQEYLIKEECSEISEDSEEGAIVDVASSSGDLSENRDQKSENEEFEYSPHQLTLEQQTYLISSMAQLSIFHFQRYRQIRVFLMIIKRYVPELLPSFMQKTRMDDVAMAIQDIQNGKDLLYWPIFSKIDN

>NRDE-1

MEIDWLNAIPDSEDSEEDEEERQERARLLKEKEEEREACKKRYLERIEREEAQRFGKQFKIDPLTFAERLERKLIPAEKKQRYREFIPCVVDHFGGTDVHFVKAMESIKTENQLLDFLTAAHKPLIEQTEDSVLHDWLAEFYKETVTSLLEMTSITDHDLKCFKVGNVFDEVRTNAIQKKVQKMEMENFAERVREAKIKEAEERKATAEATRDVLESLEGMTLSHVKHDELEDELNTIALEELHSTTRDTSPPRTRLSTRAQSQLNAMVKHLFVPAAPTTSTEVPMEMDKDDDEMDAYDDSDLKILFSSTTKESLKMEMGEENDECNFEPSQVSKYPSFQASSQFDETRRYDYDEEGDNKELARLRRPTWRKILNYEKRIGTNFHRFTNAENANLWRRNLYEDKRKKTSKVQFVDYDSPLNKMRLFVRPNEQFFNADDGPMTDRIDFSLSDGFNVLATRKIMSPLVCGSNYDCEFMARTREGPQYLVRKTQSWMHSYCGNILGRFWYLRDRHADKYPGVKDSNYDQKRRQNFGLLRYYTLEELIEVFGLLLPDVQIAMRRGVRRENMPVSTVVKLKEKDREAVESPVQGVLLLTDWRLVHLFDNTAIVNAIIHYFPTNLGGVFFEAMTEYLTELLLDNTIEFHTIIFAFGGTPTFKKQDFLAFWKKLIVRTTQSIDVFWMAQDWCDGLNSTQEEIDQLNEFNHYVEQMFTMMRKNGNINYNYCDIRKQYNIPSLDPSFNRPNDFLLLEPIYRAYINHFVDECNCYLKGPFVKCQPRVHNGYAQRNGTKLEFSK

>NRDE-2

MFRAYGNNGLKNPERISGENPDLYTQTRAAVQQRATTTLKRNEKQKLAVQNDSVFQQVGIGESDSDDDNGGVRIRMSPHRYIDPDDVFTLPEVKKQNALRDAKIAARAAQATAYNTFPSVKSLNGCQDPPETSQQSTSRKRSASNSRSPSRSHSRRYDRDNGRQRSRSREKKRRKKERRRKRSSSRSSSSSRSRDRSSRARDTSSHTLMKMNKPAKYAFLTDEEYRTCDAYISSAFITQTKSDCENYTQGVPKKEIAKCRLSVKFIVGLEHNNILFNNIYGAEYARDKENRPFWEQLDRYLKDVPKETFFRYVPPVGGYWKIRDRVDLLNLDHIDDDVLANDSDNRKDAFTFELEQAKKTFSENVHNIDALIKVISMEEEMCRRNVGSFSSSNPAALAERHQEMVKKAIKADGRNAKLRLMKIELLIKMDPNSPTIIDDFKNLTITFPHEPMVWIKYLDYIQYDSNVYNYKKLKNAFEDCIRQVTGLTNGTLLSHLNAVNDRPLLRMFHLWIYIRYLKWMISCAHTPVVLANIQATFEYNFGLADVEKRTSTNSKEREVRLEEFWESGLPRIGDEGAVGAEKMLKQSEELSDEDIQKLENDDFDILISRTEETIATCLQAQRDVQISWIEVEREMMNIDARVKRTKLKDCELYEDHVDDLETCELWDIIPFDRIRYYEAPGDCANFDFVQPFLELLGVKFLNSTNCFTTTEQIISDWISNDSTVNFYKTPTYTEKKCFEVGNNILKFMLYNRLKLTENNPEYLDKTMVKYLLAMLVTEASEQEKKLNFHSFKLNLKNLVGTFITKHPDIFKRAMLSKITGIVYMEKFVSWWERALKEQEKVVEADERRKNYKEIKMEEGVVDDVKFDVILLKKDKERVQTIRDKIRDMIDIAIPKSTEKLIQSADSSLPTLQLHLYANVLRGRLSILNQNALEETRDVFCKEILGIHTSEFESDEALLLALDQGLNELLEHCKEKDNLESVDSIPELPRAEALCEALKVVAVFVFLDKMAFSRRAVDCLIANAITKFEQFEAKKNDFNRGTYEKYCDQIDLKFITDTLITFFSHKKHRFIYNENFKKLIFQASQAFPCDSKYAKMLGELHSSGRLQVMKLQGFTDSRNSILNAKRDQQFDPELETRLLMNSLTIMFSWMNAANRIGDAGNQILYKNWKREAANTRDPAIWRQVIRVASKLSQKILKDDAYTRARGQCTWALNLHFDYIEAKTVRKNGDLMEMIYLILEQSMGQEHSLFVTDEEYMKTQQEIGLQYSESGR

>NRDE-4, isoform a

MDFTRKIRKNRMMKPCCSTVCEMSHFEMSKNLQVLPKEQNKRNQSENAKLRKKQADKDSRERREIESLRERERHVEMCGSSGPEKYIVPISHFDLSRPTDRPMETVIEESVLSRRRAEEARNEQKHNEPLIDSNRKPIKLNIQAFKHGLHKSARQSNADEKLLLDNEHWMNLDNINSDDHKCPDGYFEPDTGQVLTMNVYRCPFVQAVIYSNSDRFFDLVIKNMNTIGRFCNQSCFCKQGRKNNLPHDKSNLLHFAAAYSTREFLERVTLEKFYRREIERRLRKQPKFLNDFVASIIIDQIYLKDTIDQYQKDPHGNERLIPSEVAAEEGRLDIVEVFLETSLSKSLANLKASATSKYCSFLQYRHWCYGSLLSRLVENGILHELSEKHLLRVFAYITTDNFLHAADTALAKGYGEALDILERLGVGFKSVYASKQYTQYSDPNTRNNDIPGIISSDGNELFKRCDSSQPAHILAQYGHFKLFDKLTVEEKNDTDFHRVSALIELLEFSDDEVRSQLRFFRNIRRDNIFLFISQVIIRNRIGLLEDALQLAFTNSNNFTPINTRKLIYKDYGTPEHIALPMETALQRTGNMDTVEQIAKYYTKTDRHARKMIEEDALRCIRSIQNRARVCTEHLRLSIIFMMIKFVDPFSPKFRNILRMYLDQAMNDNVSQYVLKSFSMIRSKRFLSKQSFVTWSTAVNEAFQIVSQWNGSLEEADFVPKFLSETKKCGAQMFFPKQEFRSLLLTEEKRTEKKKYQAERRPRTEDDVMSKLAKAIEDFENLNL

>CeAlg1

MSGGPQYLPGVMNSTIQQQPQSATSSFLPSGPISSTSTSSQVVPTSGATQQPPFPSAQAAASTALQNDLEEIFNSPPTQPQTFSDVPQRQAGSLAPGVPIGNTSVSIGEPANTLGGGLPGGAPGQLPGGNQSGIQFQCPRRPNHGVEGRSILLRANHFAVRIPGGTIQHYQVDVTPDKCPRRVNREIISCLISAFSKYFTNIRPVYDGKRNMYTREPLPIGRERMDFDVTLPGDSAVERQFSVSLKWVGQVSLSTLEDAMEGRVRQVPFEAVQAMDVILRHLPSLKYTPVGRSFFSPPVPNASGVMAGSCPPQASGAVAGGAHSAGQYHAESKLGGGREVWFGFHQSVRPSQWKMMLNIDVSATAFYRSMPVIEFIAEVLELPVQALAERRALSDAQRVKFTKEIRGLKIEITHCGQMRRKYRVCNVTRRPAQTQTFPLQLETGQTIECTVAKYFYDKYRIQLKYPHLPCLQVGQEQKHTYLPPEVCNIVPGQRCIKKLTDVQTSTMIKATARSAPEREREISNLVRKAEFSADPFAHEFGITINPAMTEVKGRVLSAPKLLYGGRTRATALPNQGVWDMRGKQFHTGIDVRVWAIACFAQQQHVKENDLRMFTNQLQRISNDAGMPIVGNPCFCKYAVGVEQVEPMFKYLKQNYSGIQLVVVVLPGKTPVYAEVKRVGDTVLGIATQCVQAKNAIRTTPQTLSNLCLKMNVKLGGVNSILLPNVRPRIFNEPVIFFGCDITHPPAGDSRKPSIAAVVGSMDAHPSRYAATVRVQQHRQEIISDLTYMVRELLVQFYRNTRFKPARIVVYRDGVSEGQFFNVLQYELRAIREACMMLERGYQPGITFIAVQKRHHTRLFAVDKKDQVGKAYNIPPGTTVDVGITHPTEFDFYLCSHAGIQGTSRPSHYHVLWDDNNLTADELQQLTYQMCHTYVRCTRSVSIPAPAYYAHLVAFRARYHLVDREHDSGEGSQPSGTSEDTTLSNMARAVQVHPDANNVMYFA

>CeAlg2

MFPLPVHNGPRLGKLSIFEMPGDSLTSSSFMPDGGAETSSSSQLGGSAHGAIGTKPDAGVQFQCPVRPNHGVEGRSILLRANHFAVRIPGGSVQHYQIDVFPDKCPRRVNREVIGCLISSFSKYFTNIRPVYDGKRNMYTREPLPIGTEPMNFEVTLPGDSAVERKFSVTMKWIGQVCLSALDDAMEGRVRQVPHEAVQSIDVILRHLPSLKYTPVGRSFFTPPGVMKPGMQMHQESKLGGGREVWFGFHQSVRPSQWKMMLNIDVSATAFYRAMPVIEFVAEVLELPVQALAERRALSDAQRVKFTKEIRGLKIEITHCGAVRRKYRVCNVTRRPAQTQTFPLQLETGQTIECTVAKYFFDKYRIQLKYPHLPCLQVGQEQKHTYLPPEVCDIVPGQRCLKKLTDVQTSTMIKATARSAPEREREICKLVSKAELSADPFAHEFGITINPAMTEVKGRVLSAPKLLYGGRHRATTALPNQGVWDMRGKQFHTGMEVRTWAIACFAQQSHVKENDLRMFTTQLQRISTDAGMPIIGTPMFCKYASGVEQVEPMFKYLKQTYSAIQLIVVVLPGKTPIYAEVKRVGDTVLGIATQCVQAKNAIRTTPQTLSNLCLKMNVKLGGVNSILLPNVRPRIFNEPVIFLGCDITHPAAGDTRKPSIAAVVGSMDAHPSRYAATVRVQQHRQEIITDLTYMVRELLVQFYRNTRFKPARIVVYRDGVSEGQLFNVLQYELRAIREACVMLESGYQPGITFIAVQKRHHTRLFAADKADQVGKAFNIPPGTTVDVGITHPTEFDFFLCSHAGIQGTSRPSHYHVLWDDNDLTADELQQLTYQMCHTYVRCTRSVSIPAPAYYAHLVAFRARYHLVDRDHGSGEEGSQPSGTSSEDTTLSSMAKAVQVHPDSNNVMYFA

>Alg4/Tag76

MSRRNATSFVDNNTLTSSGISGSGSMSPPITSRPASGQASPLTSNGSLSPPQYADDQGSVSYNLDSPRDLSPLLLSELACLNMREVVARPGLGTIGRQIPVKSNFFAMDLKNPKMVVIQYHVEIHHPGCRKLDKDEMRIIFWKAVSDHPNIFHNKFALAYDGAHQLYTVARLEFPDDQGSVRLDCEASLPKDNRDRTRCAISIQNVGPVLLEMQRTRTNNLDERVLTPIQILDIICRQSLTCPLLKNSANFYTWKSSCYRIPTAAGQALDLEGGKEMWTGFFSSAHIASNYRPLLNIDVAHTAFYKTRITVLQFMCDVLNERTSKPNRNNPRGPGGPGGPGGYRGGRGGGRGGSYGNFGNRGPPGANVRDDFGGNGLTFTMDTLSRDTQLSSFETRIFGDAIRGMKIRAAHRPNAIRVYKVNSLQLPADKLMFQGIDEEGRQVVCSVADYFSEKYGPLKYPKLPCLHVGPPTRNIFLPMEHCLIDSPQKYNKKMSEKQTSAIIKAAAVDATQREDRIKQLAAQASFGTDPFLKEFGVAVSSQMIQTTARVIQPPPIMFGGNNRSVNPVVFPKDGSWTMDNQTLYMPATCRSYSMIALVDPRDQTSLQTFCQSLTMKATAMGMNFPRWPDLVKYGRSKEDVCTLFTEIADEYRVTNTVCDCIIVVLQSKNSDIYMTVKEQSDIVHGIMSQCVLMKNVSRPTPATCANIVLKLNMKMGGINSRIVADKITNKYLVDQPTMVVGIDVTHPTQAEMRMNMPSVAAIVANVDLLPQSYGANVKVQKKCRESVVYLLDAIRERIITFYRHTKQKPARIIVYRDGVSEGQFSEVLREEIQSIRTACLAIAEDFRPPITYIVVQKRHHARIFCKYQNDMVGKAKNVPPGTTVDTGIVSPEGFDFYLCSHYGVQGTSRPARYHVLLDECKFTADEIQSITYGMCHTYGRCTRSVSIPTPVYYADLVATRARCHVKRKLGLADNNDCDTNSRSSTLASLLNVRTGSGKGKKSYAPSVDDESYSLSDATSDQILQDCVSVATDFKSRMYFI

>CeR06C7.1

MSPHPPQPHPPMPPMPPVTAPPGAMTPMPPVPADAQKLHQSTGNDACIKRLQQLNVEDGAKMYMKPTEPGKMGRPVDIQTNVFGIEVTKETTVHRFMVHAKADLTSTKEVTFTKKGKEDFVVQDRRDKCCNIFFLAVEKNPEFFKMKDGNQIVYDGQSTLYTTVNLFSELDANGTKSKVFQINGADTGNDDLKTLPCISLEIYAPRDNSITLSSENLGKRTADQNIEVNNREYTQFLELALNQHCVRETNRFGCFEHGKVYFLNATEEGFDQRDCVDVGDGKQLYPGLKKTIQFIEGPYGRGQNNPSLVIDGMKAAFHKEQTVIQKLFDITGQDPSNGLNNMTREKAAAVIKGLDCYSTYTNRKRHLRIEGIFHESATKTRFELPDGKTCSIAEYYADKYKISLQYPNANLVVCKDRGNNNYFPAELMTVSRNQRVTIPQQTGNQSQKTTKECAVLPDVRQRMIITGKNAVNITLENELLVALGIKVYSEPLMVQARELDGKELVYQRSVMSDMGKWRAPPGWFVKPATVPDLWAAYAVGNPGCRFSIGDVNQLVGMFIDSCKKKGMVIKPPCETGLYSTEKIMTQLEKVAASKCKYVLMITDDAIVHLHKQYKALEQRTMMIVQDMKISKANAVVKDGKRLTLENIINKTNVKLGGLNYTVSDAKKSMTDEQLIIGVGVSAPPAGTKYMMDNKGHLNPQIIGFASNAVANHEFVGDFVLAPSGQDTMASIEDVLQNSIDLFEKNRKALPKRIIIYRSGASEGSHASILAYEIPLARAIIHGYSKEIKLIFIVVTKEHSYRFFRDQLRSGGKATEMNIPPGIVLDNAVTNPACKQFFLNGHTTLQGTAKTPLYTVLADDCKAPMDRLEELTFTLCHHHQIVSLSTSIPTPLYVANEYAKRGRDLWGELTTKGPIEAKESQGERLKELTKEIGYKQTDLNQKRVNA

>CeC04F12.1

MPSKKNKKAAAAERSATLSASASQQVAKDEQSAPVVPTPELSRVSVSSNPPTPSAIEKPVPLPVPSKPLFEYEPPAQLEALAKNKPVKVTTNSYELELKPTPVFRYDVSVLKSFDDSTRDPIELAGSKGGDRQRQADLTEIVKVALTLEGLGNLNIFYDGAAMLFTTQKFETKTEKQQAIFMPIPSDHPKLSTSLKESQFGRASGKFHVSIELNSKQSELNTIDLLNERLDSAACPVTQMAQIALSAEAKQNGFIILDGGHELFDQKNTRRTIGKDGVEDMDGVAASIKMAQGNKKTGSAHLVMDYKKKQFFKTGPLKDLIKGINPNQLRSYLKGLRVHTTYSHQSIFIDGISNVPIGEIKLPDGTPLLDDCARVSGKPKSFFDASKPAVQVNQYNKREGRRMVYSFPIENLLVKPNQKLTQNHGEPPRSIKPELRFDLIRKVGESAKLLSPNQTLKSIGIAINPEPIVVEAMTVPKPTILYKDGAFTSPDLLNRTSWDVQARNHQSLKGGFVEPMKINKILILYNSQSYSQASPKEAVVELQSILSKKAKDVGMTIGQIDVEDLNDPDIIGAIENKMTILKTSQVKPIVIYADHTSEGTHSTLKLQERLCEVITQQVALDKSLKRTPGKVTANNLLMKLNLKYGGVNHKVRVDNSISHLWGDTSNTLIISYDVCHSSGKVYKKDEICDEPSCVGFGFNGTACPEAFIGDFHYQLPRHEQVDEHLLKLRARFMLNHYITSRKKYPQQVVILRDGVSEGQHKMVRNEEFQAIKQSIMNVFNEKKAKAPSFALLVVTKRHANRLLIKDQNGYTNVPPLTAIDQTIVKKQGNEVIFVSHCPLNGTAQPIVINTLVNDGIFKTNDQLVQFITALCCAHQKSTNIVSLPESIYAADEYAKRGADLFQSYKLKHRDLPTTTIDDSTQLDYEKITLILCFQTSSFKNKRIA

>CeF58G1.1

MPALPPVYTPSGAPSSVHAPPAVPPVPVPTQPLRSEYQTSNDACIKRLEELNIAPAAKLYPTPTEPGKCGVEAEIQTNVFGIEMHQDSLFYQYSVNITTELKNGKEVTFTKKGKDDFVVTERHDKCCAILFRALGDYEEFFKTSDSCLIYDGQSILFSNVDLFQGFREGAVKTKYMQLDGGEMDHKDLKSLPCIKLEVFPTKNPAVKFTREAVARRATDSNLDSVSLAYQQILELALTQPCLRNTARYVVFDHGKMFFIDPLGEGFEKCDVVDVGDGKQVVPGLKKTINFIEGPYGRGRSNPSVVIDGMKVAFHKNQPILDKLKEITTQPVEHGLKGLEKDRCAAVIKGLDCYSTYGGRERHHKIEGIHHEGARNARFELNDGGSCTVAQYFEDVYNITLRYPDTNLIVSKERGNINFYPMELLKISSHQRVQIPQLTSAQSQKTTKESAVLPDVRQRLILTGKNAAQISSDNEVLGKMGVSVCEDPLMVKGRSIPAVKLANAEIGANPINVKDNKWRANRFTRPATAPNVWAMYVVGTASTRITLDTLKKFADEFAAMCKSKGVNMPAPADISLIHMDAIESRLYDATKANCTFVFIITDDSITTLHQRYKMIEKDTKMIVQDMKLSKALSVINAGKRLTLENVINKTNVKLGGSNYVFVDAKKQLDSHLIIGVGISAPPAGTKYAMENKGVLNPNVIGYAYNAQHNQEFSGDFVLNSASQDTLAPIEDIVMHSLNEYQKFHDGGLPRRVIVYRTGTSEGNHGSIMAYEIPLARAAMRDFSPDIQLVYIVVSKDHSFRFFKPDLASLASRPQATSSTASRHSAMPAAPKAWDLNIAPGILVDSIVTNPACKQFFLNSHITLQGTAKTPLYTVLADDAKVSMTALEDITYKLCHLHQIVGLPTSLPTPLYVANEYAKRGRNLWNEAVALNNVPTVSGPEADRLKELTKSICYKASGDLTGRRVNA

>CeRde1

MSSNFPELEKGFYRHSLDPEMKWLARPTGKCDGKFYEKKVLLLVNWFKFSSKIYDREYYEYEVKMTKEVLNRKPGKPFPKKTEIPIPDRAKLFWQHLRHEKKQTDFILEDYVFDEKDTVYSVCRLNTVTSKMLVSEKVVKKDSEKKDEKDLEKKILYTMILTYRKKFHLNFSRENPEKDEEANRSYKFLKNVMTQKVRYAPFVNEEIKVQFAKNFVYDNNSILRVPESFHDPNRFEQSLEVAPRIEAWFGIYIGIKELFDGEPVLNFAIVDKLFYNAPKMSLLDYLLLIVDPQSCNDDVRKDLKTKLMAGKMTIRQAARPRIRQLLENLKLKCAEVWDNEMSRLTERHLTFLDLCEENSLVYKVTGKSDRGRNAKKYDTTLFKIYEENKKFIEFPHLPLVKVKSGAKEYAVPMEHLEVHEKPQRYKNRIDLVMQDKFLKRATRKPHDYKENTLKMLKELDFSSEELNFVERFGLCSKLQMIECPGKVLKEPMLVNSVNEQIKMTPVIRGFQEKQLNVVPEKELCCAVFVVNETAGNPCLEENDVVKFYTELIGGCKFRGIRIGANENRGAQSIMYDATKNEYAFYKNCTLNTGIGRFEIAATEAKNMFERLPDKEQKVLMFIIISKRQLNAYGFVKHYCDHTIGVANQHITSETVTKALASLRHEKGSKRIFYQIALKINAKLGGINQELDWSEIAEISPEEKERRKTMPLTMYVGIDVTHPTSYSGIDYSIAAVVASINPGGTIYRNMIVTQEECRPGERAVAHGRERTDILEAKFVKLLREFAENNDNRAPAHIVVYRDGVSDSEMLRVSHDELRSLKSEVKQFMSERDGEDPEPKYTFIVIQKRHNTRLLRRMEKDKPVVNKDLTPAETDVAVAAVKQWEEDMKESKETGIVNPSSGTTVDKLIVSKYKFDFFLASHHGVLGTSRPGHYTVMYDDKGMSQDEVYKMTYGLAFLSARCRKPISLPVPVHYAHLSCEKAKELYRTYKEHYIGDYAQPRTRHEMEHFLQTNVKYPGMSFA

>CeC16C10.3

MADLLDKIMGSSSSNRIPKRDNRMNQDKDEPTSKRSAPMFSTPKSTTPIGRDSFATMDTMNVQMNMFPVDIKDMPHKIQRFQVDVIVCSSNGKQINANLGVLAAKGDVNSHNRRLAQYYIMRTVHDKLLVKFSGKSHHFLAYDCAATLYLPEGVYTGDDQEEVTLTIDDFPKEEWKFVSKLSRRKDDSYLVVLKPAGFVYTQGEVAQEEANRMELTRIIEIVTSQKLNNEDYLQFGNATFPRLSPPHSEPDAISEIRSGFAKVSRLSQNGGGKAFMTVDTKISPFYKDTSVIKFSSNKLSEMKGGGGGRGGYGRSDSRDSRGGYRGGRSDSRDFRGVYGNRGGNDRYRDESRGRRDMYDSRRDSGSSNGADYSPSDAAELEHAFGERGNTKRCIEEALKGLDVECTHLKGNLIRVSSIAENNAENTSFMMKDDKGEREVTVAEYFLLQYNIKLKYPRLPLVVSKRFKHESFFPMELLRIAPGQRIKVNKMSPTVQSAMTGRNASMPQHHVKLVQDILRDNLKLEQNKYMDAFGIKLMSTEPIQMTAKLLPPAQIKFKGQTYMPDMSRPAFRTQDKFVEPARIRKIGIVVFDNCIQMRQAEDFCDKLSNFCRDNGITVEKDSRDWSIRELNSSDSVAIQNLMKKWLDDRVDILVGIAREKKPDVHDILKYFEESIGLQTIQLCQQTVDKMMGGQGGRQTIDNVMRKFNLKCGGTNFFVEIPNAVRGKAVCSNNETLRKKLLEHVQFIGFEISHGASRTLFDRSRSQMDGEPSVVGVSYSLTNSTQLGGFTYLQTQKEYKLQKLDEFFPKCVRSYKEHSKTLPTRIVIYRVGAGEGNFNRVKEEVEEMRRTFDKIQPGYRPHLVVIIAQRASHARVFPSCISGNRATDQNIPSGTCVENVLTSYGYDEFILSSQTPLIGTVRPCKYTILVNDAKWSKNELMHLTYFRAFGHQVSYQPPSVPDVLYAAENLAKRGRNNYKIHQRYVNLQAVENRIIKDHSELINEDMREELAAAIVDEMSVAMNGMTIPKRNFWA

>CePPW1/SAGO2

MEKQLKAMSVSDKPAAPAAQKLGTAPLAAKKTRNEEWGTKVNIDTNIRKLTIKPNQPIYKYAVQVNYVFRKPDGTEATIEMSKSAKKGTEHDNDKTRCQNVYNEAIKRYDELKTGGPFFYDRQASLYTLTKLKNESISFVVTDKICKRQNFKEAQFVLKKVDQSFQSTSNDVIKTTNSCPANADKTLLEAMNIIVSGPAFENKNVITVGACVHYLIDPTGVDVAYKEYPEGQLYSGVGVSKSVKTLEGTDKKVPSLFMTTEMKTTLFHPDYAPLVELLQTFRGFSTTLKANSPAAQRIEKAFVGLDVVLNYGVHKGLGEDGVVMKIRRFHTSAKETCFEVEKSTREFTNVFDYFKKKYGITLKYPDLFTIEAKGKQGKIHFPAEVLLLCPNQTVTNDQMINNEQADMIKMSAAQPHIRKTTTDTIVRNVGLASNNIYGFIKVEDPVNLEGMVLPKPKIAFAGNRLADLANPKSRFPTDFNRAGQYYDAKELTKWELVFVQNEEVQGLAKQLADEMVNNGMKCSNPTMSFIIRGDLEPIFKKAKAAGTQLLFFVVKSRYNYHQQIKALEQKYDVLTQEIRAETAEKVFRQPQTRLNIINKTNMKLGGLNYAIGSEAFNKPNRLIVGFVTSQRVGGNPDYPISVGFAANMLKHHQKFAGGYVYVHRDRDVFGSIIKDTLLTIFKTCTEQRGRPDDILLYFNGVSEGQFSMINEEFSARVKEACMAFQKEGTPPFRPHITIIASSKAHNERLYKSDKGRIVNLEPGTVVDHTIVSNVYTEWYHASAVARQGTAKATKFTLIFTTKAGPQAEPLWHLEQLTNDLCYDHQIVFHPVGLPVPLYIADRYSQRGAMVLAANQG

>CeCsr1

MNQKQNPRLALNIFGLELSERTIFRHVVQMKLIDRQHNKEYILTTMSARGRGNRATKQKDNFILLDILLKQWAAKKGQQNLPAFAYDGAQSLFTLEGISLMVDIKKEDALEIPELSNFLKDSISFLSGDLEISCEPDLEKPSFVQTELNEWSDPRFYAYLDIVTSQSAIRSERYLSQSKGLYVHTQSLEELRVKWAVAAKGIHKGCRIVGANGPLPILELDPQSTQYYASIPLSQMLQYAFPRDFPPNRIVNPNMKLQRAVKLLLKDLKCNPYYDDKQIWATNTITVSDVDYNAPKDPEFRQKYPNLKFPMLPAVQCGTGPHKRLMPLEYLKVLPYQSIDRRVLEEFELTPRANAPNERWSTLQKHYDQFGFNDQVMKDFGVQICNDPFNNVSEIDGERVLAPSVAYADPVHVDDEKRDWKAQDKKFVTPATIDHLMFVLVAGYTRTWDADCDATKFVAKAFMQRCKDKGMHIGSYSMDQHNGERGSENFLTSVFKNLVTHPNYRDSSFTPFVLFISDDVPNIHECLKFEERMSDIPTQHVLLKNVKKMRDNIEKKSQGGRRAYDLTLDNIVMKANIKCGGLNYTADIPRDLACWNEVSTFVIGMDVAHPDRNAAREGNPSTVGLSCNSAENPYSFIGDFLYTDPRREAIQDEILRKFTDQSVRNFAEIRGFPKKVIIFRDGVSFGEETAALKEVEIIEQTIKTAAKSMGHSDYAPKVLAIVVKKRHHTRFYAKGGHHGNMPINPLPDTSVGGDIAEYGKRQIFIQAFRPVQGTAKVPSFLVIRDDEEVSDEHVAKMVCAVCSLHQLVNSPTSIPTPVYVAHELAKRGTGLYKAYRFKNGELFDDWETLTTQLSYSTLDRLSKVRVV

>CePPW2

MPATPVPPVTMPPVPPVGFPPVTAPPGLHPPPPVPPVPVPTLPVTSEHKTAHDACIKRLEQLEIPPDPKIYPSPTEPGTFGSVTEVLTNVFGVEVHKTSNFYQYSVHITAELSSKKEVTFTKKGKEDFIVHDRHEKCSAILFHALNRFEDFFKSSENCIIYDGQSILFTSMNLFEGIPTGKIKTKVFQINGADTNLKDLMVLPCIKLEVFPTKNPVVNFSLEDIGRRTSDSNIESVNMAYKQILDLAMSQYAIRETSKCVVFEHGKMFFINPLQEGYAPCDIVEVGDGKQMKPGLKKTLQFIEGPYGRNQNNPSIVIDGMKVAFHMEQLVFQKLSSITSNLANGITGSERERCAAVIKGLDCHSNYRGRTRHHKIESIHHEGAATARFELEGGGTCTVATYFKDKYKIQLRYPNANLIVCKERGALNFYPMELITISPNQRVRITQQTSSQSQRTTKESAVLPDIRQRLIMTGKIAAKITAENEVLGKMGVTVCDEPLVVKGRNLPAIRLASFETGEHLINPRDCKWRPQRYNRSAVAPKVWALYGVGSPGSQMNRDVMRRFCDEFMNMSRSKGILFPPPGDVNLLTPDAIENRLREAANAGCTFVLCITEDNITCLHQKYKFIEHHTQMIVQDMKLSKALSVVNASKKLTLENVINKTNVKLGGSNYVYLDTKNFLQEHLIIGVGISSPPPGTKYIMESRGILNPTIVGFAYNGNGKQEFSGDFVLNAAGQETIAPIEDIVSYSIKGYKKFHDGKAPKRITIYRSGSSEGNHGPIISYEVPLARVAMRNFSPDTQLLYIVVSKEHTYRFFKKESGGSSSGGSNSAGASNSGTLTSAPPKPWELNIGPGLTVDYGVTNPACKQFFLNSHMTLQGSAKTPLYTVLADDRNIGMSALEEFTFNLCHLHQIVGLPTSIPTPLYVANEYAKRGRNLWNEAINANEIPEVTGPESARLKKMTDGISYMGSGDMIDRRVNA

>CeSAGO1

MSNITQVTSSMASASLSNKAPLPVGHQPLAEKKPKEVNQEGTPVQIVTNMRKINLEKNHSIFKYSVQVLFVYQKSDGTELVLEKSKSVGSGCDHERSKSHCLRVYRKAAKQCQELKSGGPFCYDSQGCLYSFSKLKNDEFSTNITGSDISNNPKFLRVEFKLAKVQESFQTTTNDVAKSVNCRPALQEKTILEAMNQIVSTAPINHPNVLTIGNCVHYLYDDTNIDIRSITGEGGKSSAVGASKSVRTLEGTGKTPCLYMATELKTTLFHPDNCSLLKVFMDYRGFNGSLKANSPFVLKNKNAFIGLWCYTTHGKCSDWKDDRPMIKIKDFGLSAKETTFERDNKKISVFNYFQVKYNMTLKYPDLFTVVARGKDGKNQHIPVECLDLCNSQTVRTEQMVGTEQADLIKLAAAKPHDRKKITDTVVNSIGLASEPKGIISVGAPESVTGLVLPKPDIYFSGGKKVFWNDPKKRGPATDFMPAGTFIKPTKLTNWEVVFDNGVQLVDCIQHLTSTMRQLGMEVSNPTVSLINRGYLRSIFENAKAANRQLIMFITKSMNNYHTEIKCLEQEFDLLTQDIRFETAVKLAQQQNTRKNIIYKTNMKLGGLNYELRSGVFSNSKRLIIGFETSQRGGLGDAPIAIGFAANMMSHSQQFAGGYMFVKKSADNYGPVIPEILLTILKQAKANRPNDRPDELLIYFSGVSEGQHALVNEYYANQVKAACGLFNESFRPHITLILASKVHNTRVYKSENGGGVCNVEPGTVIDHTIVSPVLSEWYHAGSLARQGTSKLVKYSLIFNTKKNEKLSVYERLTNELCYEMQIVFHPTSLPIPLHIAGTYSERGSQMLALKKPIYTNGEFNQVATNEQLGYASKKLFGTRFNA

>CeT22B3.2

MSRRNATNFVDNNTLTSSGISGSGSLSPPITSRPASGQASPLSSNGSLSPPVDDQGSVSYNSDSPRDLSPLLLSELACLNMREVVARPGLGTIGRKIPVKSNFFAVDLKNPKMVVVQYHVEVHHPGCRKLDKDEMRIIFWKAVSDHPNIFHNKFALAYDGAHQLYTVARLEFPDDQGSVRLDCEATLPKDNRDRTRCAISIQNVGPVLLEMQRTRTNNLDERVLTPIQILDIICRQSLTCPLLKNSANFYTWKSSCYRIPTAAGQALDLEGGKEMWTGFFSSAHIASNYRPLLNIDVAHTAFYKTRITVLQFMCDVLNERTSKPNRNNPRGPGAPGGYRGGRGGARGGSYQNFGNRGPPGANVRDDFGGNGLTFTMDTLSRDTQLSSFETRIFGDSIRGMKIRATHRPNAIRVYKVNSLQLPADKLMFQGIDEEGRQVVCSVADYFSEKYGPLKYPKLPCLHVGPPTRNIFLPMEHCLIDSPQKYNKKMTEKQTSAIIKAAAVDATQREDRIKQLAAQASFGTDPFLKEFGVAVSSQMIETSARVIQPPPIMFGGNNRSINPVVFPKDGSWSMDHQTLYMPATCRSYSMIALVDPRDQTSLQTFCQSLTMKATAMGMNFPRWPDLVKYGRSKEDVCTLFTEIADEYRVTNTVCDCIIVVLQSKNSDIYMTVKEQSDIVHGIMSQCVLMKNVSRPTPATCANIILKLNMKMGGINSRIVADQITNKYLVDQPTMVVGIDVTHPTQAEMRMNMPSVAAIVANVDLLPQSYGANVKVQKKCRESVVYLLDAIRERIITFYRHTKQKPARIIVYRDGVSEGQFSEVLREEIQSIRTACLAIAEDFRPPITYIVVQKRHHARIFCKFPNDMVGKAKNVPPGTTVDTGIVSPEGFDFYLCSHYGVQGTSRPARYHVLLDECKFTADEIQNITYGMCHTYGRCTRSVSIPTPVYYADLVATRARCHIKRKLGLADNNDCDTNSLSSSLASLLNVRTGSGKGKKSHAPSVDDESYSLPDAASDQILQDCVSVAADFKSRMYFI

>CeT22H9.3

MPTTVQNPSPTGFLPQIPFRIPKRTRRSSEVDVPATKRPAPPLTSSTSLPTRDSFSIIETRDVQLNMFALNISGMPRKIQRFHVDTLIKGKNGKIFNVNHGVLANSGDINSHNKRLAQHALMQKFHKKRRELFGQHDYHILAYDCANTLYLPDGVYLGRNEEECTFKSDDFSPEQWNITSKLSRKKDYTYVMKIRPSGFIYTSGEEASEVANRMELTRCVEIVTSQKLNNSDYCQFGNDTFSFRAQPVSEPDATSEIRSGFAKVSRIVEGRNGKNEMLMAIDTKASPFYKSTSVLNFVCGKYLEKRGSSRNDYRGRGDYNRKKTGGGSTDAPDHQDVAEVERSLEYRENRQQIEEALKGLLVSAAHLKNSTNLIMISRVAETNSETTKFMTNNGEREISVADHYLEAYNYRLKYPKMPLVVSKRFRDECFYPMELLQVAPGQRIKDHKMSAAVQMAMKGQKSTLPQKHVDLVKHVLSRNLKLDRNLYMDAFGIKLESAELVKLQSKILPPPQIKFKDEVYMPKMGNPVFRTNGRFVDPADINAVAIVVFDRAIDMRQAENFCDRLSCYCRENGITVKKSSRDWPIREINSEDSVAIKNAMEKWFSKGVNILVAIAKEKKPDVHDVLKYYEASVGLQTIQVCKQTVDMMLSSGGRQTADNVMRKFNLKCGGTNFFVQIPRSVNGRTVCADAHLLNEKLFERVQFIGFDISHGASRTLFDRSTGKMDGEVSIVGVSYSLSHSTHLGGFAYMQTQKEYKLQKLDEVFPKCVDSYRNHTGRLPSKIVIYRIGAGEGDFKRVKEEVDKIRGTFNRIQPDYRPQLVVIIAQRDSHVRVFPAHITGSKAGQQNVTSGTCVDSVITSLGHQEFILSSQMPLLGTVRPCKYTILTNDPNWTKTEITHLTYFRAFGHQVSYQPPSFPDVLYAAENLAKRGRNNYKVHQRFVNLQAVERSVIAENSDLVSEEMREQLAAAIVDEMSAAINGMTISKRNFWA

>CeERGO1

MSYNNGGGGGGGGYRNDRDDRYHNNDRQNYRSSDQGRSGYNDDRRDNRYDDRRGSNNDRGCYDQHDRRGSSNDDRRGYRGYNQGGGGYQQQYSQDARYGSNQRNDNYGNNRGSHGGANMYSQNGGNRGGGGGRVGGGRTAAGMSNPGDLVGGADQPIHSVSKKSLRHNAQEFAVRPKTMVQDKGLGQKTTLLTNHTLVQLPQEPITLHVFNIEVFINGKSSNKRELCGPRFWEILKENKPTFGMPNQYIFNDVNMMWSTNKLRQSEGRTNNRRMNFVWKYVKQIKFGGNIEDEETMQLLSTLIDAIATQRARLPLAPPKYTVFKRLTYLICEEAYEPELPDVSLCHKLRIGTDARVGVSIAIRTNLRAGITACFDLGHTLFTRPAYPLVRLLCDIIEHSVVLDEAFEMKYDAALRACNVSDENLRVMTQILTKMTLQLSTETGDYVGEDGEVIVRPAPTIRNPGRNFKFVGLGAPADRYYFTSDGVELTVADYYLQKYNIRLRYPNLPCVLKKAPEQCGNKHSAMPLELVSYIVVPTRYGGFTMPDMRADMINKTTYTAQQRGKLLQHIIAQKSLSGIEPPVSNNDDYMKKHKLVMKREPIRVKATILPPPTLVYGDSVFHDEHHIGEWEAVTHDPPRQVLDGAVFRRKLYKSSEQPLMKRLMGSILLIQSPRQCRDFDYNQQGYHAIMRAIEDSGQPVLWADENKHSAVIQGELQFNQNQHGIEVIEQFLQNIKSTIGEYERDGEVIVPIVFAVFQARATVYSGNNNEYNDYNVLKYLADNKYGIHTQGILEKSLGVVGPSPKNCALTRLMVEKVLGKVGTTHRKLERGGAHKTWTIFTDPAKPTLVLGIDVSHPSTRDRETGNVLQKMSAATVVGNIDLDVTEFRASSRIQDTGVECLIDFSKEIDERIGEFIDHTGKRPAHIVVYRDGLSEGDFQKYLFEERVCIEERCLKIDTSFQPSITYIVVTKRHHTQFFLEDPSQGYESQGYNVLPGTLIEDAVTTNKYYDFFLSTQIGNEGCFRPTHYYVLHDTWTGKPDSFWPTVTHALTYNFCRSTTTVALPAPVLYAHLAAKRAKETLDGINTYKSVNNIYCDLESFGDLCEVNKDMNVNEKLEGMTFV

>CePrg1

MASGSGRGRGRGSGSNNSGGKDQKYLGTIQPDLFIRQQGQSKTGSSGQPQKCFANFIPIEMTQSDYSIYQYHVEFEPTVDSKANREKMLRDNNVTDEIGHHFVFDGMILYLKEEWEQNQMIEVQHPIDRSLICIRFKQTNRFLVDDPQTINIFNTIIRRSFDALQLTQLGRNYFNWGDSRAVPDYNMSILPGYETAIRMYEENFMLCVENRFKMVREESMYILFHKELRSCQNNPQRVQEKMNEMYGGTTIITRYNNKLHRYTRLDYSISPLSEFVKDGQSITLKEYFKNQYGIEITVDDQPIIISEGKPKQPGEPPQVSYIVPELCFPTGLTDEMRKDFKMMKEIAKHTRMSPQQRLVESRKLIVDLSKNEKVMECFKYWGISLGQDLANVQARVLKSEPLQGKKTYEGKQAEWARGVKECGIYRGSNMTNWIVIGPGSGNSGLLSQKFIEEARRLGKILQVQLGEPMCVPIRGISPNDYLEGVKGAIKQVDGEDIHMLVVMLADDNKTRYDSLKKFLCVECPIPNQCVNLRTLAGKSKDGGENKNLGSIVLKIVLQMICKTGGALWKVNIPLKNTMIVGYDLYHDSTLKGKTVGACVSTTSNDFTQFYSQTRPHENPTQLGNNLTHFVRKALKQYYDSNDQTLPSRLILYRDGAGDGQIPYIKNTEVKLVRDACDAVTDKAAELSNKVQEKIKLAFIIVTKRVNMRILKQGSSLDNAINPQPGTVVDTTVTRPERMDFYLVPQFVNQGTVTPVSYNIIHDDTDLGPDKHQQLAFKLCHLYYNWQGTVRVPAPCQYAHKLAFLTAQSLHDDANGCLRDKLFFL

>CeF55A12.1

MSPHPPQPHPPMPPMPPVTAPPGTMPPMPPVPADAQKLHQITGNDACIKRLQQLKVENGAKMYMKPAEPGQLGRKEVTFTKKGKEDFVVLDRRDKCCNLFFHAVEKNPEFFKMKDGNQIVYDGQSTLYTTVNLFSELDANGTKSKVFQINGADINNDDLKTLPCISLEIYAPRNNSITLSAENLSKRTADQNIEVNNREYTQFLELALNQHCVRETKRFGCFEHGKVYFLNATEEGFDQRDCVDVGDGKQLYPGLKKAIQFIEGPYGRGQNNPSVVIDGMKAAFHKEQTVIQKLYEITGQDPSNGINNMAREKAAAVMKGLDCYSTYTNRKRHLRIEGIFHESASKTRFELPDGKTCSIAEYYADKYKITLQYPNANLVVCKDRGNNNYFPAELMTISRNQRVTIPQQTGNQSQKTTKECAVLPDVRQRIIITGKNAVNISLENELLVALGIKVYSEPLMVEARELDGKELVYQRSVMSEMGKWRAPPGWFVKPAKVPELWALYAVGNQGCRFSSGDPPCETGLFHTDKIMDQLEKVAASGCKYVLVITDDAITHLHKRYKALEQKSRMTIQDMKISKANAVVKDGKRLTLENIINKTNLKLGGLNYTVSDSKKSMTDEQLIIGVGVSAPPAGTKFMMDNKGHLNPQIIGFASNAVANHEFVGDFVLAPSTQDTMASIEDVLQNSIDLFESSRKTLPKRIIIYRSGASEGSHASILAYEIPLARAIIHGYSKDIKLIYIVVTKEHNYRFFRDQLRSGGKATEMNIPPGIVLDNAVTNPACKQFFLNGHTTLQGTAKTPLYTVLADDCAAPMDRLEELTFTLCHYHQIVALSTSIPTPLYVANEYAKRGRDLWGERTVDGPIEAKESHGERLKELTNEIGYKQTDFNEKRINA

>CeT23D8.7

MEDQWLLSAIYDDDLVEKLKVRSSTSSRSTSINVPSLENEFLSSSSGSRVSDDLYLHPIEENREPFKLIGKPLPSTTGRFLSLLANHFQITCNGSIIHQYYIRFDPDIPSKKLNRTILRTLQEQNPGLIECPLVFDGIHTVYSTELINVKEVNNSVINVAGVVNTKESPNLFKLYLTHVDSFLLDTKIITGNQDQNQKLRMMHAIDTVFRQTSTGNFHAVLQSFFSIAQNSAIEPSHGLGWGTVNLGVGREVCYGFYQNVVETFDTLTMNLDVATTTFYRPVALVEFLAEILEVPLATVTDGRSLSDVQKKKFNREVAGLKVETRHCSCPRRFRVARCTWKPTENISFHLSETAGNQDSKPLSLVEYYKRRYNIDLTYKHLPCIEVGRTRECILPLELCYVVSGQRCIKKLNEQQIANLIRATSRNATERQNAVMSLQNRLKMDNDVNAVKFGLKVEAQLLKIEGRVLPVPRLLYRSPNLKRQECVTVPNNGTWDMRGKNFYSGIQIREWAIVCFASPEIIGEASMRSFVRNLVNVASEIGMPFLEEHRFCRYAEPDQTVKLLEHLNEQYNLQLVLCIVPGKSVVYGELKRKGELLGLTTQCVRSQNVSKASPHTLSNLCMKINSKLGGINVILSSPPQSLNSEPVLFIGCHLTRSSLASSSDSTSSIAHCDSSIACLVGSMDGHPTQFSPIFRTQPRHQRTIVDMCEMTREAIINFRKSTGFKPHKIIIYRAGIADVTVDEIMQTELRAVRDACAMIEYGFQPGITFIGLDVTHHTRLFAANEKDRVGNSQNVPAGTLVETGITVNNLFEFYLVSHAGIQGTSRPTKYVVMWDDNSIPSADIHEMTYQLCHTQSRCTRSVSIPSPVYYAKLVAQRAKILMADENFDMERFRLCGIGRNDGMSFT

>CeNrde3

MDLLDKVMGEMGSKPGSTAKKPATSASSTPRTNVWGTAKKPSSQQQPPKPLFTTPGSQQGSLGGRIPKREHTDRTGPDPKRKPLGGLSVPDSFNNFGTFRVQMNAWNLDISKMDERISRIMFRATLVHTDGRRFELSLGVSAFSGDVNRQQRRQAQCLLFRAWFKRNPELFKGMTDPAIAAYDAAETIYVGCSFFDVELTEHVCHLTEADFSPQEWKIVSLISRRSGSTFEIRIKTNPPIYTRGPNALTLENRSELTRIIEAITDQCLHNEKFLLYSSGTFPTKGGDIASPDEVTLIKSGFVKTTKIVDRDGVPDAIMTVDTTKSPFYKDTSLLKFFTAKMDQLTNSGGGPRGHNGGRERRDGGGNSRKYDDRRSPRDGEIDYDERTVSHYQRQFQDERISDGMLNTLKQSLKGLDCQPIHLKDSKANRSIMIDEIHTGTADSVTFEQKLPDGEMKLTSITEYYLQRYNYRLKFPHLPLVTSKRAKCYDFYPMELMSILPGQRIKQSHMTVDIQSYMTGKMSSLPDQHIKQSKLVLTEYLKLGDQPANRQMDAFRVSLKSIQPIVTNAHWLSPPDMKFANNQLYSLNPTRGVRFQTNGKFVMPARVKSVTIINYDKEFNRNVDMFAEGLAKHCSEQGMKFDSRPNSWKKVNLGSSDRRGTKVEIEEAIRNGVTIVFGIIAEKRPDMHDILKYFEEKLGQQTIQISSETADKFMRDHGGKQTIDNVIRKLNPKCGGTNFLIDVPESVGHRVVCNNSAEMRAKLYAKTQFIGFEMSHTGARTRFDIQKVMFDGDPTVVGVAYSLKHSAQLGGFSYFQESRLHKLTNLQEKMQICLNAYEQSSSYLPETVVVYRVGSGEGDYPQIVNEVNEMKLAARKKKHGYNPKFLVICTQRNSHIRVFPEHINERGKSMEQNVKSGTCVDVPGASHGYEEFILCCQTPLIGTVKPTKYTIIVNDCRWSKNEIMNVTYHLAFAHQVSYAPPAIPNVSYAAQNLAKRGHNNYKTHTKLVDMNDYSYRIKEKHEEIISSEEVDDILMRDFIETVSNDLNAMTINGRNFWA

>CeY49F6A.1

MSNQEPYRIPKRTASDPNQSGSQGSCVRPNQEGNVKKEESVNSAPRRMFTLPSSSSTSGAQVFNPIGKCQVQTNSFKIDITKMPKRLLRLSMETRLCGGKTELNLNDGVQAVAGNLNSQEDFTTQEWSQISNYLRKKNAEFKVKISPNGFVYTNGPNSLSDANRQELPRLVEIATSEVLNTPEYLQFGNQTYAVNEPAVSSPDALSEIRSGFDKSVRLVDGENGSTEIIMTIDNKLAPFHKSTSVLKMICDKFEEIVNPARQHRGSQDRRGDRDRGYRGGRSYGHDNRRRSRSRSPQDRGDRGDRGNHHRDHNEDWDERKVKEVERRLLEEPRALGSIADALKGLIVESVHLSKDVNRNSRIKLNEGKNDEQEISVADYYLKQYNYKLKFPYLPMIVSKRFRSETMFPVELLKIVPGQKISIQKINPNVQSAMTGPNSILPSKHVRIVARILESSLKIASNPYLKTFGIRISKDPIKLEAKILSPAQLSFNKYSIMPKPGSVQFEPSREDKFFKPAKIYEVAVIAFRVVSINLSDFCMKLCDLCRRSGLEIRRETRDWIKIENFNPTSTIELKEQMKRLQQSNCSIIIGITEEKKPEVHDVLKYFEAAVGLQTMQIHSKTAGFIISGNGQTAGNVIKKLNLKCGGINYIVEVPQSFNRTVVCSNNSFVQKKLFDGTQFIGFEMTHGAARTLFDKSNGTFDGEPTIVGCAYSLQKATDLGGFNWFQEQNEYRLKNLGTHIPKCLQYYKESSGSLPEKIVIYRTGAGEGDFDRVQQEITEIRSSFAEVQEGYKPSLVVIVANKTSHLRVFPVEIREGDTAMKQNVRSGTCLDGKITSAGREEFVLISQTALISTAKPTKYTIAANDPKWSKNEVMNLTYILSFAHQVCYQPPAVPHILYAADNLAKRGRNNFIQHKKLGELSKTIQATLTKYSDLNIEMDSKELSGELVEDITKSMNVMAIKARNFWA

>CeZK1248.7

MFIDACKKRGMIIKPPSETSLCHMDNIISLLENAAASKCKFAFVITDDSITHLHKKYKALEQKSMMVIQDMKISKANSVVKDGKRLTLENVINKTNMKLGGLNYTVSDAKKSMTDEQLIIGVGVSAPPAGTKYMMDNKGHLNPQIIGFASNAVANHEFVGDFVLAPSGQDTMASIEDVLKSSIDLFEMNRNTLPKRIIIYRSGASDGSHASILAYEIPLARATIQGYSKDINLIYIIVTKEHSYRFFRDQLRSGGKATEMNIPPGIVLDSAVTNPACKQFFLNGHTTLQGTAKTPLYTILADDCNAPMDRLEELTYTLCHHHQIVALSTSIPTPLYVANEYAKRGRDLWSEKMDVSFFQCQYH

>CeZK218.8

MDKSHVSYKPPTTRLGLYPFVSATGCVLVVDTIKIHDILLKTVNFEKGLHYVNAGTIEYMASEYNYTIKIKNHFVYSLAPELSKKNPKVKQSETPRFINPPSGTVVDKLVVSGYKFDFYLNSHHAVLGTSRPAHYTVMYDDMGMSQDEVYKMTDALAFLSARCRKPISLPAPVHFYVYKLFQCWP

>CePrg2

MIQNDYSIYQYHVEFEPTVDSKATRENMLRQPSVTVEIGKHFVFDGMILYLKEEWDQNQMIEVQHPNDNSLICIRFKKTNRFLVDDPQTINIFNTIIRRSFDAMKLTQIGRNYFDWDNSRALRKELRSCQNNRQRVQEKMNEVYGGSTIITRYNNKLHRFTRLDNEITPLSKFQKDGEQIILKEYFKNQYDIDITDDEQFIIISEGKPKQPGEPPQVNYIVPELCFPTGLTDEMRKDFKMMKEIAKHTRMSPQQRLDETRKLITKLSQNQTMMECFQYWGISLGQDLANVQARVLKSEPLQGKRQYEGKQAEWARGVKECGIYRGSNMTNWIVIGPGSGNSGLLAQKFIAEARNLGRTLQVQLGEPMCVKINGISPNDYLEGLKAAIKSVDGEEIHMLVVMLADDNKTRYDSLKKYLCVECPIPNQCVNLRTLAGKSKDGGENKNLGSIVLKIVLQMICKTGGALWKVNIPLKSTMIVGYDLYHDSTLKGKTVGACVSTTSNDFTQFYSQTRPHENPTQLGNNLTHFVRKSLKQYYDNNDKTLPSRLILYRDGAGDGQIPYIKNTEVKLVRDACDAVTDKAAELSNKVQEKIKLAFIIVTKRVNMRILKQGSSSKSAINPQPGTVVDTTVTRPERMDFYLVPQFVNQGTVTPVSYNIIHDDTGLGPDKHQQLAFKLCHLYYNWQGTVRVPAPCQYAHKLAFLTAQSLHDDANGYLRDKLFFL

>CeC14B1.7

MDDVLDRIMGSAPSQSGSSLNRTPKRDHRSNPDMHEPSSKRSAPMFTTPKSATPIGRDSFATLDTLNVQMNMFTMDIKDMPHKLQRLQVDVIICSSNGKQINVNLGVLAAKGDVNSHNRRLAQFFIMRAVHDKLPEKFSGKSHHFLAYDCAATLYVPEGVYTGDAEEQVTLKIDDFPKDEWKIVSKLSRRKDDSYLVTLKPAGFVHTQGEHARAEANRMELTRCVEIITSQMLNNEDYYQFANCPNREAVAVDEVVTAEASPEIHVEVTVAAVLIQEIIVEVTATEVERYRDESRGCRDMNDSRRDSSNGVDYSPSDAAEVEKALGERGSTKRFIEDALKGLDVECTRLKGNLIRVCSIAENNAENTSFMMKGDKGEQEITVVQYFQLQYNIKLKHSRLPLVVSKRFKHESFFPMELLQIAPGQRIKVNKMSPTVQSSMTGMNASMPEQHVKLVQNILIDNLKLKQSKYMDAFGIRLMSTKPLQLPAKLLPPAQIKFKNQTYMPEMSRPAFRTQDKFVQPAHIGRIGVVVFDNCIQMRQAESFCDNLAAICRDNGIKIDQDSRDWTICEINSSDAAGIQNRMRKWADDKVDILFAITREKKPDVHDILKYYEESIGLQTIQVCQQTVDKMMGGGGGRQTIDNVMRKFNLKCGGTNFFDEIPSSVRGRAVCSNSETLSKKLFEHVQFIGFEISHGAARTLFDRSRSQLDGEPSVVGVSYSLTNSTQLGGFTYLQTQKEYKLQKLGEVFPKCVQSYKEHSRRLPTRIVIYRVGAGEGDFKRVKEEIEEIRGTFDKIQPGYRPHLVVVIAQIASHARVFPSNITGRKAMEQNVPSGTCVDNVPTSYGYDEFILSSRTPLIGTVRPCKYTILINDANWSKNEIMHLTYFRAFGHQVSYKPPSVPDVLYAAENLAKRGRNNYKIHQRHVNLQAVENSIIGDHSELINEDMREELAAAIVDHMSVAMNGMTISKRNFWA

>ERI-7

MIEADKKFLELVKKNQKQNTNPVYQYTLEDNPESLTLQDVLESHMTDPIELNSFDSMEKEAGIPFSNEKQRTAIKMALNENRKLVCIQGPPGTGKTFTLTLLLCRLIQQKKQVVVLAPTREALANIRMMTKKTLKRMGIKVHEHALMDTNEYRDVINKSDRALMAAEEVRDLRKAFDNGEITENVLDEMRQSIINRVRNEVGAEVIGNVRVAFATIGASFVDFVMKHKKFDPCLCIIDEAAQVMEAQTWPAVYKMKRIVMAGDPKQLPALVFTDEAKAFGLQNSVMDRILEKKNNFSWIMLENQYRSNAKIATWSNTCFYHNQLKTDVKCHEYSLHTILNPQPKKFRNLFDPLVLIDTSLERDVEKRLETYEHAVFDTNSINKTKQGFSYANLAEAKIAIGHYQRLLKYGVQPSDIAIITPYKGQTSLVTKLMEEFGAETGYTDFVQTTIGTVDSVQGKEYEVVIFTMVRSNPRKTMGFVSELRRLNVVITRAKRHFMFIGNGYLLAESKKDEIRKLYDCFKNAKRRFHPNNAFGEDGNVTDYVSNNFGQDLESFMNYSNDEEMINWCKRFNENGPDYRAKKALAWEKREETRLMKTISKVKYEYKRGSTEQLASELDQLDCSSN

>ERI-6, isoform a

MTDKENFYSGFFLGRLPGSFDSRFLIKVACFQPGKEERNVFELTTDTKREEDYGEMVHFWVDKGPLQNKSFVRFEENYETFFIHENKMFDRIVHTDKDFYVGVNDLPDSDIYIHPDLGKMIDTRSIVTPGKKYNFITFEVKQNENNSFDIEIVGGEVQGKEKNSEEPSAKEKIIQKMEQVHEVMKVEYEYNKNEETANWDVIGHLKLIKFYDKDLTSGLRPVLRLMDNRHVALFKKQRETMPIKMIKVIEVDGIWKDDVNFVPQEGTLLPTDHDEKTVVLLGNDYRHKFERLFKREDVFRLESNNTLTHLKFWQYGLEKI
